# Examining the robustness of observational associations to model, measurement and sampling uncertainty with the vibration of effects framework

**DOI:** 10.1093/ije/dyaa164

**Published:** 2020-11-05

**Authors:** Simon Klau, Sabine Hoffmann, Chirag J Patel, John PA Ioannidis, Anne-Laure Boulesteix

**Affiliations:** 1 Institute for Medical Information Processing, Biometry, and Epidemiology, Ludwig-Maximilians-Universität München, Munich, Germany; 2 Leibniz Institute for Prevention Research and Epidemiology–BIPS, Bremen, Germany; 3 LMU Open Science Center, Ludwig-Maximilians-Universität München, Munich, Germany; 4 Department of Biomedical Informatics, Harvard Medical School, Boston, MA, USA; 5 Meta-Research Innovation Center at Stanford (METRICS), Stanford University, Stanford, CA, USA; 6 Department of Epidemiology and Population Health, Stanford University School of Medicine, Stanford, CA, USA; 7 Department of Biomedical Data Science, Stanford University School of Medicine, Stanford, CA, USA; 8 Department of Statistics, Stanford University School of Humanities and Sciences, Stanford, CA, USA; 9 Department of Medicine, Stanford University School of Medicine, Stanford, CA, USA

**Keywords:** Measurement error, metascience, observational study, replicability, researcher degrees of freedom, stability

## Abstract

**Background:**

The results of studies on observational associations may vary depending on the study design and analysis choices as well as due to measurement error. It is important to understand the relative contribution of different factors towards generating variable results, including low sample sizes, researchers’ flexibility in model choices, and measurement error in variables of interest and adjustment variables.

**Methods:**

We define sampling, model and measurement uncertainty, and extend the concept of vibration of effects in order to study these three types of uncertainty in a common framework. In a practical application, we examine these types of uncertainty in a Cox model using data from the National Health and Nutrition Examination Survey. In addition, we analyse the behaviour of sampling, model and measurement uncertainty for varying sample sizes in a simulation study.

**Results:**

All types of uncertainty are associated with a potentially large variability in effect estimates. Measurement error in the variable of interest attenuates the true effect in most cases, but can occasionally lead to overestimation. When we consider measurement error in both the variable of interest and adjustment variables, the vibration of effects are even less predictable as both systematic under- and over-estimation of the true effect can be observed. The results on simulated data show that measurement and model vibration remain non-negligible even for large sample sizes.

**Conclusion:**

Sampling, model and measurement uncertainty can have important consequences for the stability of observational associations. We recommend systematically studying and reporting these types of uncertainty, and comparing them in a common framework.

**Key Messages**

- We extended the concept of vibration of effects such that model, sampling and measurement uncertainty can be compared in a common framework.
- Model choices, sub-sampling and measurement error are associated with a large variability in the effect estimate when studying observational associations for data from the National Health and Nutrition Examination Survey (NHANES). Measurement error can also lead to substantial bias. The consequences of model and measurement uncertainty remain non-negligible for large sample sizes.
- The framework can be used to systematically compare these main sources of uncertainty in observational associations with the aim of improving the transparency and quality of research results.

## Introduction

A large part of observational studies in epidemiology is concerned with aetiological research questions, i.e. the study of associations with the aim of uncovering underlying causes of disease. Observational associations in aetiological epidemiology can be unstable and occasionally difficult to replicate in subsequent studies.^1–4^ The instability sometimes leads to contradictory findings from similar epidemiological studies, raising challenges to the interpretation and credibility of epidemiological evidence.^5^

There are many factors that contribute to this instability. Small sample sizes may lead to high instability in the estimates of the magnitude of an association and its statistical significance. Another key factor that may play an important role in the instability of research findings in aetiological epidemiology includes diverse model specification choices, such as which variables are adjusted for. As we have shown in earlier research, the inclusion and exclusion of potential adjustment variables can cause a large variability in results when estimating the association between an exposure and an outcome variable of interest using a given data set.^6^ Finally, measurement error in exposure and outcome variables may further exacerbate the instability of observational associations. Note that these factors have a more major impact on aetiological epidemiology, whereas, when the primary research goal of an epidemiological study is description or prediction, they may play a less prominent role.

While sampling uncertainty is classically accounted for when deriving *P*-values and confidence intervals to report the results of epidemiological studies, methods to account for model and measurement uncertainty are not commonly used when analysing observational data. Instead, results are sometimes presented as if the chosen model were the only possible model, even though different authors may consider very different sets of adjustment variables when analysing the same research question of interest.^6^^,^^7^ The large majority of observational analyses are not pre-registered and do not have explicitly pre-specified analysis plans.^8^ Concerning measurement error, there is a widespread and persistent belief that the effects of exposure measurement error and exposure misclassification are relatively benign, as they will merely result in a bias in parameter estimates towards the null and loss in statistical power.^9–11^ However, these presumed consequences of exposure measurement error and exposure misclassification, which are sometimes mentioned in the discussion of epidemiological findings to argue that an observed association may potentially have been underestimated, only hold in cases where the variable of interest is the only covariate in the model that is measured with error. If the included adjustment variables are also subject to measurement error, which is almost always the case in epidemiological studies, it is more difficult to predict whether measurement error will attenuate or inflate risk estimates.^12–15^

Due to the multiplicity of possible analysis strategies, the relatively small sample sizes of many epidemiological studies and the ubiquity of measurement error, model, sampling and measurement uncertainty all appear to play important roles in the instability of observational associations and may contribute to the non-replicability of research findings. It would be interesting to quantify and compare these different sources of uncertainty in a common framework.

The aim of this work is to extend the vibration of effects approach,^7^ which we previously used to assess model and sampling uncertainty,^6^^,^^16^ to measurement uncertainty in order to provide a tool to investigate the robustness of observational associations to these three types of uncertainty.

We will illustrate this approach with data from the National Health and Nutrition Examination Survey (NHANES) and consider three different scenarios for measurement vibration. In the first scenario, we introduce measurement error only in the exposure of interest. This type of error is expected to reduce the strength of the association. Secondly, we introduce measurement error only in the adjustment variables. This second scenario occurs in practice when there are special efforts being made to reduce measurement error to a minimum for the exposure of interest or if a method for measurement error correction has been applied to account for measurement error in this variable. Finally, we consider a more realistic scenario for measurement error where error is present both in the variable of interest and in the adjustment variables. Additionally, we compare measurement vibration with model vibration and sampling vibration. We complement the analyses on real data with results on simulated data to investigate the behaviour of the three types of vibration for increasing sample sizes.

## Methods

### Model and sampling vibration

We previously introduced the concept of vibration of effects to quantify the variability in results when studying an association of interest under a broad range of model specifications.^7^ The idea of this approach is to quantify the variability of results through a vibration ratio, which we defined as the ratio of the largest vs the smallest effect estimate for the same association of interest under different analysis choices. Moreover, we applied this framework to assess the vibration of effects arising through the specification of the probability model to data from the NHANES.^6^ We showed that this type of vibration, which we obtained through the inclusion or exclusion of all potential adjustment variables, can have important consequences for the estimation of the effect of the variable of interest on all-cause mortality in a Cox regression. The vibration ratios used were the relative hazard ratio (RHR) and the relative *P*-value (RP). In this second study, these vibration ratios describe the ratio of the 99th and 1st percentile of hazard ratios and the difference between the 99th and 1st percentile of −log10(*P*-value), respectively. In addition, we suggested showing volcano plots with *P*-values at the *y*-axis and effect estimates at the *x*-axis. These volcano plots allow easy detection of patterns like the Janus pattern, which is characterised by significant estimates in both a positive and negative direction.

The vibration of effects framework can be used to transparently report the multiplicity of results arising from different model specifications. From a statistical perspective, fitting different models to estimate and test an effect of interest results in a multiple testing problem. Researchers may be tempted to selectively report the most spectacular of these results [i.e. the smallest *P*-value(s) or the larger effect(s)], which are often type 1 errors. It is then likely that later replication of these results on independent data will fail. In this worrying context, reporting the vibration of effects and showing volcano plots^6^^,^^7^ rather than one or a few model fit(s) is a valuable alternative reporting strategy in order to reduce non-replicable findings and increase transparency regarding the multiplicity of possible modelling strategies.

Furthermore, we previously applied the vibration of effects framework when fitting the same model on different subsamples of the data,^16^ and compared this type of vibration, denoted as ‘sampling vibration’ in the following, with ‘model vibration’ as assessed in Patel et al.^6^ When studying sampling vibration, a favourite model has to be chosen from all models considered in the assessment of model vibration. For this model, we suggested drawing a large number of *B* random subsets of the data and fitting the same statistical model on each of these subsets.^16^ Similar to model vibration, vibration ratios and volcano plots can be used to illustrate sampling vibration.

### Measurement vibration

In this work, we suggest further extending the vibration of effects framework to illustrate measurement uncertainty. For continuous variables, we focus on an additive classical non-differential measurement error model *Z *=* X *+* U*, where *Z* is the observed exposure, *X* is the true exposure, and *U* is a measurement error term, which is independent of the true exposure *X*. Measurement error for a continuous variable can be assessed by quantifying the correlation *ρ_XZ_* between true exposure *X* and observed exposure *Z* in a validation sample. For binary variables, the magnitude of misclassification can be quantified through sensitivity and specificity values.

In order to study the impact of measurement error following the classical measurement error model *Z = X + U* for continuous variables for a given data set, we have to introduce an error term that is independent of the true exposure values *X* to generate observed exposure values *Z*. As these true exposure values are unknown for any given data set, we will assume true exposure *X* to be equal to the original recordings of the variables.^11^^,^^17^ We can generate virtual error-prone observed values *Z* for continuous variables based on a given correlation *ρ_XZ_* as follows. As shown in the Supplementary data, available at *IJE* online, we first calculate the variance of observed exposure *Z* as the variance of true exposure *X* divided by *ρ^2^_XZ_*. We can then determine the measurement error variance by subtracting the variance of *X* from the variance of *Z*:

(1) $$\text{Var}(U)=\frac{\text{Var}(X)}{{\rho^{2}}_{XZ}}-\text{Var}(X)$$

As a final step, to obtain observed exposure *Z*, measurement error values *U* can be generated from a normal distribution with mean zero and variance Var(*U*), and added to the true exposure *X*.

Furthermore, we suggest adding exposure misclassification to binary variables by using values for sensitivity and specificity. In particular, for a binary variable with observed values 0 or 1, all values of 1 can be replaced by random values from a binomial distribution with a probability of success that is equal to the sensitivity. Similarly, all values of 0 can be replaced by random values from a binomial distribution with a probability of success equal to 1− specificity. As shown in the Supplementary data, available at *IJE* online, for ordinal variables, we follow a strategy that is similar to the simulation strategy for continuous variables by assuming latent variables that follow a normal distribution.

Similarly to sampling vibration, we have to choose a favourite model among the models that are considered in the assessment of model vibration. For this model, we repeat the procedure of adding random measurement error *B* times. With *B* different results obtained by adding measurement error to the variables, the vibration of effects framework can be used. To quantify the results, we suggest using the 99th and 1st percentiles of effect estimates and *P*-values to construct vibration ratios to define relative effect estimates and RPs , similar to model and sampling vibration. Moreover, these results can be visualized with volcano plots.

### The NHANES cohort data

#### Data set description

We analyse cohorts from the NHANES, modelling all-cause mortality with a variable of interest and 15 adjustment variables (for more details on data collection and pre-processing see Patel et al.^6^). For this work, we run the analyses successively with 30 variables of interest, which were chosen from a pool of 417 variables. We selected these 30 variables because of a small amount of missing values (*<*15%), and, for ease of interpretation, ensured that they were either binary or continuous. For illustrative purposes, out of these 30 variables, we will limit the presentation of results to 2 continuous variables of interest (thigh circumference and high-density lipoprotein (HDL)-cholesterol), as well as the 2 binary variables diabetes (defined as self-reported doctor diagnosed diabetes and fasting glucose *>*125 mg/dl) and heart disease (defined as self-reported doctor diagnosed heart attack or coronary disease). Results for the other 26 variables of interest can be found in the Supplementary data, available at *IJE* online. The 15 adjustment variables used were selected in line with our recent work.^6^ They comprise variables of continuous, binary and ordinal type.

#### Assessing model and sampling vibration

In order to assess model vibration for the NHANES data, we follow Patel et al.,^6^ where we focused on the particular type of model vibration that is due to the inclusion or exclusion of all potential adjustment variables in a Cox regression. Furthermore, we include the variables age and sex as baseline variables in every model. The combination of the 13 remaining adjustment variables yields 2^13^ = 8192 different models. For the investigation of sampling vibration, we consider *B *=* *1000 subsets of size 0.5*n*, where *n* is the number of observations. Moreover, we use the model with all 15 adjustment variables as the favourite model.

#### Assessing measurement vibration

In order to assess the vibration of effects due to measurement uncertainty in the NHANES data, we first have to get an idea of the magnitude of measurement error that we can expect in this study. Ideally, the magnitude of measurement error should be assessed in a validation study, in which the error-prone variables and a gold standard are both assessed to study the measurement error characteristics specific to the NHANES data. In this validation study, it would also be possible to assess the correlation between the measurement errors in the variable of interest and in all adjustment variables. In the absence of such a validation study, we decided to search in the literature for information on the precision with which the variables of interest and adjustment variables used in our analyses are typically measured and assumed the correlation between the measurement errors in the different variables to be zero. To obtain a representative range of measurement error, we aimed to collect high and low values of sensitivity, specificity and correlations for each variable. As we found only scarce information for most variables, we decided to calculate average values for sensitivity, specificity and correlations for high and low measurement error to obtain representative values which we applied to all error-prone variables. For more detailed information for the different variables and references see Supplementary Tables 2 and 3, available as Supplementary data at *IJE* online. Using the average values for high and low measurement error as limits in a uniform distribution, we randomly draw a correlation and values for sensitivity and specificity for each iteration *b *=* *1*,..,B*. In the case of continuous variables, this strategy resulted in correlation coefficients between observed exposure and true exposure uniformly distributed between 0.73 and 0.9. For binary variables, we draw values for sensitivity and specificity from a uniform distribution between 0.56 and 0.85, and between 0.73 and 0.98, respectively. Finally, we generate measurement error for different types of variables following the procedure described in the section Measurement vibration. Similar to the assessment of sampling vibration, we use the model with all 15 adjustment variables as a favourite model and repeat the procedure *B *=* *1000 times. In accordance with Brakenhoff et al.,^11^ we assume the variables age and sex to be without measurement error, and the same is assumed to apply to race/ethnicity.

#### Comparing different scenarios of measurement vibration with sampling and model vibration

In the assessment of measurement vibration for the NHANES data, we distinguish between three different scenarios: 1) We add measurement error to the variable of interest but not to the adjustment variables, or, conversely, 2) we add measurement error to all adjustment variables except age, sex, and race/ethnicity, and consider the variable of interest to be measured without error, and 3) we add measurement error to both the variable of interest and the adjustment variables (expect age, sex and race/ethnicity). For all scenarios, we assume that information on the outcome has no measurement error, an assumption that is justifiable given the completeness and accuracy of NHANES data on death ascertainment. Finally, we compare these three scenarios, which illustrate measurement vibration, with model and sampling vibration, and focus on the interpretation of results on RHRs and volcano plots. In these volcano plots, we consider a *P*-value *<* 0.05 as significant. For all analyses on the NHANES data, we use the *coxph* function from the R-package *survival*. Due to the complex sampling structure of the NHANES data, we account for participant weights, as well as for the clusters pseudostrata and pseudosampling units by using a robust sandwich variance estimator. For all types of vibration, we standardise the continuous variables of interest to ensure comparability.

### Simulation study

In addition to the analyses on real data, we conduct a simulation study with the aim of comparing measurement, sampling and model vibration for sample sizes that can both be smaller and larger than the initial sample size of the NHANES data. In this simulation study, we generate data with sample sizes *n* ∈{500, 1000, 5000, 10 000, 50 000, 100 000, 200 000}. The simulated data is based on the NHANES data in the sense that we adopt the correlation structure as well as the effect sizes of the variables on the real data. More details about the data generation are described in the Supplementary data, available at *IJE* online. Finally, we assess the three types of vibration in the same way as introduced in the section The NHANES cohort data. For measurement vibration, we consider only the scenario with measurement error in both the variable of interest and the adjustment variables.

## Results

### Results on the NHANES data

**Figure 1:**
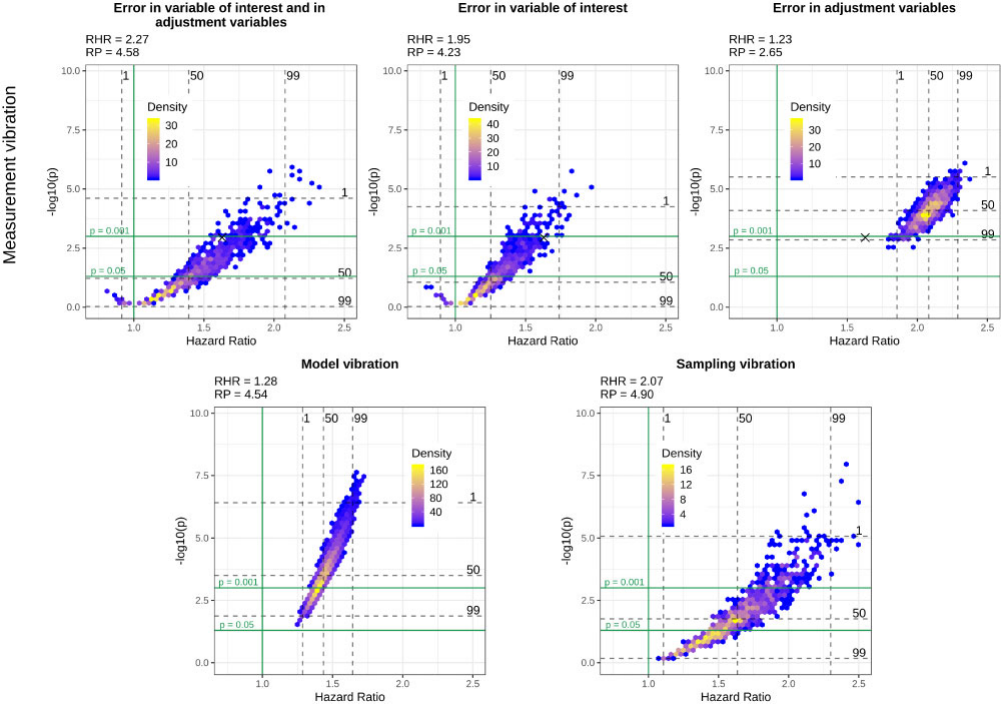
Volcano plots for different types of vibration and different scenarios of measurement vibration when diabetes is the variable of interest. The black cross in the top panel indicates the model without measurement error

**Figure 2:**
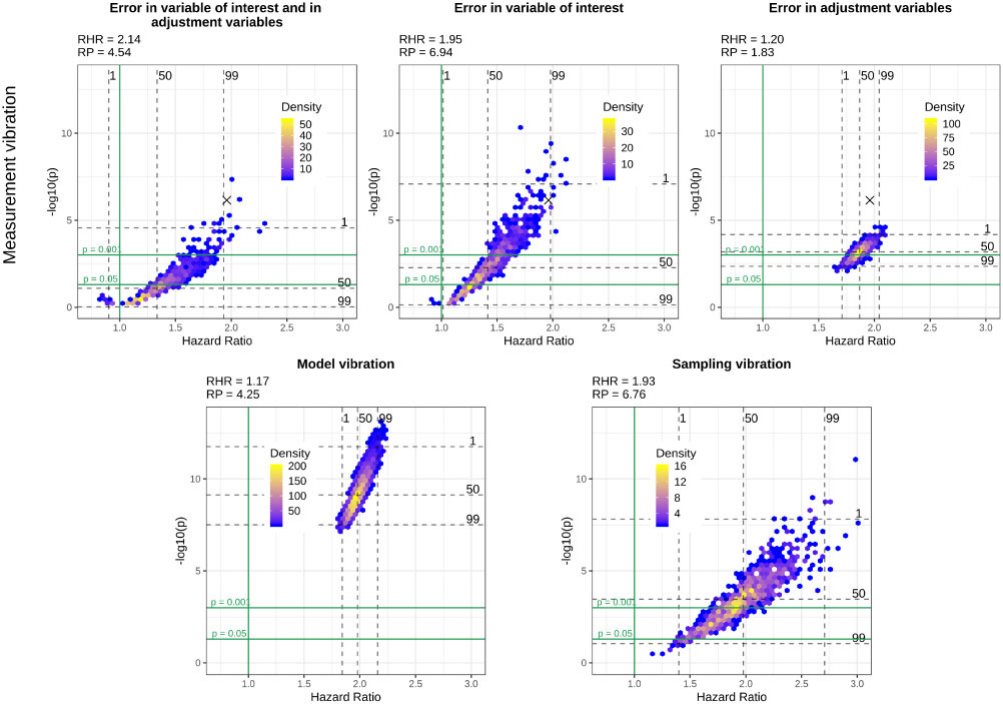
Volcano plots for different types of vibration and different scenarios of measurement vibration when heart disease is the variable of interest. The black cross in the top panel indicates the model without measurement error

**Figure 3:**
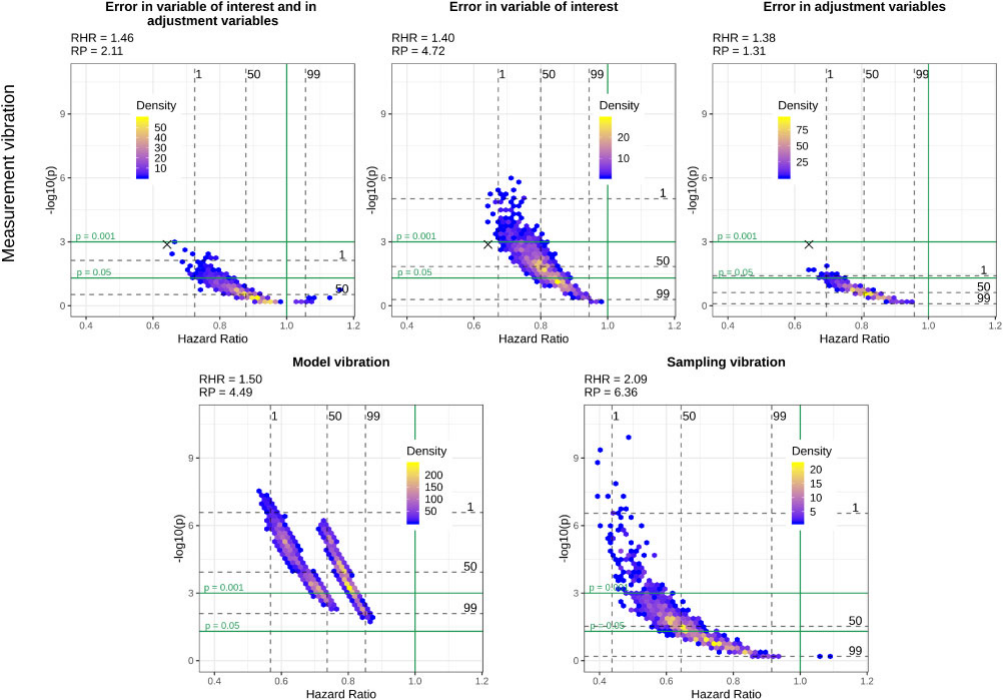
Volcano plots for different types of vibration and different scenarios of measurement vibration when thigh circumference is the variable of interest. The black cross in the top panel indicates the model without measurement error

**Figure 4:**
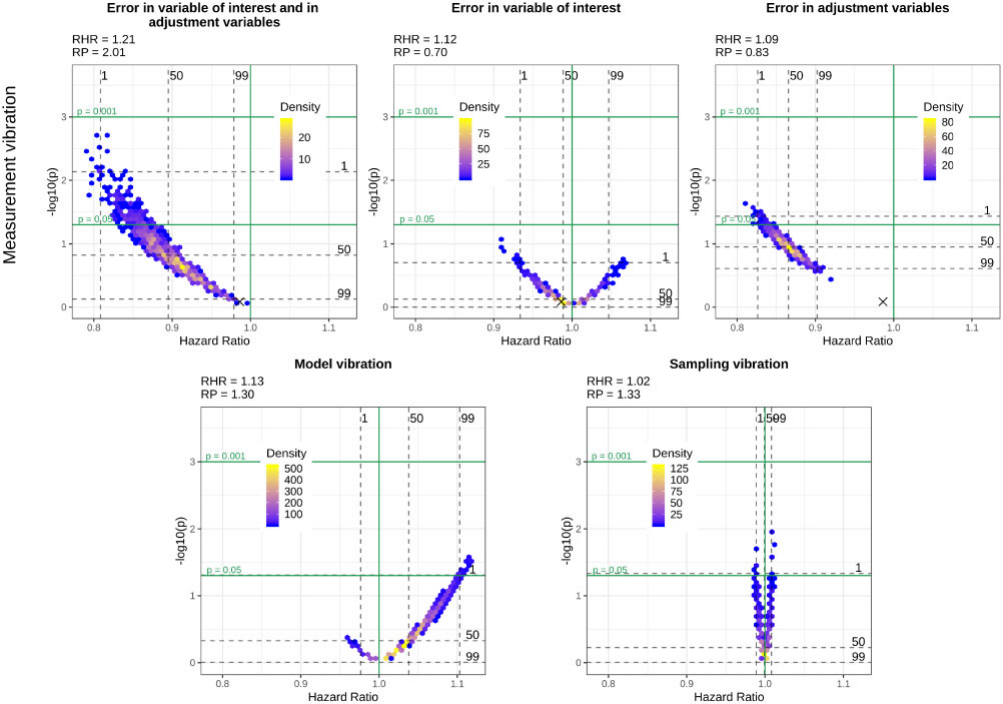
Volcano plots for different types of vibration and different scenarios of measurement vibration when HDL-cholesterol is the variable of interest. The black cross in the top panel indicates the model without measurement error

Figures 1–4 show volcano plots of model, sampling and measurement vibration for the three different scenarios of measurement error, introduced in the section Comparing different scenarios of measurement vibration with sampling and model vibration, for the four selected variables of interest, i.e. diabetes, heart disease, thigh circumference and HDL-cholesterol. In these figures, we provide additional quantitative information about RHRs and RPs.

In the most realistic scenario for measurement error, i.e. when there is measurement error in the variable of interest and the adjustment variables, both significant and non-significant results can be observed for all variables of interest. Measurement vibration in this scenario is higher than model and sampling vibration in terms of RHRs for three of four variables of interest (diabetes, heart disease and HDL-cholesterol). In the assessment of sampling vibration, both significant and non-significant results are obtained for all variables of interest and sampling vibration is higher than model vibration for diabetes, heart disease and thigh circumference. In contrast to measurement and sampling uncertainty, model uncertainty does not change the significance of results for diabetes, heart disease and thigh circumference, where all results are significant. Only for HDL-cholesterol does model uncertainty change the significance of results. Whereas we observe a Janus pattern for HDL-cholesterol in the case of sampling vibration, we can clearly distinguish two clusters for thigh circumference in the case of model vibration. These clusters result from the choice of whether the body mass index was included or excluded as an adjustment variable.

Despite a general tendency of measurement error to lead to an attenuation in effect estimates and loss of statistical power when present only in the variable of interest, we can also observe cases where measurement error leads to an inflated effect estimate and a smaller *P*-value compared with the results without measurement error in this scenario. This tendency is particularly evident for HDL-cholesterol and diabetes and can also be observed for the large majority of the variables of interest illustrated in the Supplementary data, available at *IJE* online. When measurement error is only present in the adjustment variables, we can observe a clear bias towards the null for thigh circumference, whereas there is a substantial bias away from the null for diabetes and HDL-cholesterol. Finally, in the more realistic scenario when measurement error is present both in the variable of interest and in the adjustment variables, the effects of measurement error are more difficult to summarise as they seem to combine the effects of a general attenuation towards the null, which occurs due to the measurement error in the variable of interest, and the effect attenuation or inflation that occurs due to measurement error in the adjustment variables.

### Results on simulated data

**Figure 5:**
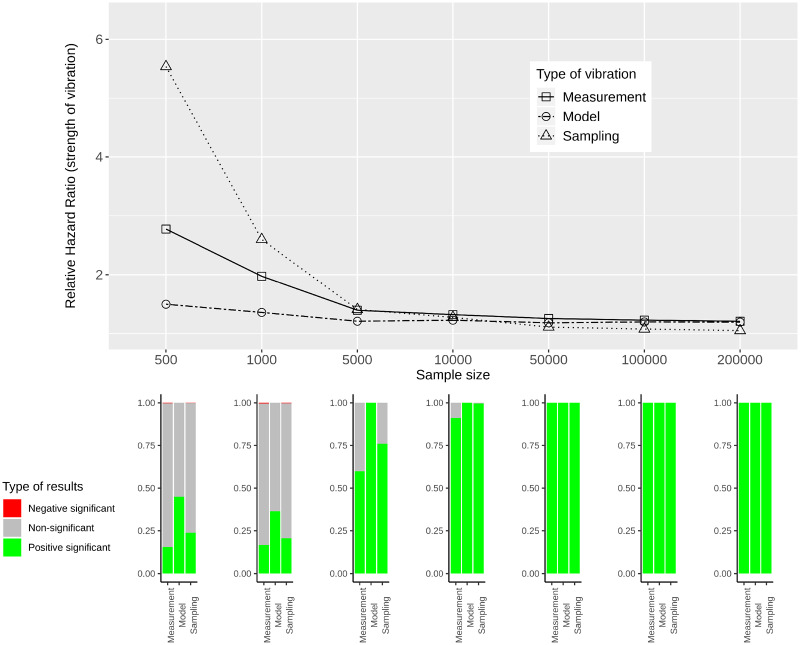
Measurement, model and sampling vibration for different sample sizes (top panel), and bar plots showing the type of results in terms of significance of estimated effects (bottom panel) for the association of diabetes with mortality

**Figure 6:**
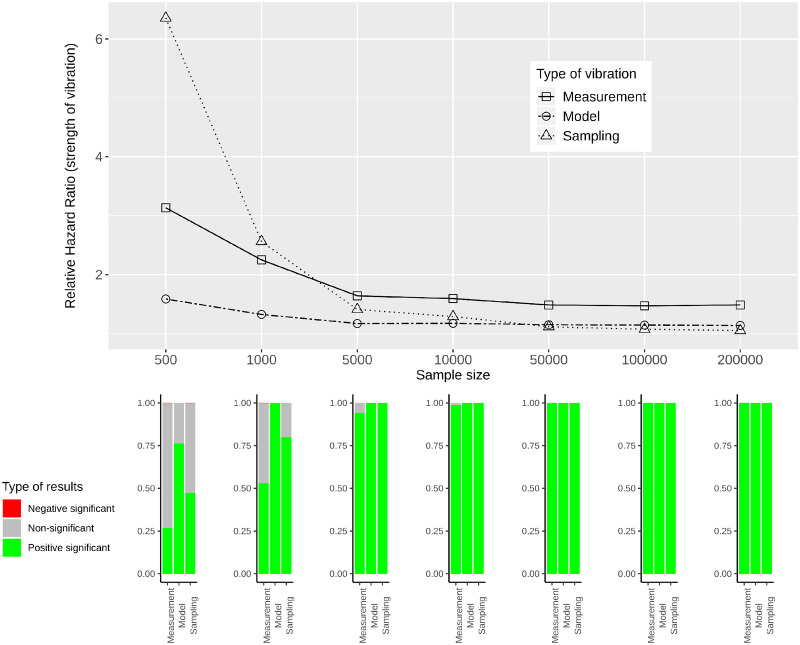
Measurement, model and sampling vibration for different sample sizes (top panel), and bar plots showing the type of results in terms of significance of estimated effects (bottom panel) for the association of heart disease with mortality

**Figure 7:**
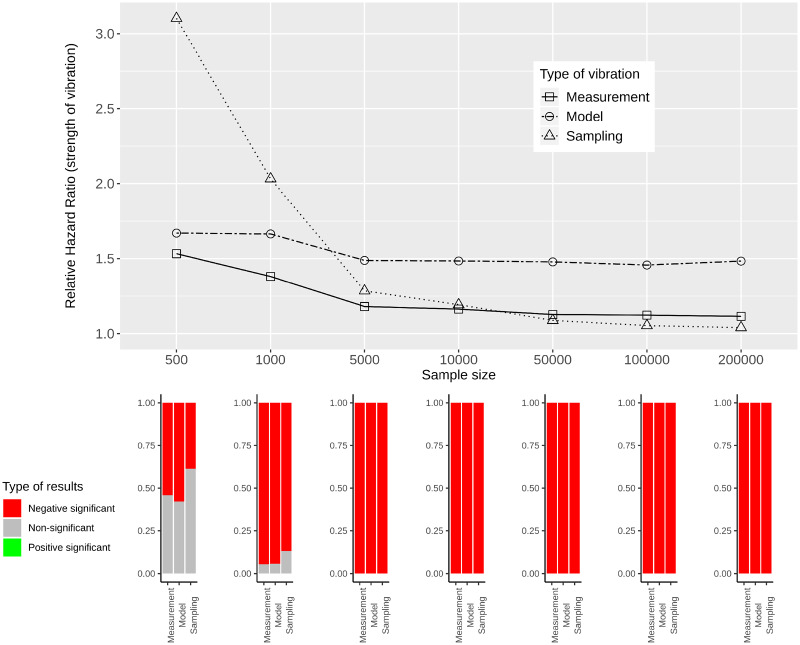
Measurement, model and sampling vibration for different sample sizes (top panel), and bar plots showing the type of results in terms of significance of estimated effects (bottom panel) for the association of thigh circumference with mortality

**Figure 8:**
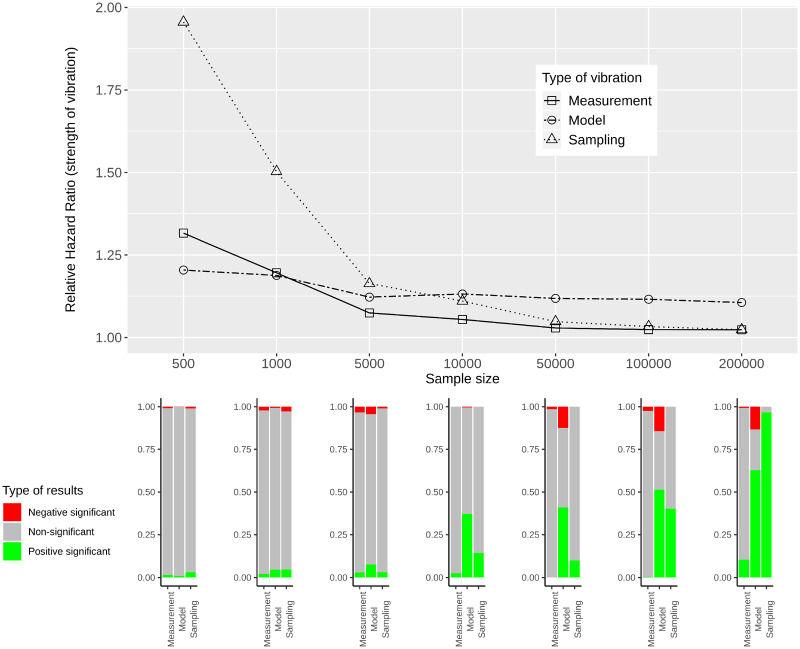
Measurement, model and sampling vibration for different sample sizes (top panel), and bar plots showing the type of results in terms of significance of estimated effects (bottom panel) for the association of HDL-cholesterol with mortality

Figures 5–8 provide RHRs quantifying the variability in effect estimates for simulated data of varying sample sizes. In the lower panels of these figures, bar plots show the percentage of significant results for each sample size and each type of vibration for the three categories: negative significant, non-significant and positive significant.

For all variables of interest, RHRs decrease with increasing sample size. This is most obvious for sampling vibration, which is larger than model and measurement vibration for small sample sizes and tends to 1 with increasing sample size. Model and measurement vibration, on the other hand, remain non-negligible even for a sample size of 200 000. For diabetes, heart disease and thigh circumference RHRs *>* 1.1 can be observed. For HDL-cholesterol, model vibration decreases to 1.1 and measurement vibration to 1.02 for the largest sample size. In the comparison of model and measurement vibration, measurement vibration is lower for thigh circumference and higher for diabetes and heart disease for all sample sizes. For HDL-cholesterol, measurement vibration is higher than model vibration for small sample sizes, and lower for large sample sizes.

When focusing on the results with regard to the type of significance, both significant and non-significant results are present for small sample sizes and all types of vibration for the three variables diabetes, heart disease and thigh circumference. For large sample sizes, the results indicate significance with either only positive sign or only negative sign (without showing a Janus pattern). For HDL-cholesterol, in contrast, a Janus pattern can be observed for measurement and model vibration for both small and large sample sizes. For sampling vibration, most of the results are significant with positive sign for the largest sample size, but non-significant results occur as well. As shown in the Supplementary data, available at *IJE* online, 8 of the other 26 variables of interest can be associated with a Janus pattern for at least one type of vibration for the largest sample size.

## Discussion

In this work, the vibration of effects approach,^7^ which we previously used to assess the variability in observational associations for different model specifications,^6^ and applied to different subsamples of the data,^16^ was extended to exposure measurement uncertainty. Through this extension, it is possible to quantify and compare model, sampling and measurement uncertainty in a common framework when investigating the stability of research findings in observational studies. We studied these three sources of uncertainty on real data for different scenarios of measurement vibration and on simulated data for varying sample sizes. In accordance with Loken and Gelman^18^ and in contrast to what is commonly assumed in the literature,^9^^,^^10^ we found in our analyses on the NHANES data set that even in the simple situation where there is only measurement error in the variable of interest, measurement error can lead to occasional overestimations of parameter estimates. This phenomenon was well-illustrated by Loken and Gelman^18^ and especially occurs in the situation of low sample sizes. Yet, even for larger sample sizes, the additional variance in the estimator, which is introduced by measurement error, can induce overestimations of parameter estimates.

For the more realistic scenario of measurement error, where both the variable of interest and the adjustment variables were assumed to be prone to measurement error, measurement vibration was even less predictable as both bias towards the null and systematic inflations of effect estimates occurred in this situation. For this latter scenario, measurement vibration, as quantified through RHRs, exceeded model vibration and sampling vibration for 27 and 12 of the 30 associations of interest that we studied, respectively. In our simulation study we found that, while all types of uncertainty decreased for increasing sample sizes, model and measurement vibration persisted non-negligibly for large sample sizes in contrast to sampling vibration.

For most probability models, there are theoretical results on the behaviour of sampling uncertainty. In contrast, the consequences of model and measurement uncertainty on parameter estimates in observational studies in epidemiology are very difficult to predict. Model uncertainty is, in principle, reducible by considering the fit of the different candidate models to the data (note, however, that there are different possible ways to do that, implying some sort of method uncertainty). In contrast, a reduction in sampling uncertainty and measurement uncertainty requires more effort at the data collection stage as it can only be achieved by increasing the sample size or by using more precise measurement tools, respectively. Finally, in the comparison between the different types of vibration, one must keep in mind that measurement uncertainty does not only lead to a variability in effect estimates, but also to bias.

Measurement error may also be a prominent feature for outcomes assessed in observational studies. This was not an issue for the mortality outcome that we used in the NHANES analyses, but measurement error in the outcome may be as large as or even larger than measurement error in the exposure and adjustment variables in many other circumstances. In these cases, a similar approach can be used to investigate the vibration of effects due to outcome measurement error. Similarly, while we focused on additive classical measurement error in this work, it is straightforward to extend the concept of measurement vibration to other error structures including systematic, multiplicative and heteroscedastic measurement error.

Currently, statistical inference that is commonly applied to analyse epidemiological studies only accounts for sampling uncertainty. Neglecting model and measurement uncertainty can lead to an underestimation of uncertainty and overconfidence in results, and therefore to contradictory findings when studying the same association of interest in different epidemiological studies. To improve the replicability and credibility of epidemiological findings, it is therefore vital to either pre-emptively reduce these sources of uncertainty during the planning of epidemiological studies, to integrate them when deriving statistical results, or to systematically report their consequences on parameter estimation. Although there are a number of methods to account for model and measurement uncertainty in epidemiological studies, including Bayesian model averaging,^19^ multimodel inference,^20^ simulation extrapolation, regression calibration^21^ and Bayesian hierarchical approaches,^22^ these methods are only rarely applied in practice. In accordance with recent work,^12^^,^^15^ we found that the presence of measurement error in adjustment variables can lead both to bias towards the null and an inflation of effect estimates, underlining the importance of simultaneously accounting for measurement error in the variable of interest and all adjustment variables in a common framework in future studies. To our knowledge, there are currently no methods which can simultaneously account for measurement error in the variable of interest and all adjustment variables when information from a validation sample is lacking, although this would be, in principle, possible in a Bayesian hierarchical framework. In cases where we can neither reduce nor integrate model and measurement uncertainty when deriving statistical results, it is important to study the robustness of results by systematically assessing the impact of these types of uncertainty on parameter estimation.

Some caveats need to be discussed regarding our vibration of effects approach. Firstly, there may be a lack of consensus among experts about which variables can legitimately be considered adjustment variables in a model, and which combinations of adjustments are acceptable and most plausible. The plausible set may be a reduced subset of the full set of all theoretical combinations. However, even experts will often have difficulties agreeing which variables are indispensable. Empirical studies suggest that most observational studies do not include the majority of those variables for which there is a theoretical consensus that they should be considered as adjustment variables.^23^ Other empirical work shows that, even within the same publication, estimates of reported associations for the same exposure–outcome pair under different analyses and models can yield large differences in effect estimates.^24^ Therefore, we argue that considering a substantial number of variables and all their combinations is a legitimate exercise. Secondly, data on the extent of measurement error for exposures, outcomes and adjustment variables may be missing entirely, or existing data from other datasets may not be representative of the respective measurement errors in a new dataset. In the absence of a validation study, investigators should meticulously record what is known and what is unknown about these measurement errors and, in particular, examine the transferability of the magnitude of measurement errors between different studies. Using the proposed vibration of effects framework will allow them to show what influence different sizes of measurement error could have on the stability of the results.

Acknowledging these caveats, the vibration of effects approach provides a flexible tool to systematically assess and compare sampling, model and measurement uncertainty in a common framework. Finally, encouraging the wider use of the vibration of effects concept for understanding model, sampling and measurement uncertainty may further sensitize researchers to the need to think more carefully about these sources of instability. For example, studies rarely report the extent of measurement error for the exposures of interest and do not make a systematic effort to summarise the existing evidence about these measurement errors. It is possible that, in many studies, such evidence does not even exist. Similarly, consideration of confounding and choice of adjustment variables is often sketchy and not well-documented.^25^^,^^26^ In the current illustrative simulations we used a broad range of possible error, but in specific future studies investigators may be able to have a better sense, even at the design phase, of what magnitude of errors need to be anticipated. Moreover, the set of candidate adjustment variables would best be pre-emptively defined. Regardless, the vibration of effects estimations may help place the instability or robustness of study results into better context.

## Supplementary data

Supplementary data are available at *IJE* online.

## Funding

This work was supported by the Deutsche Forschungsgemeinschaft [grant number BO3139/4-3] and the German Federal Ministry of Education and Research [grant number 01IS18036A].

## Supplementary Material

dyaa164_Supplementary_Data
