# Supplementary material for: Examining the robustness of observational associations to model, measurement and sampling uncertainty with the vibration of effects framework
Source: Int J Epidemiol. 2020 Nov 5;50(1):266–78. doi: 10.1093/ije/dyaa164 (PMC7938511; doi:10.1093/ije/dyaa164)
Supplement: dyaa164_Supplementary_Data [file dyaa164_supplementary_data.zip › ije-2020-02-0147-File002.pdf]

Supporting information for the article:

Examining the robustness of observational associations to model, measurement and sampling uncertainty with the vibration of effects framework

Simon Klau*†[1,2], Sabine Hoffmann†[1,3], Chirag J. Patel[4], John P.A. Ioannidis[5,6,7,8,9], and Anne-Laure Boulesteix[1,3]

[1]Institute for Medical Information Processing, Biometry, and Epidemiology, Ludwig-Maximilians-Universität München, Munich, Germany
[2]Leibniz Institute for Prevention Research and Epidemiology – BIPS, Bremen, Germany
[3]LMU Open Science Center, Ludwig-Maximilians-Universität München, Munich, Germany
[4]Department of Biomedical Informatics, Harvard Medical School, Boston, MA, USA
[5]Meta-Research Innovation Center at Stanford (METRICS), Stanford University, Stanford, CA, USA
[6]Department of Epidemiology and Population Health, Stanford University School of Medicine, Stanford, CA, USA
[7]Department of Biomedical Data Science, Stanford University School of Medicine, Stanford, CA, USA
[8]Department of Statistics, Stanford University School of Humanities and Sciences, Stanford, CA, USA
[9]Department of Medicine, Stanford University School of Medicine, Stanford, CA, USA

Measurement error for continuous variables

In section 2.2 of our paper, we introduced ρ_{XZ} as a known correlation between true exposure X and observed exposure Z. In other words, ρ_{XZ} describes the correlation between a measure and a gold standard, where the latter is known to be the truth (Bennett et al., 2017). We claimed that the measurement variance $\text{Var}(U)$ can be obtained by

$$\text{Var}(U) = \frac{\text{Var}(X)}{\rho_{XZ}^2} - \text{Var}(X). \tag{1}$$

With known ρ_{XZ}, this can be easily shown:

*Corresponding author: e-mail: simon.klau@yahoo.de, Department of Medical Information Processing, Biometry and Epidemiology, Ludwig-Maximilians-Universität München, Marchioninistr. 15, D-81377, Munich, Germany

†These authors contributed equally to this work.

$$
\begin{aligned}
\rho_{XZ} &= \mathrm{cor}(Z, X) \\
\rho_{XZ}^2 &= \frac{(\mathrm{Cov}(Z,X))^2}{\mathrm{Var}(Z)\mathrm{Var}(X)} \\
&= \frac{(\mathrm{Var}(X))^2}{\mathrm{Var}(Z)\mathrm{Var}(X)} \\
&= \frac{\mathrm{Var}(X)}{\mathrm{Var}(Z)} \\
&= \frac{\mathrm{Var}(X)}{\mathrm{Var}(X) + \mathrm{Var}(U)} \\
\rho_{XZ}^2(\mathrm{Var}(X) + \mathrm{Var}(U)) &= \mathrm{Var}(X) \\
\rho_{XZ}^2\mathrm{Var}(X) + \rho_{XZ}^2\mathrm{Var}(U) &= \mathrm{Var}(X) \\
\rho_{XZ}^2\mathrm{Var}(U) &= \mathrm{Var}(X) - \rho_{XZ}^2\mathrm{Var}(X) \\
\mathrm{Var}(U) &= \frac{\mathrm{Var}(X)}{\rho_{XZ}^2} - \mathrm{Var}(X).
\end{aligned}
$$

On the other hand, there are test-retest correlations, where two measurements are compared without one that is clearly known to outperform the other (Kimberlin and Winterstein, 2008; Keszei et al., 2010). Here, we do not distinguish further between repeated measurements from one instrument and those from different instruments. Formally, we assume the situation

$$
\begin{aligned}
Z_1 &= X + U_1, \\
Z_2 &= X + U_2, \\
U_1, U_2 &\overset{iid}{\sim} N(0, \mathrm{Var}(U)),
\end{aligned}
$$

where Z_1 and Z_2 are the observed exposures measured by different instruments 1 and 2, X is the true exposure, and U_1 and U_2 are the corresponding measurement errors caused by instruments 1 and 2.

With a known correlation between Z_1 and Z_2, $\rho_{Z_1Z_2}$, we can show that this correlation can be considered instead of ρ_{XZ}^2 in (1), in cases where no gold standard is available:

$$
\begin{aligned}
\rho_{Z_1Z_2}^2 &= \frac{(\mathrm{Cov}(Z_1, Z_2))^2}{\mathrm{Var}(Z_1)\mathrm{Var}(Z_2)} \\
&= \frac{(\mathrm{Cov}(X + U_1, X + U_2))^2}{\mathrm{Var}(X + U_1)\mathrm{Var}(X + U_2)} \\
&= \frac{(\mathrm{Var}(X))^2}{(\mathrm{Var}(X) + \mathrm{Var}(U))^2} \\
\rho_{Z_1Z_2} &= \frac{\mathrm{Var}(X)}{\mathrm{Var}(X) + \mathrm{Var}(U)} \\
\rho_{Z_1Z_2}\mathrm{Var}(X) + \rho_{Z_1Z_2}\mathrm{Var}(U) &= \mathrm{Var}(X) \\
\mathrm{Var}(U) &= \frac{\mathrm{Var}(X) - \rho_{Z_1Z_2}\mathrm{Var}(X)}{\rho_{Z_1Z_2}} \\
\mathrm{Var}(U) &= \frac{\mathrm{Var}(X)}{\rho_{Z_1Z_2}} - \mathrm{Var}(X).
\end{aligned}
$$

Strategy to add measurement error to ordinal variables

In section 2.2 of our paper, we described our strategy to add measurement error to continuous and binary variables. For ordinal variables, we assume latent variables which follow a normal distribution. As a first step, our strategy consists in defining feasible cutpoints that reflect the characteristics of the variables. For example, the NHANES data comprises the five-categorical variable BMI which can simply be defined by assuming a continuous variable with the cutpoints of 18.5, 25, 30 and 35, as these are the values the five categories are actually based on. For physical activity, the cutpoints are based on Health.gov physical activity guideline categories. For the variable income, we searched for tertiles of US household income values on the internet. Finally, for education, we assume the number of years in school to be an underlying continuous variable and choose the cutpoints according to the level of education. For more information on the coding of the categorical variables, we refer to Patel et al. (2015). Given the relative number of observations for each category, optimal parameters of the normal distribution can be obtained by minimizing the squared distance between original and estimated cutpoints. For each category, the cutpoints can be used to draw observations from a truncated normal distribution with the parameters that were obtained in the previous step. After adding measurement error as described for the continuous variables, the cutpoints can again be used to discretize the variable with measurement error.

Overview of all variables used in our study

Table S1: Variables from the NHANES and their purpose in our study

Name	Variable of interest	Adjustment
diabetes	✓	✓
heart disease	✓	✓
thigh circumference	✓	
HDL-cholesterol	✓	
hypertension	✓	✓
any cancer	✓	✓
family history of heart disease	✓	✓
segmented neutrophils number	✓	
maximal calf circumference	✓	
standing height	✓	
waist circumference	✓	
weight	✓	
60 sec. pulse	✓	
number of dietary supplements taken	✓	
hepatitis A antibody	✓	
hepatitis B core antibody	✓	
hepatitis B surface antibody	✓	
lymphocyte percent	✓	
mean platelet volume	✓	
calcium	✓	
sodium	✓	
osmolality	✓	
phosphorus	✓	
sex	✓	✓
pest control	✓	
pneumonia	✓	
private water source	✓	
water treatment	✓	
age	✓	✓
passive smoking	✓	
physical activity		✓
race/ethnicity		✓
active smoking		✓
bmi		✓
total cholesterol		✓
drink five drinks per day (alcohol consumption)		✓
income		✓
education		✓

Literature search for measurement error for variables from the NHANES data

The following tables comprise variables of interest and adjustment variables, except the variables that we assume to be measured without error (age, sex, and race/ethnicity). Furthermore, we are not aware of any studies that provide information on the following variables: pulse measurement, pneumonia, the number of dietary supplements taken in the last 30 days, osmolality, hepatitis A antibody, pest control, private water source and water treatment.

Table S2: Sensitivities and specificities found in the literature in order to generate measurement error for binary variables from the NHANES data

Name	Sensitivity		Specificity		References
	low	high	low	high	
diabetes	0.49	0.80	0.91	1.00	Molenaar et al. (2006)
heart disease	0.88	0.88	0.98	0.98	Barr et al. (2009)
hypertension	0.14	0.71	0.71	0.99	Molenaar et al. (2006)
any cancer	0.33	0.98	0.14	0.96	Rauscher et al. (2008)
family history of heart disease	0.67	0.89	0.59	0.97	Scheuner et al. (2008)
passive smoking	0.38	0.66	0.79	0.97	Arechavala et al. (2018)
active smoking	0.77	1.00	0.79	1.00	Rebagliato (2002)
alcohol consumption	0.38	0.96	0.64	1.00	Allen et al. (1997)
mean	0.56	0.85	0.73	0.98	

Table S3: Correlations found in the literature in order to generate measurement error for continuous variables from the NHANES data. Gold standard correlations were modified in advance such that all values in this table can be considered as test-retest correlations. Values in the upper part of the table refer to situations where a correlation with a gold standard was modified by calculating its squared value. The lower part of the table comprises the test-retest correlations as originally provided in the literature. For waist circumference, weight, and sodium, both test-retest and gold standard correlations were found.

Name	Type	Correlation		References
		low	high	
standing height	numeric	0.59	0.98	Brener et al. (2003)
				Nakamura et al. (1999)
physical activity	ordinal	0.02	0.74	Ferrari et al. (2007)
bmi	ordinal	0.48	0.88	Fonseca et al. (2010)
				Nakamura et al. (1999)
income	ordinal	0.09	0.81	Moore et al. (2000)
waist circumference	numeric	0.61	0.99	Battram et al. (2011)
				Ulijaszek and Kerr (1999)
weight	numeric	0.81	0.93	Brener et al. (2003)
				Nakamura et al. (1999)
sodium	numeric	0.68	0.96	Jain et al. (2009)
				Yildirim et al. (2015)
thigh circumference	numeric	0.98	0.98	Moreno et al. (2003)
HDL-cholesterol	numeric	0.70	0.79	Al-Delaimy et al. (2006)
segmented neutrophils number	numeric	0.95	0.99	Stamminger et al. (1998)
maximal calf circumference	numeric	0.99	0.99	Ulijaszek and Kerr (1999)
hepatitis B surface antibody	numeric	0.74	0.84	Huzly et al. (2008)
hepatitis B core antibody	numeric	0.74	0.84	Huzly et al. (2008)
lymphocyte percent	numeric	0.90	0.98	Stamminger et al. (1998)
				Grimaldi and Scopacasa (2000)
mean platelet volume	numeric	0.66	0.99	Grimaldi and Scopacasa (2000)
				Tóth et al. (2006)
calcium	numeric	0.30	0.69	Buscemi et al. (2015)
				Satia-Abouta et al. (2003)
phosphorus	numeric	0.36	0.54	Buscemi et al. (2015)
total cholesterol	numeric	0.63	0.77	Al-Delaimy et al. (2006)
education	ordinal	0.99	0.99	Maisto et al. (1982)
mean		0.73	0.90	

Concept of our simulation study

In section 2.4, we briefly introduced the concept of our simulation study with the aim of comparing measurement, sampling and model vibration for sample sizes that can both be smaller and larger than the initial sample size of the NHANES data. Here, we provide some more details. In this simulation study, we generate data with sample sizes $n \in \{500, 1000, 5000, 10000, 50000, 100000, 200000\}$. The simulated data is based on the real NHANES data in the sense that we adopt the correlation structure as well as the effect sizes of the variables on the real data.

In order to generate the covariates we first use the R-package `GenOrd` (Barbiero and Ferrari, 2015) for the generation of covariates of binary and ordinal type with an appropriate covariance structure. Subsequently, we generate the continuous variables in an iterative way by modeling them through a linear regression. Then we use the predicted values together with the estimated variance to generate values for the continuous covariate.

In particular, to generate values for the continuous variable age, we use the original data set and model age as an outcome variable in a linear regression. In this linear regression, we use all binary and ordinal variables as covariates. Based on this linear model and the simulated binary and ordinal data obtained by using `GenOrd`, we predict new values for age. Moreover, we add random values from a normal distribution with mean zero and variance which is equal to the estimated variance of the random error to this variable. To generate values for the continuous variable total cholesterol, we use the same procedure, but consider age as additional covariate in the linear regression. In the case where we have a continuous variable of interest, we model this variable with age and total cholesterol as additional covariates.

Based on these covariate values, we generate exponentially distributed survival times (Bender et al., 2005) as a function of the variable of interest and all 15 potential adjustment variables. Moreover, we use the effect sizes of the variables, which we obtained from a model fit based on the real data set, and determine λ_t as 1 divided by the mean of the original event times. Finally, we generate exponentially distributed censoring times with λ_c as 1 divided by the mean of the original censoring times. Due to a sometimes unrealistically large number of events, we multiply λ_c successively with an increasing factor to ensure event rates in an interval between 0.2 and 0.3. For more detailed information, we refer to our R code, which is also provided as Supplementary Material for the original article.

Bibliography

Al-Delaimy, W. K., Jansen, E. H. J. M., Peeters, P. H. M., van der Laan, J. D., van Noord, P. A. H., Boshuizen, H. C., van der Schouw, Y. T., Jenab, M., Ferrari, P., and Bueno-de Mesquita, H. B. (2006). Reliability of biomarkers of iron status, blood lipids, oxidative stress, vitamin d, c-reactive protein and fructosamine in two dutch cohorts. *Biomarkers*, 11(4):370–382.

Allen, J. P., Litten, R. Z., Fertig, J. B., and Babor, T. (1997). A review of research on the alcohol use disorders identification test (AUDIT). *Alcoholism: Clinical and Experimental Research*, 21(4):613–619.

Arechavala, T., Continente, X., Pérez-Ríos, M., Fernández, E., Cortés-Francisco, N., Schiaffino, A., Centrich, F., Muñoz, G., and López, M. J. (2018). Validity of self-reported indicators to assess secondhand smoke exposure in the home. *Environmental Research*, 164:340–345.

Barbiero, A. and Ferrari, P. A. (2015). *GenOrd: Simulation of Discrete Random Variables with Given Correlation Matrix and Marginal Distributions*. R package version 1.4.0.

Barr, E. L. M., Tonkin, A. M., Welborn, T. A., and Shaw, J. E. (2009). Validity of self-reported cardiovascular disease events in comparison to medical record adjudication and a statewide hospital morbidity database: the AusDiab study. *Internal Medicine Journal*, 39(1):49–53.

Battram, D. S., Beynon, C., and He, M. (2011). The reliability and validity of using clothing size as a proxy for waist circumference measurement in adults. *Applied Physiology, Nutrition, and Metabolism*, 36(2):183–190.

Bender, R., Augustin, T., and Blettner, M. (2005). Generating survival times to simulate Cox proportional hazards models. *Statistics in Medicine*, 24(11):1713–1723.

Bennett, D. A., Landry, D., Little, J., and Minelli, C. (2017). Systematic review of statistical approaches to quantify, or correct for, measurement error in a continuous exposure in nutritional epidemiology. *BMC Medical Research Methodology*, 17(146):1–22.

Brener, N. D., McManus, T., Galuska, D. A., Lowry, R., and Wechsler, H. (2003). Reliability and validity of self-reported height and weight among high school students. *Journal of Adolescent Health*, 32(4):281–287.

Buscemi, S., Rosafio, G., Vasto, S., Massenti, F. M., Grosso, G., Galvano, F., Rini, N., Barile, A. M., Maniaci, V., Cosentino, L., and Verga, S. (2015). Validation of a food frequency questionnaire for use in Italian adults living in Sicily. *International Journal of Food Sciences and Nutrition*, 66(4):426–438.

Ferrari, P., Friedenreich, C., and Matthews, C. E. (2007). The role of measurement error in estimating levels of physical activity. *American Journal of Epidemiology*, 166(7):832–840.

Fonseca, H., Silva, A. M., Matos, M. G., Esteves, I., Costa, P., Guerra, A., and Gomes-Pedro, J. (2010). Validity of BMI based on self-reported weight and height in adolescents. *Acta Paediatrica*, 99(1):83–88.

Grimaldi, E. and Scopacasa, F. (2000). Evaluation of the Abbott CELL-DYN 4000 hematology analyzer. *American Journal of Clinical Pathology*, 113(4):497–505.

Huzly, D., Schenk, T., Jilg, W., and Neumann-Haefelin, D. (2008). Comparison of nine commercially available assays for quantification of antibody response to hepatitis B virus surface antigen. *Journal of Clinical Microbiology*, 46(4):1298–1306.

Jain, A., Subhan, I., and Joshi, M. (2009). Comparison of the point-of-care blood gas analyzer versus the laboratory auto-analyzer for the measurement of electrolytes. *International Journal of Emergency Medicine*, 2(2):117–120.

Keszei, A. P., Novak, M., and Streiner, D. L. (2010). Introduction to health measurement scales. *Journal of Psychosomatic Research*, 68(4):319–323.

Kimberlin, C. L. and Winterstein, A. G. (2008). Validity and reliability of measurement instruments used in research. *American Journal of Health-System Pharmacy*, 65(23):2276–2284.

Maisto, S. A., Sobell, L. C., Cooper, A. M., and Sobell, M. B. (1982). Comparison of two techniques to obtain retrospective reports of drinking behavior from alcohol abusers. *Addictive Behaviors*, 7(1):33–38.

Molenaar, E. A., van Ameijden, E. J. C., Grobbee, D. E., and Numans, M. E. (2006). Comparison of routine care self-reported and biometrical data on hypertension and diabetes: results of the Utrecht Health Project. *European Journal of Public Health*, 17(2):199–205.

Moore, J. C., Stinson, L. L., and Welniak, E. J. (2000). Income measurement error in surveys: a review. *Journal of Official Statistics*, 16(4):331–361.

Moreno, L. A., Joyanes, M., Mesana, M. I., González-Gross, M., Gil, C. M., Sarría, A., Gutierrez, A., Garaulet, M., Perez-Prieto, R., Bueno, M., Marcos, A., et al. (2003). Harmonization of anthropometric measurements for a multicenter nutrition survey in Spanish adolescents. *Nutrition*, 19(6):481–486.

Nakamura, K., Hoshino, Y., Kodama, K., and Yamamoto, M. (1999). Reliability of self-reported body height and weight of adult Japanese women. *Journal of Biosocial Science*, 31(4):555–558.

Patel, C. J., Burford, B., and Ioannidis, J. P. A. (2015). Assessment of vibration of effects due to model specification can demonstrate the instability of observational associations. *J Clin Epidemiol*, 68(9):1046–58.

Rauscher, G. H., Johnson, T. P., Cho, Y. I., and Walk, J. A. (2008). Accuracy of self-reported cancer-screening histories: a meta-analysis. *Cancer Epidemiology, Biomarkers & Prevention*, 17(4):748–757.

Rebagliato, M. (2002). Validation of self reported smoking. *Journal of Epidemiology & Community Health*, 56(3):163–164.

Satia-Abouta, J., Patterson, R. E., King, I. B., Stratton, K. L., Shattuck, A. L., Kristal, A. R., Potter, J. D., Thornquist, M. D., and White, E. (2003). Reliability and validity of self-report of vitamin and mineral supplement use in the vitamins and lifestyle study. *American Journal of Epidemiology*, 157(10):944–954.

Scheuner, M. T., Setodji, C. M., Pankow, J. S., Blumenthal, R. S., and Keeler, E. (2008). Relation of familial patterns of coronary heart disease, stroke, and diabetes to subclinical atherosclerosis: the multi-ethnic study of atherosclerosis. *Genetics in Medicine*, 10(12):879–887.

Stamminger, G., Köppel, C., Schaub, A., Gärtner, I., Tonndorf, C., Meyer, K., Elix, P., Lang, B., and Beier, L. (1998). Performance of the SE-9000 automated haematology analyser in a laboratory serving a haematological oncology unit. *Clinical & Laboratory Haematology*, 20(3):143–149.

Tóth, O., Calatzis, A., Penz, S., Losonczy, H., and Siess, W. (2006). Multiple electrode aggregometry: a new device to measure platelet aggregation in whole blood. *Thrombosis and Haemostasis*, 96(6):781–788.

Ulijaszek, S. J. and Kerr, D. A. (1999). Anthropometric measurement error and the assessment of nutritional status. *British Journal of Nutrition*, 82(3):165–177.

Yildirim, E., Karapinar, T., and Hayirli, A. (2015). Reliability of the i-STAT for the determination of blood electrolyte (K+, Na+, and CI-) concentrations in cattle. *Journal of Veterinary Internal Medicine*, 29(1):388–394.

Supplementary Figures

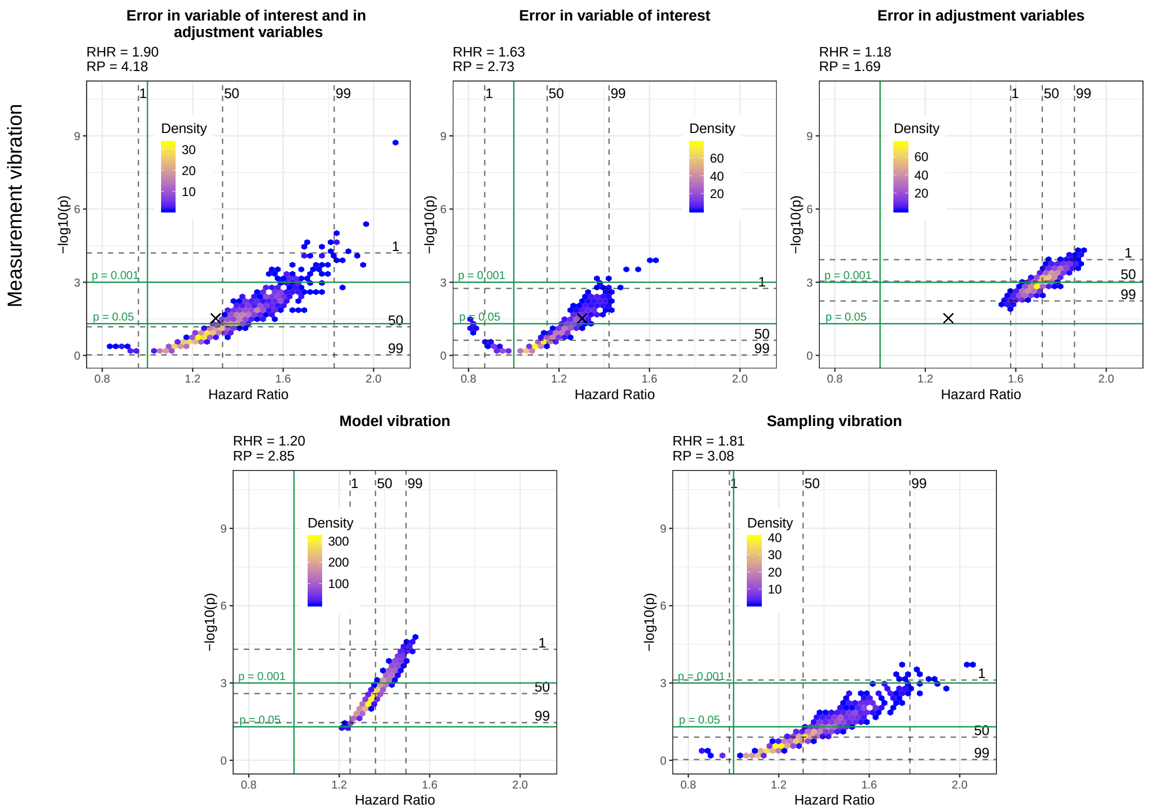

Figure S1: Volcano plots for different types of vibration and different scenarios of measurement vibration when **hypertension** is the variable of interest. The summary measures RHR and RP indicate relative hazard ratios and relative p-values, respectively. The black cross in the top panel indicates the model without measurement error.

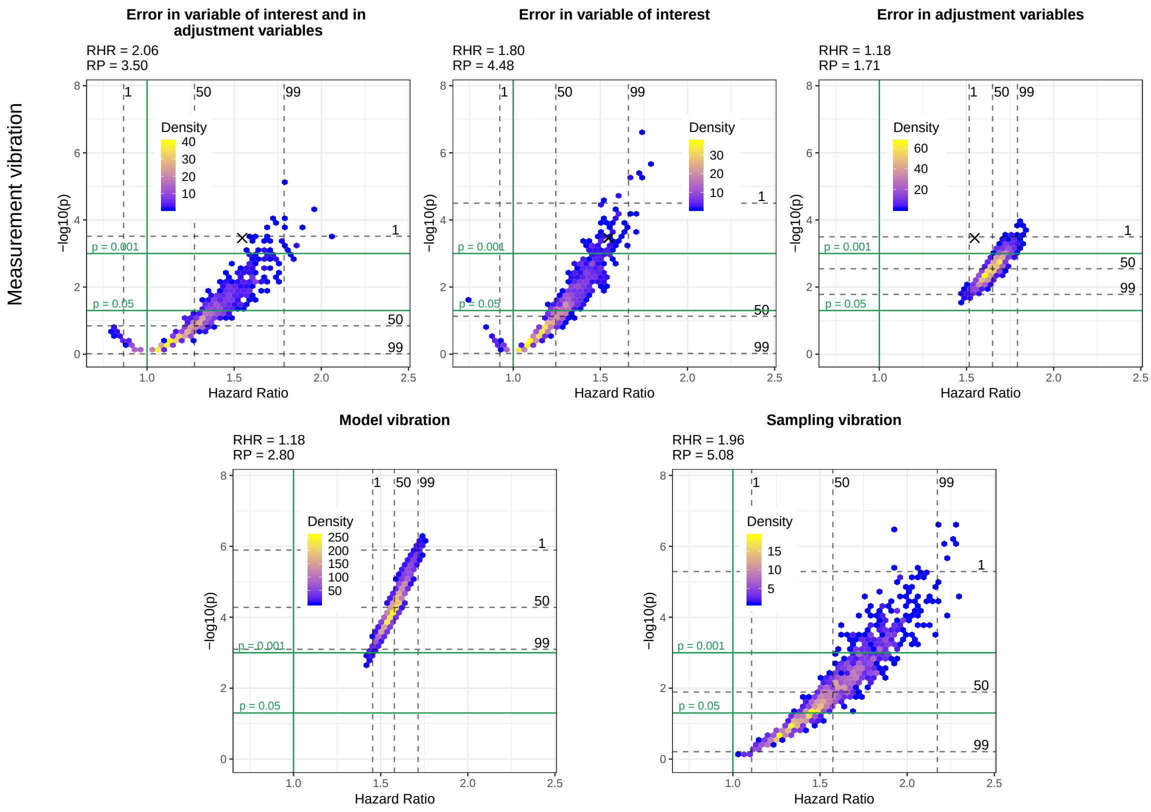

Figure S2: Volcano plots for different types of vibration and different scenarios of measurement vibration when **any cancer** is the variable of interest. The summary measures RHR and RP indicate relative hazard ratios and relative p-values, respectively. The black cross in the top panel indicates the model without measurement error.

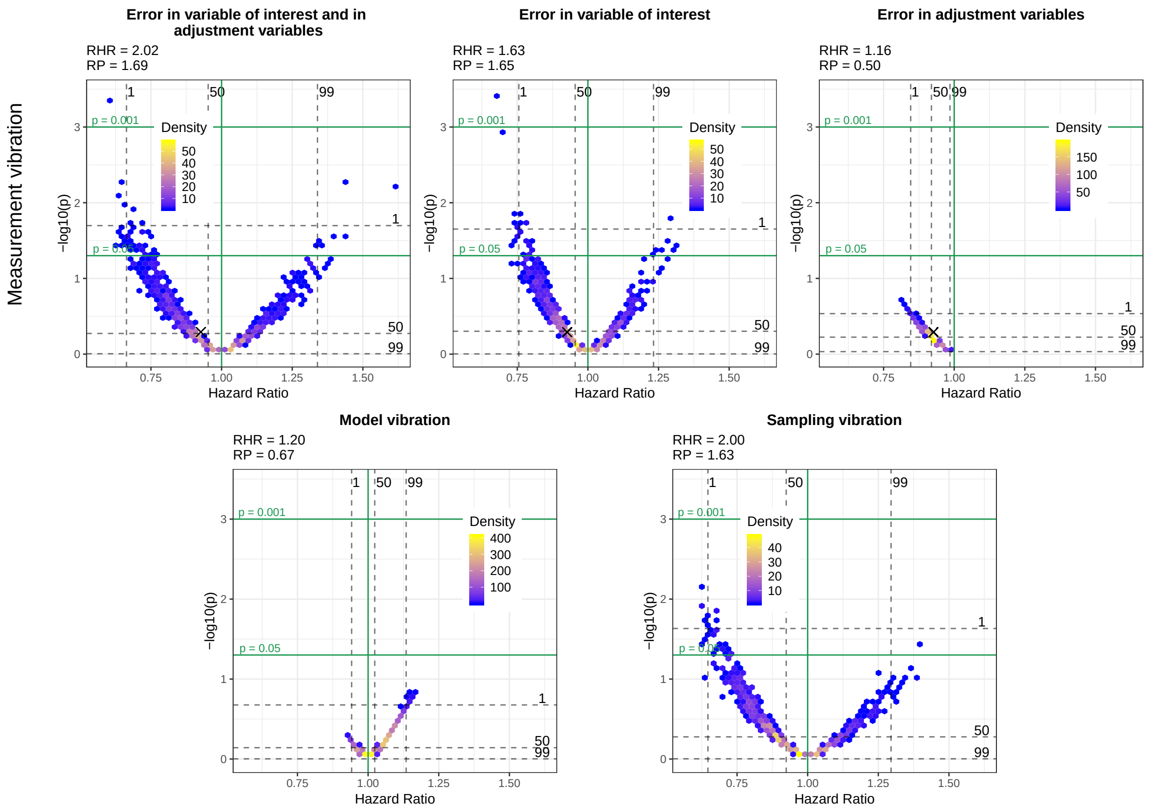

Figure S3: Volcano plots for different types of vibration and different scenarios of measurement vibration when **family history of heart disease** is the variable of interest. The summary measures RHR and RP indicate relative hazard ratios and relative p-values, respectively. The black cross in the top panel indicates the model without measurement error.

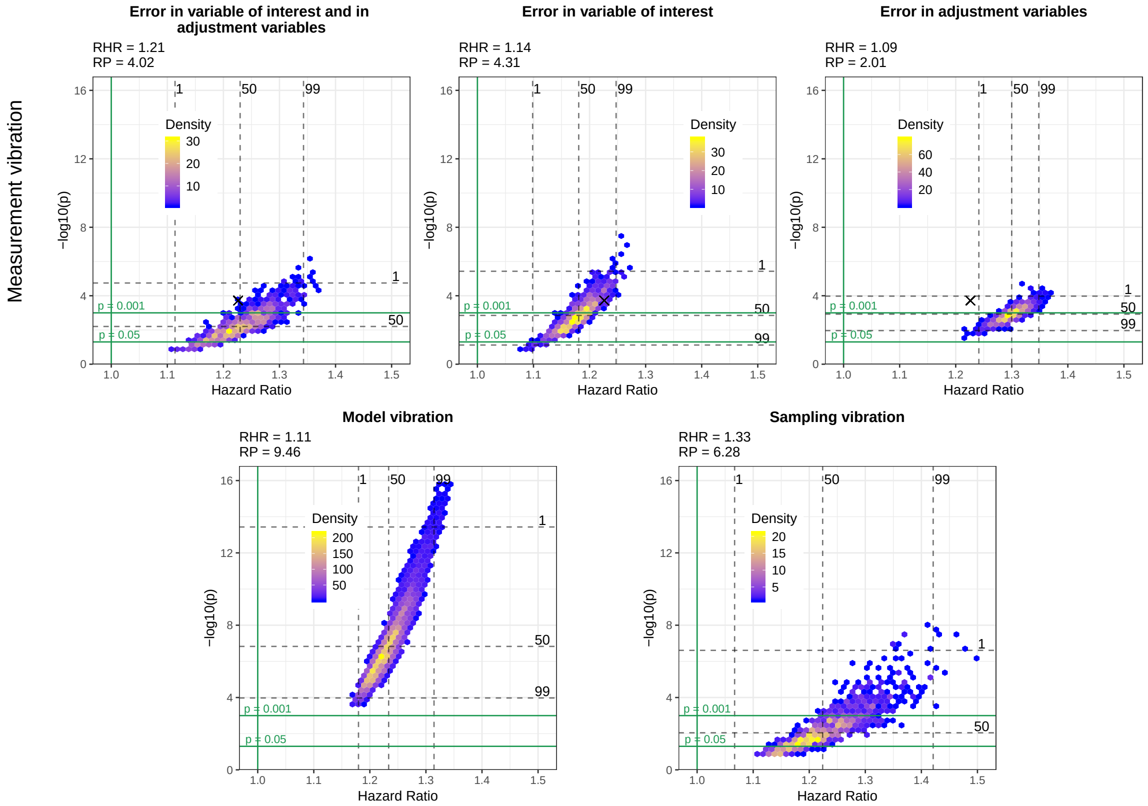

Figure S4: Volcano plots for different types of vibration and different scenarios of measurement vibration when **segmented neutrophils number** is the variable of interest. The summary measures RHR and RP indicate relative hazard ratios and relative p-values, respectively. The black cross in the top panel indicates the model without measurement error.

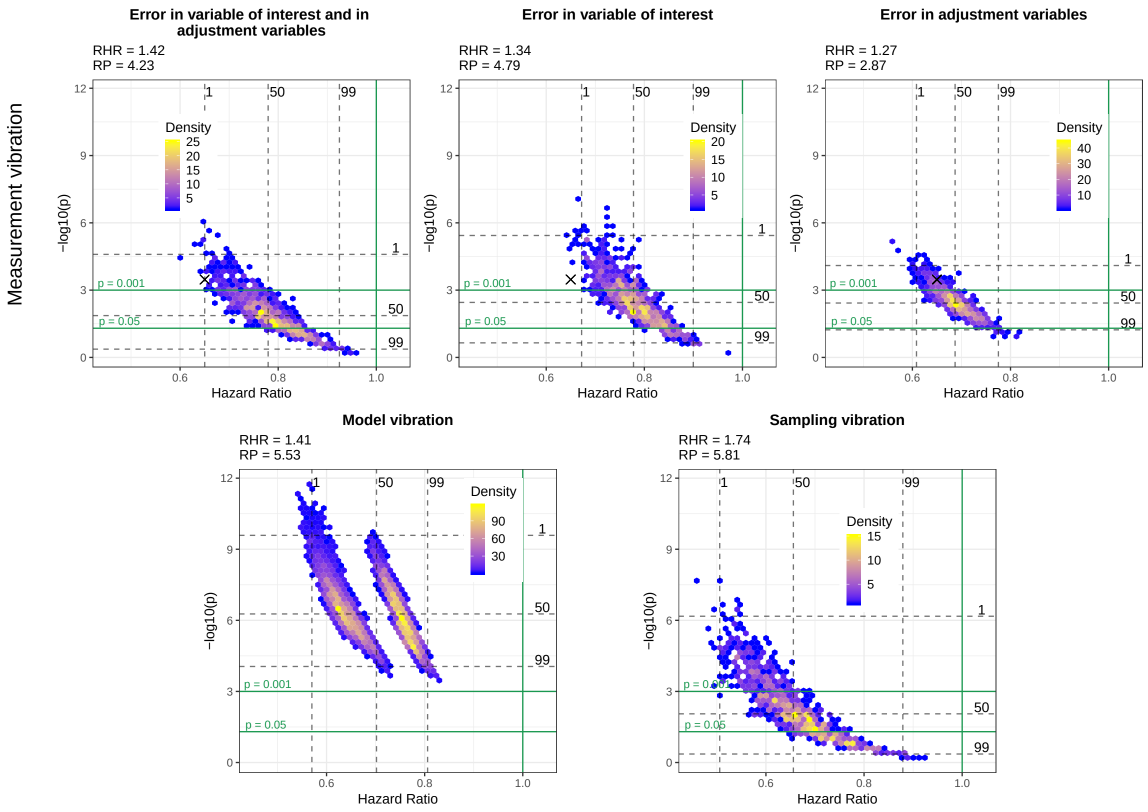

Figure S5: Volcano plots for different types of vibration and different scenarios of measurement vibration when **maximal calf circumference** is the variable of interest. The summary measures RHR and RP indicate relative hazard ratios and relative p-values, respectively. The black cross in the top panel indicates the model without measurement error.

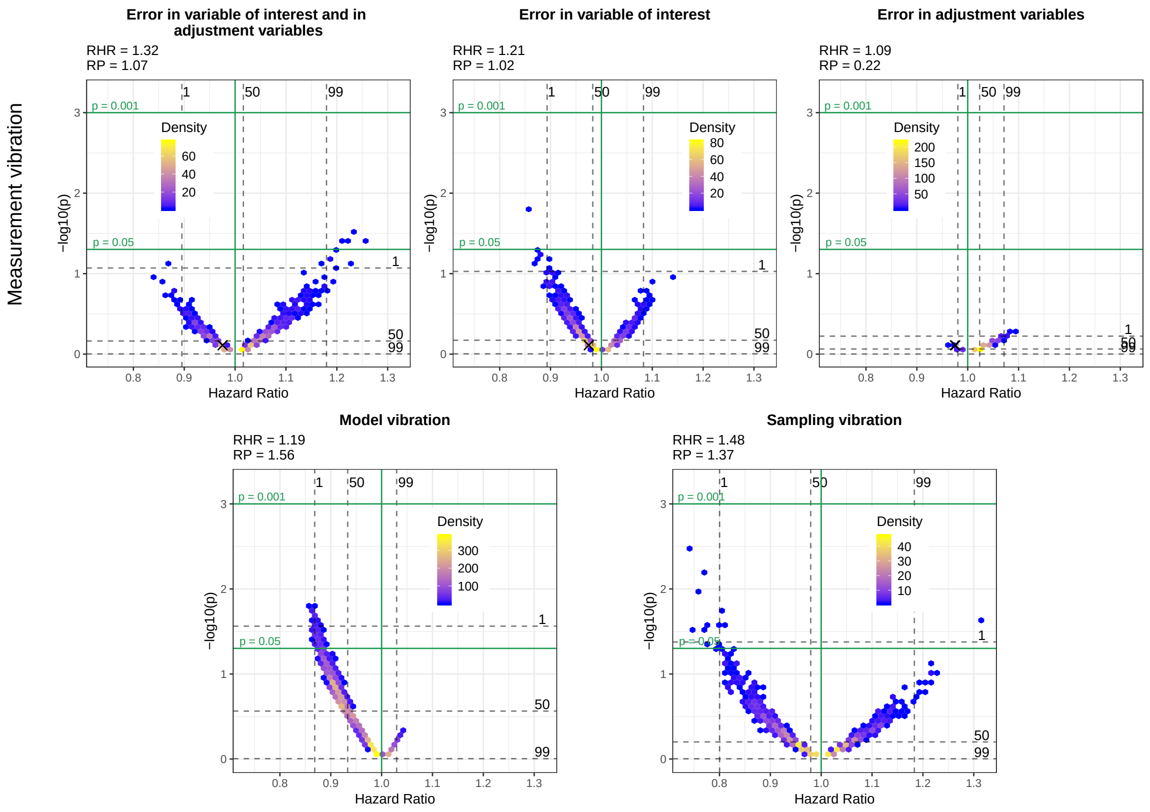

Figure S6: Volcano plots for different types of vibration and different scenarios of measurement vibration when **standing height** is the variable of interest. The summary measures RHR and RP indicate relative hazard ratios and relative p-values, respectively. The black cross in the top panel indicates the model without measurement error.

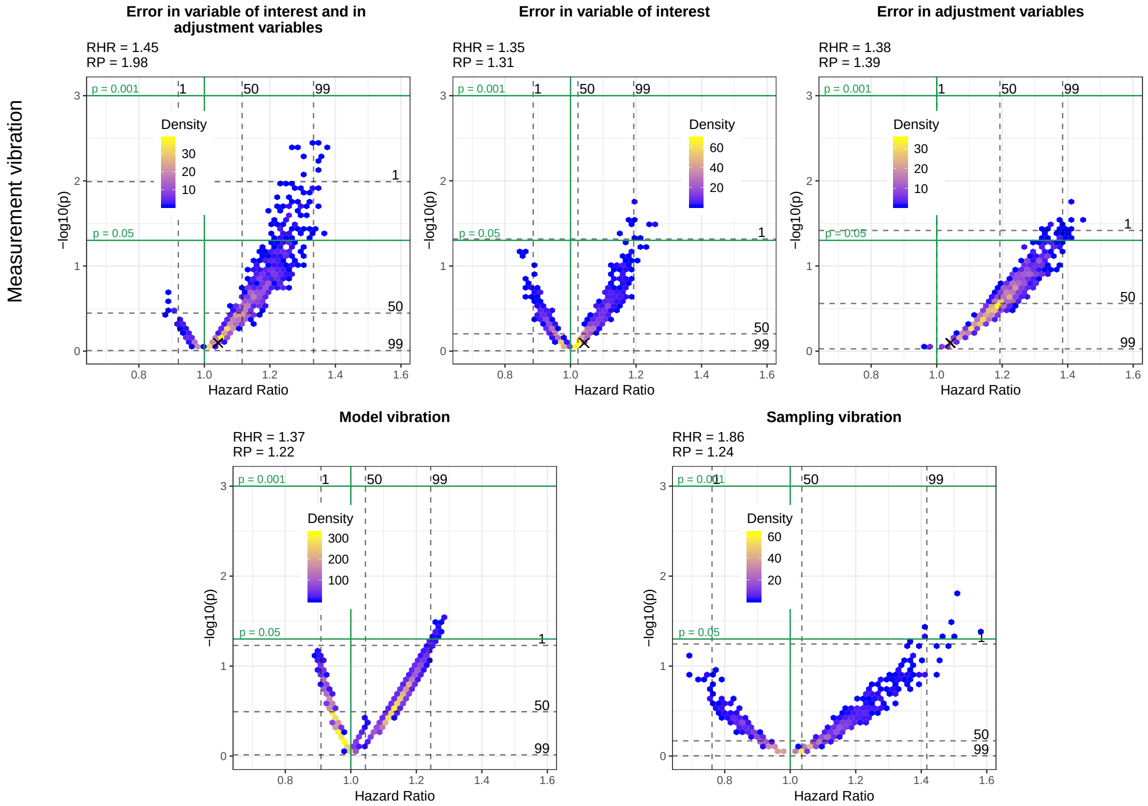

Figure S7: Volcano plots for different types of vibration and different scenarios of measurement vibration when **waist circumference** is the variable of interest. The summary measures RHR and RP indicate relative hazard ratios and relative p-values, respectively. The black cross in the top panel indicates the model without measurement error.

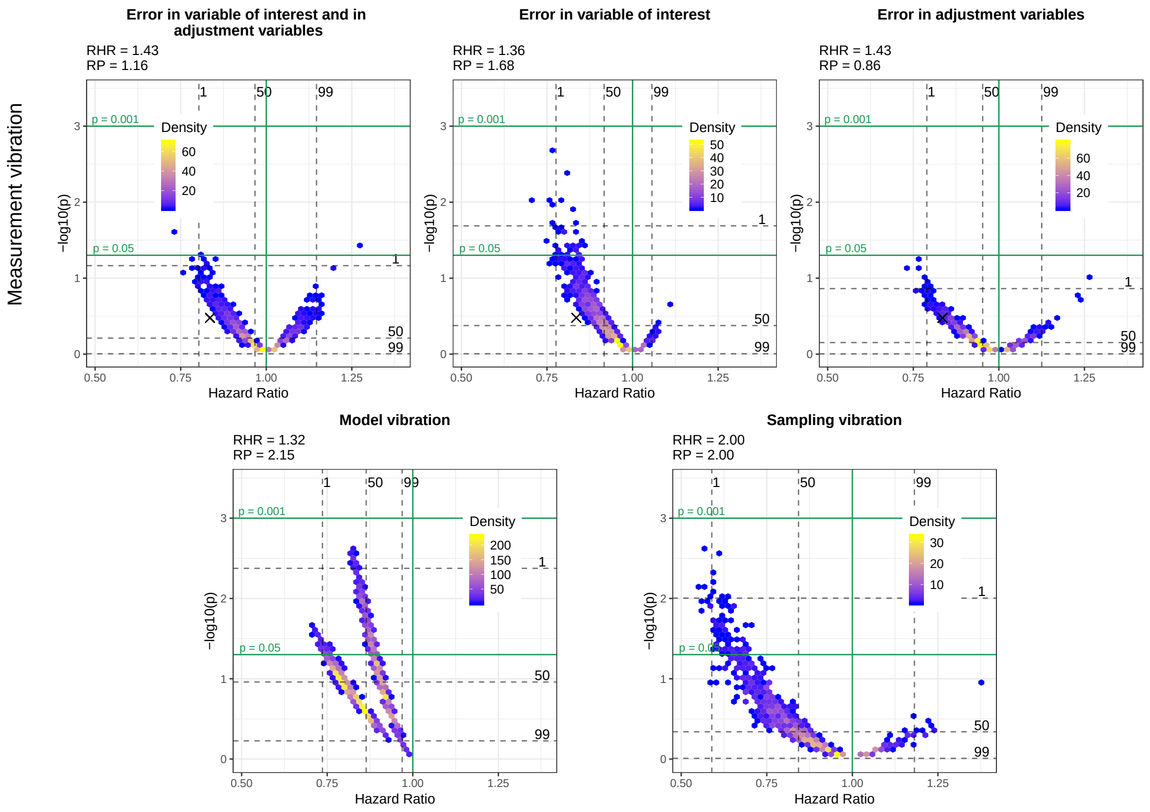

Figure S8: Volcano plots for different types of vibration and different scenarios of measurement vibration when **weight** is the variable of interest. The summary measures RHR and RP indicate relative hazard ratios and relative p-values, respectively. The black cross in the top panel indicates the model without measurement error.

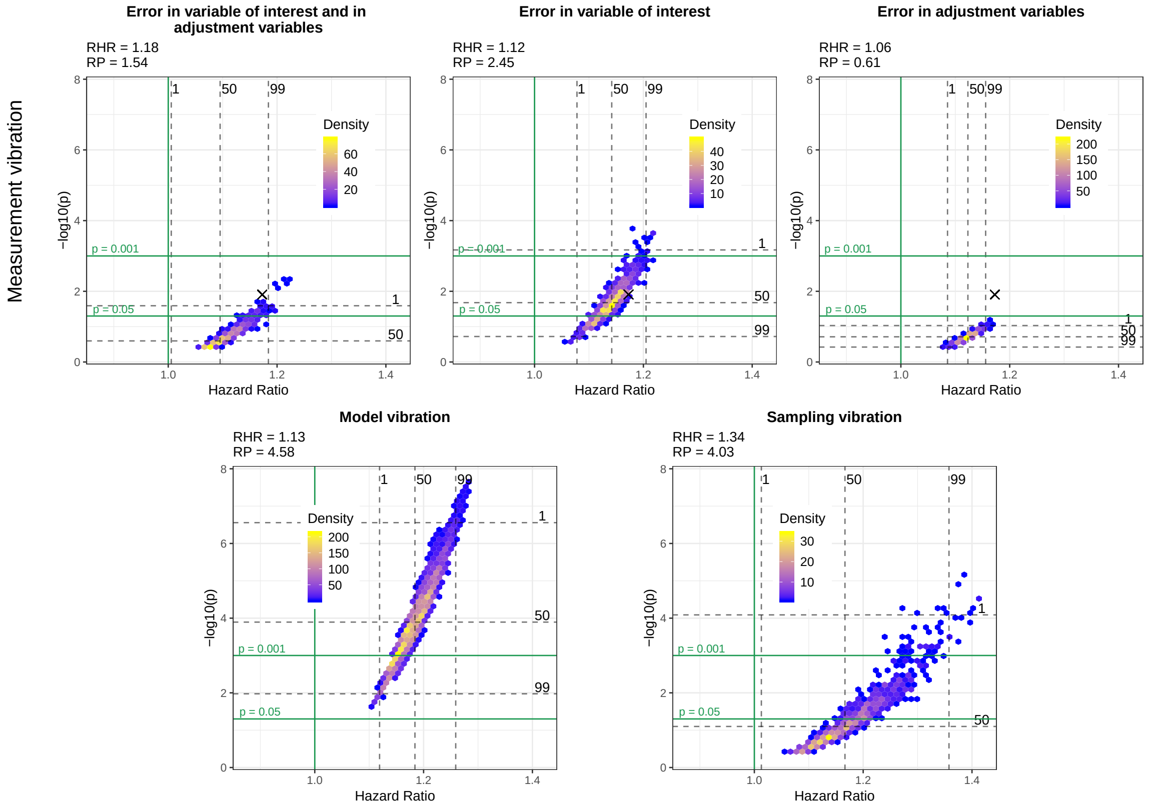

Figure S9: Volcano plots for different types of vibration and different scenarios of measurement vibration when **60 sec. pulse** is the variable of interest. The summary measures RHR and RP indicate relative hazard ratios and relative p-values, respectively. The black cross in the top panel indicates the model without measurement error.

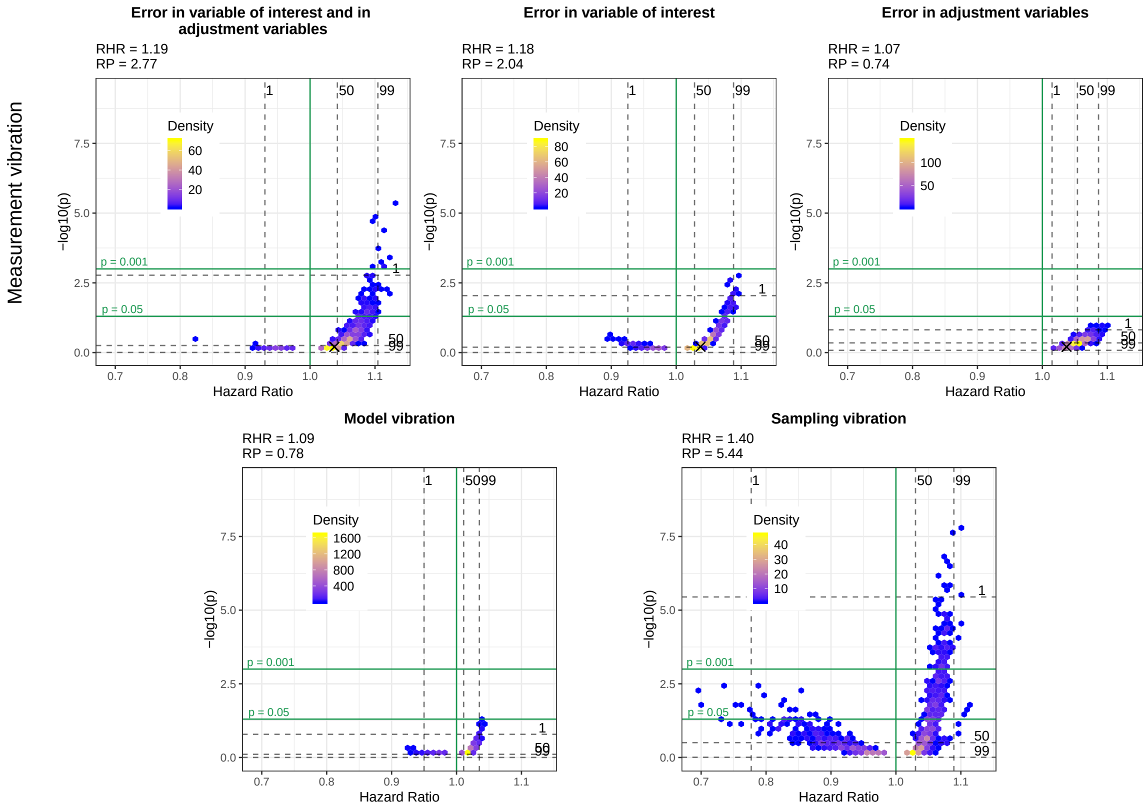

Figure S10: Volcano plots for different types of vibration and different scenarios of measurement vibration when **number of dietary supplements taken** is the variable of interest. The summary measures RHR and RP indicate relative hazard ratios and relative p-values, respectively. The black cross in the top panel indicates the model without measurement error.

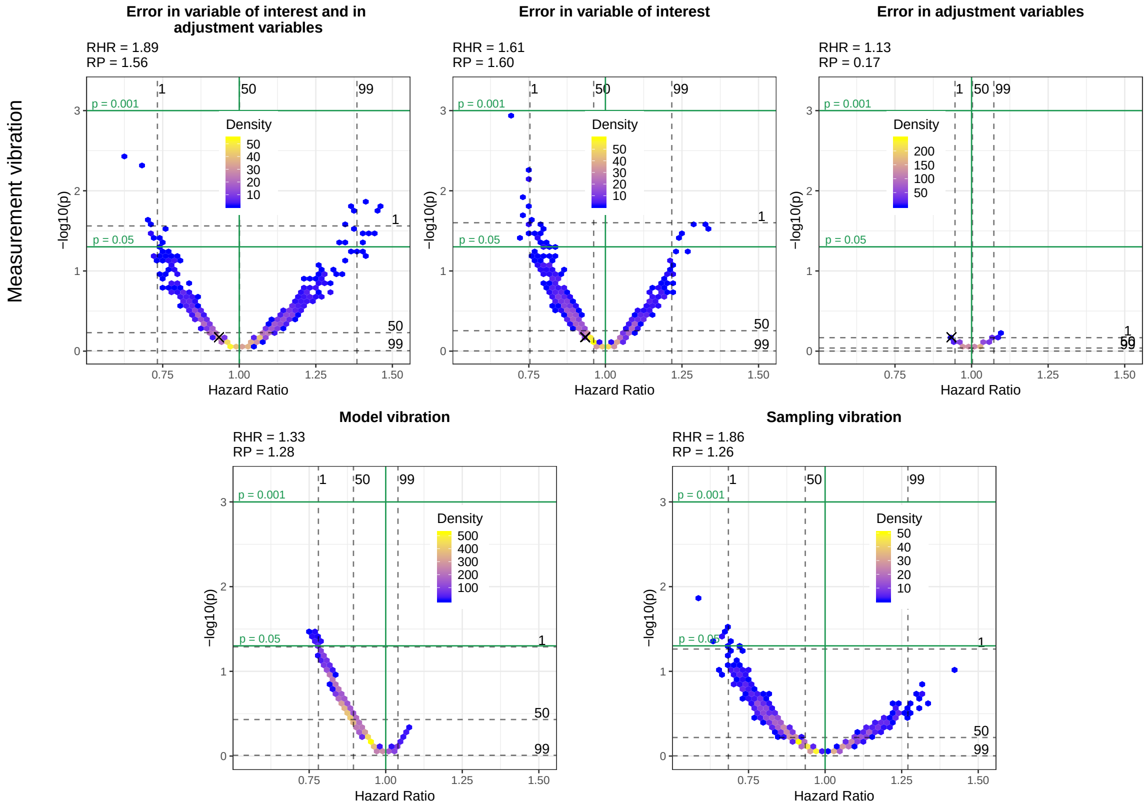

Figure S11: Volcano plots for different types of vibration and different scenarios of measurement vibration when **hepatitis A antibody** is the variable of interest. The summary measures RHR and RP indicate relative hazard ratios and relative p-values, respectively. The black cross in the top panel indicates the model without measurement error.

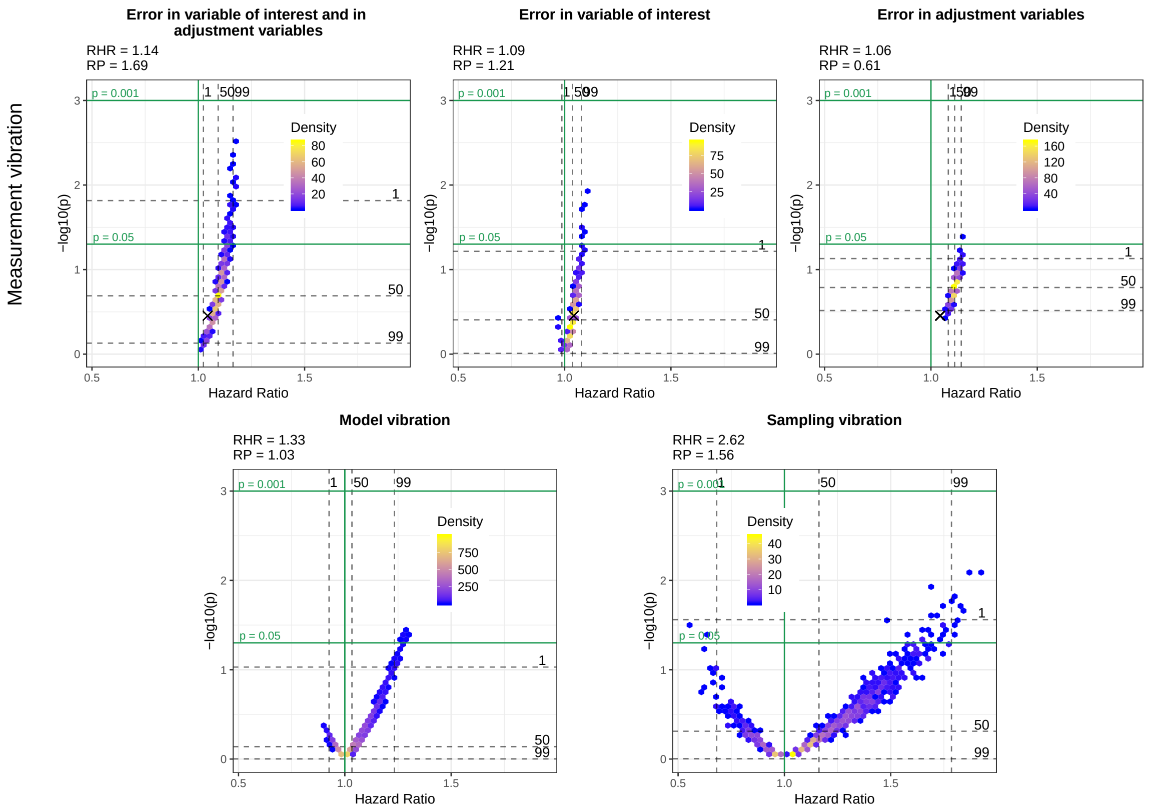

Figure S12: Volcano plots for different types of vibration and different scenarios of measurement vibration when **hepatitis B core antibody** is the variable of interest. The summary measures RHR and RP indicate relative hazard ratios and relative p-values, respectively. The black cross in the top panel indicates the model without measurement error.

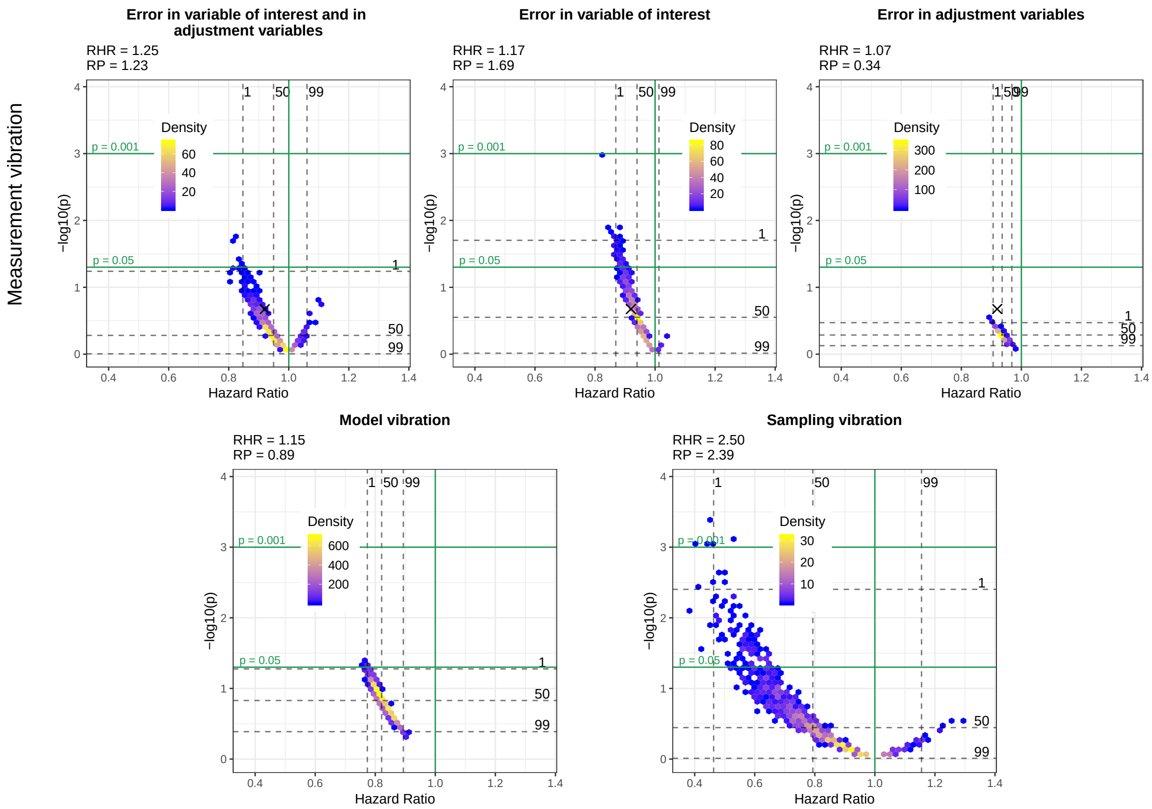

Figure S13: Volcano plots for different types of vibration and different scenarios of measurement vibration when **hepatitis B surface antibody** is the variable of interest. The summary measures RHR and RP indicate relative hazard ratios and relative p-values, respectively. The black cross in the top panel indicates the model without measurement error.

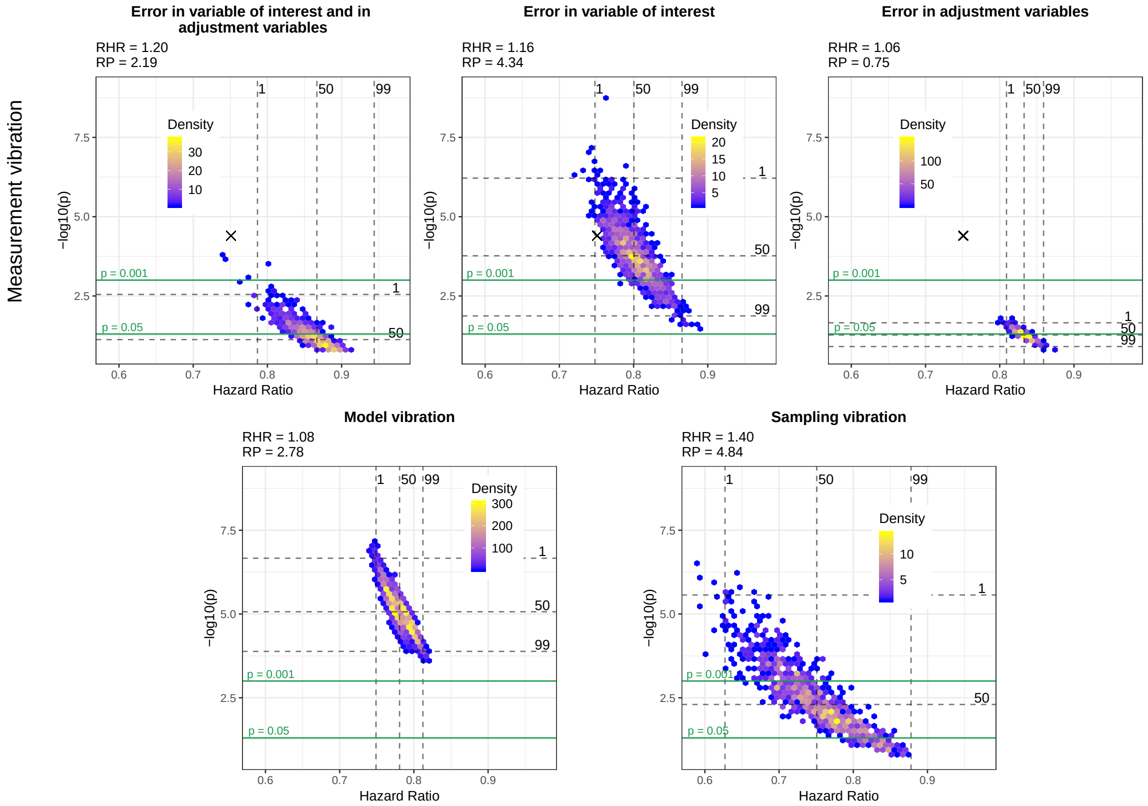

Figure S14: Volcano plots for different types of vibration and different scenarios of measurement vibration when **lymphocyte percent** is the variable of interest. The summary measures RHR and RP indicate relative hazard ratios and relative p-values, respectively. The black cross in the top panel indicates the model without measurement error.

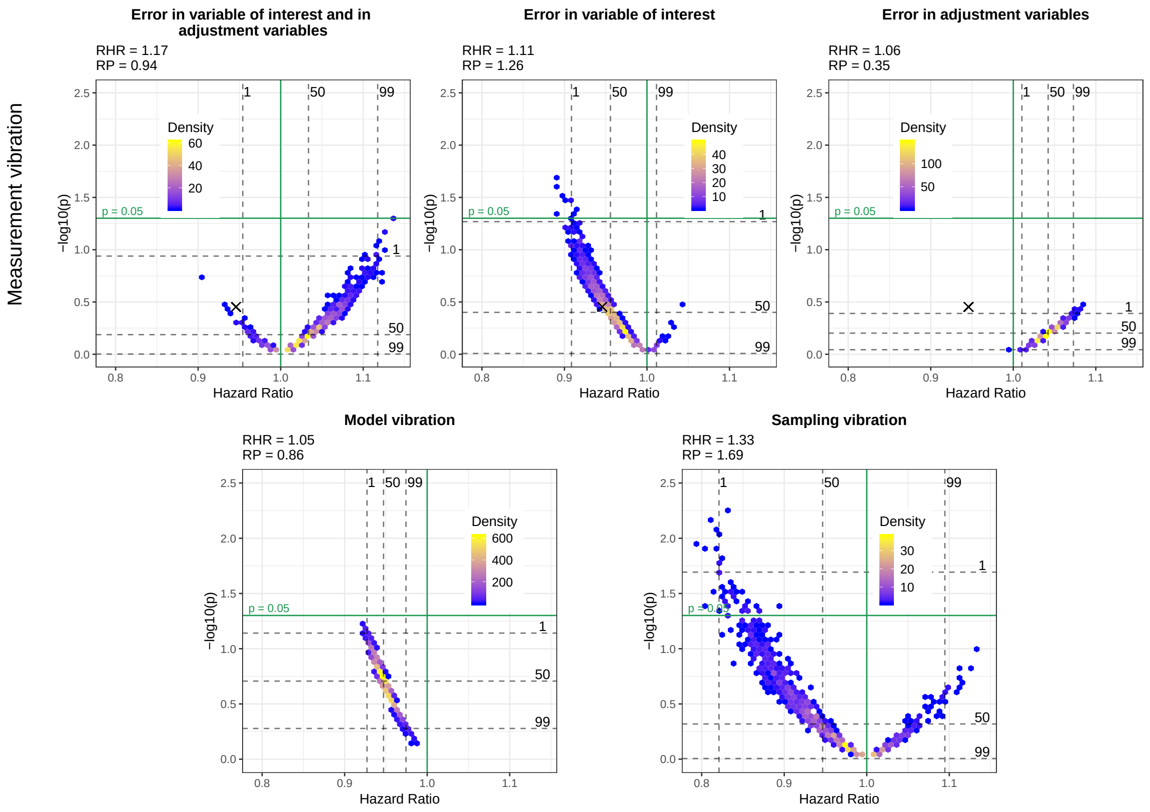

Figure S15: Volcano plots for different types of vibration and different scenarios of measurement vibration when **mean platelet volume** is the variable of interest. The summary measures RHR and RP indicate relative hazard ratios and relative p-values, respectively. The black cross in the top panel indicates the model without measurement error.

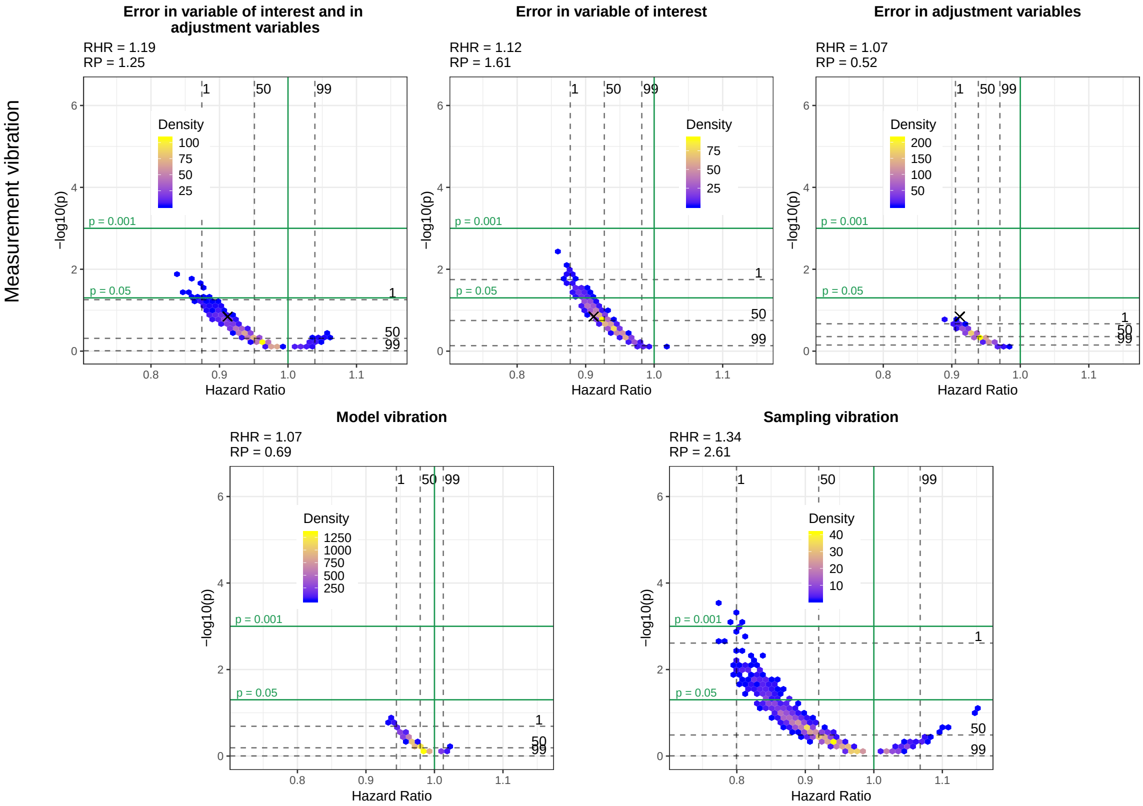

Figure S16: Volcano plots for different types of vibration and different scenarios of measurement vibration when **calcium** is the variable of interest. The summary measures RHR and RP indicate relative hazard ratios and relative p-values, respectively. The black cross in the top panel indicates the model without measurement error.

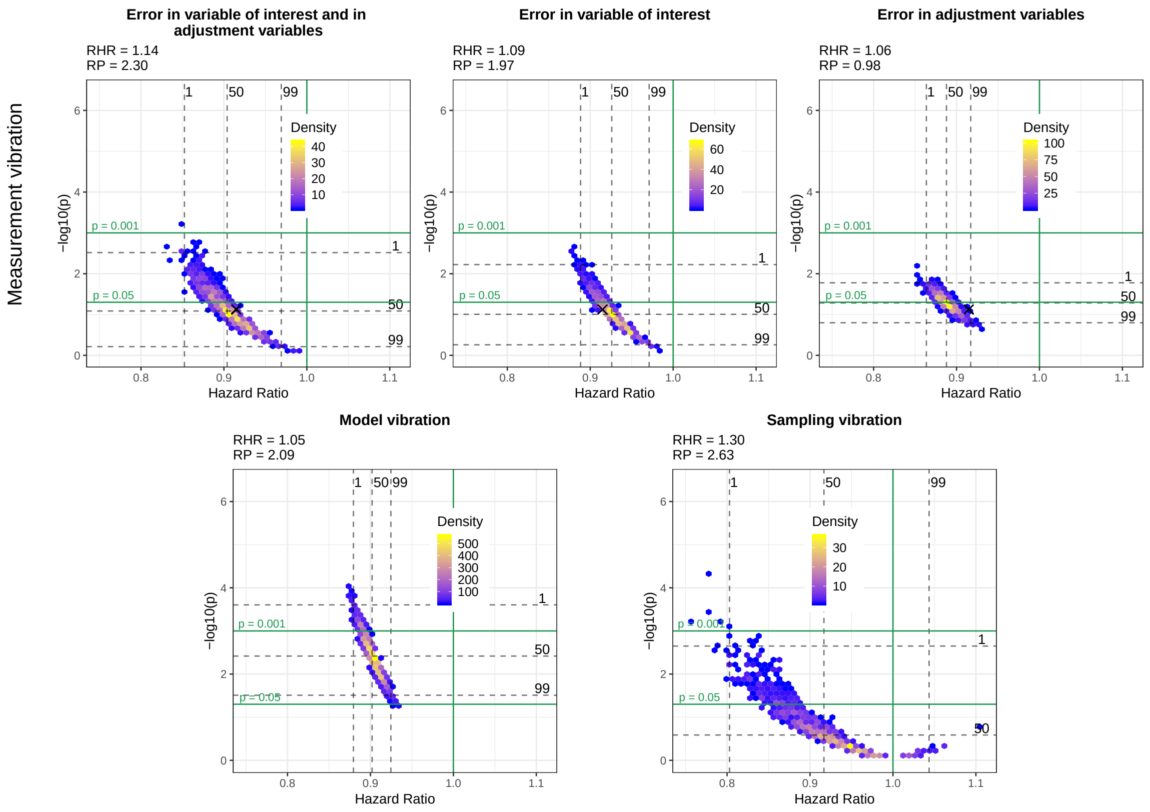

Figure S17: Volcano plots for different types of vibration and different scenarios of measurement vibration when **sodium** is the variable of interest. The summary measures RHR and RP indicate relative hazard ratios and relative p-values, respectively. The black cross in the top panel indicates the model without measurement error.

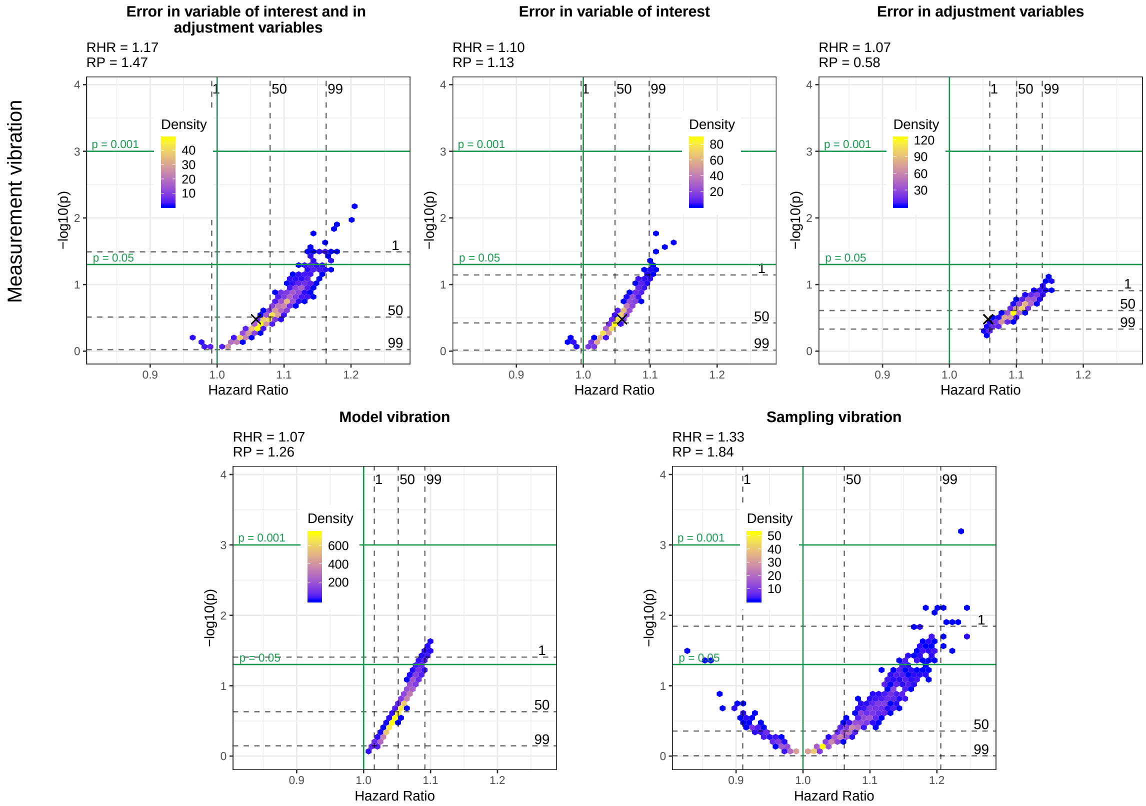

Figure S18: Volcano plots for different types of vibration and different scenarios of measurement vibration when **osmolality** is the variable of interest. The summary measures RHR and RP indicate relative hazard ratios and relative p-values, respectively. The black cross in the top panel indicates the model without measurement error.

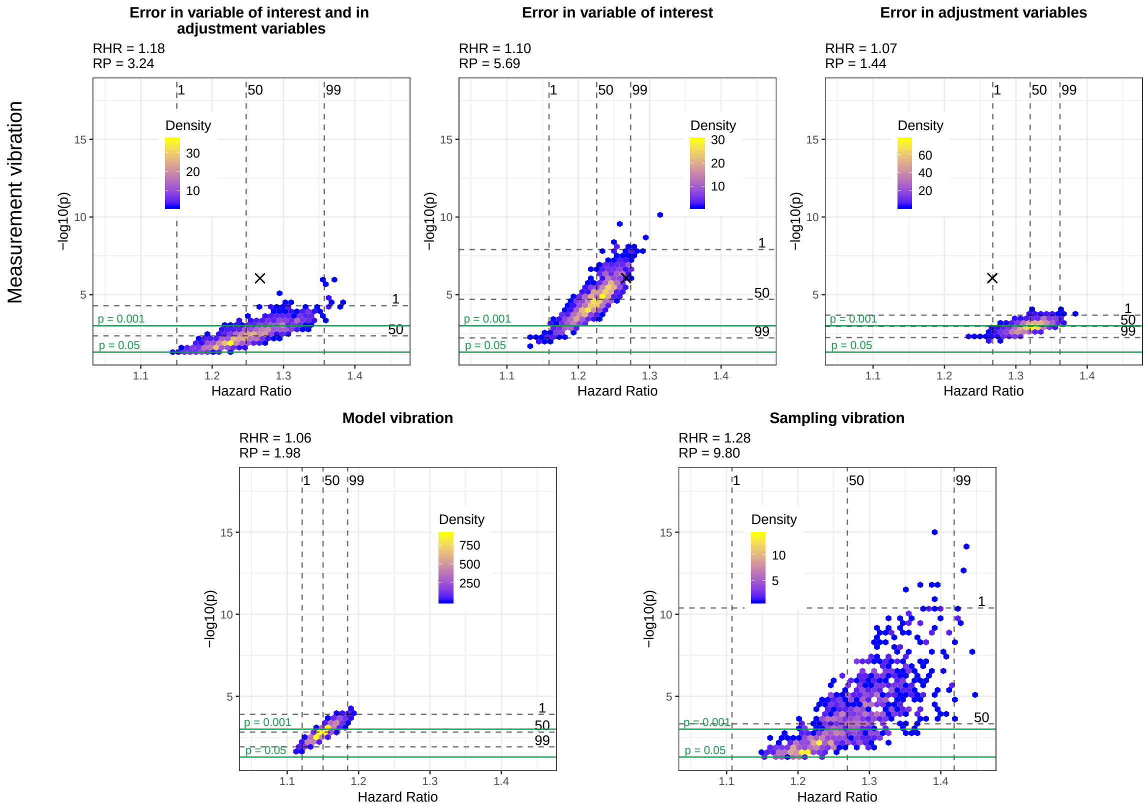

Figure S19: Volcano plots for different types of vibration and different scenarios of measurement vibration when **phosphorus** is the variable of interest. The summary measures RHR and RP indicate relative hazard ratios and relative p-values, respectively. The black cross in the top panel indicates the model without measurement error.

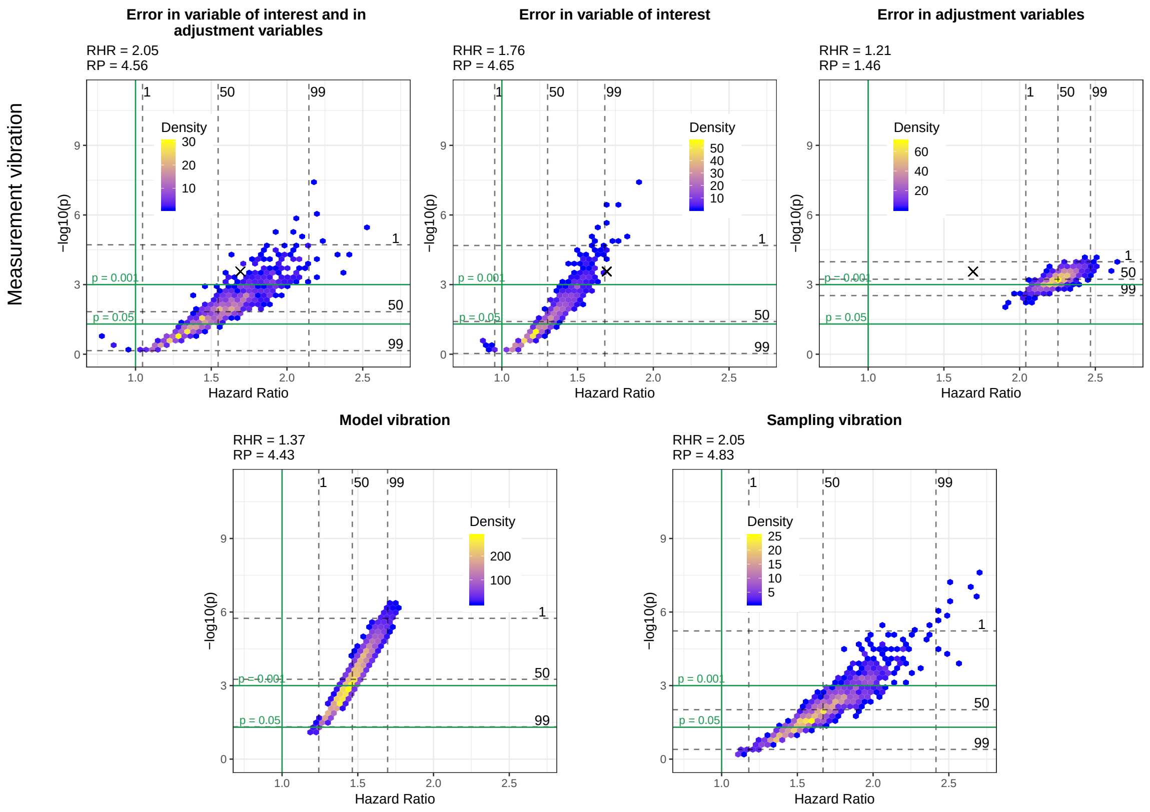

Figure S20: Volcano plots for different types of vibration and different scenarios of measurement vibration when **sex** is the variable of interest. The summary measures RHR and RP indicate relative hazard ratios and relative p-values, respectively. The black cross in the top panel indicates the model without measurement error.

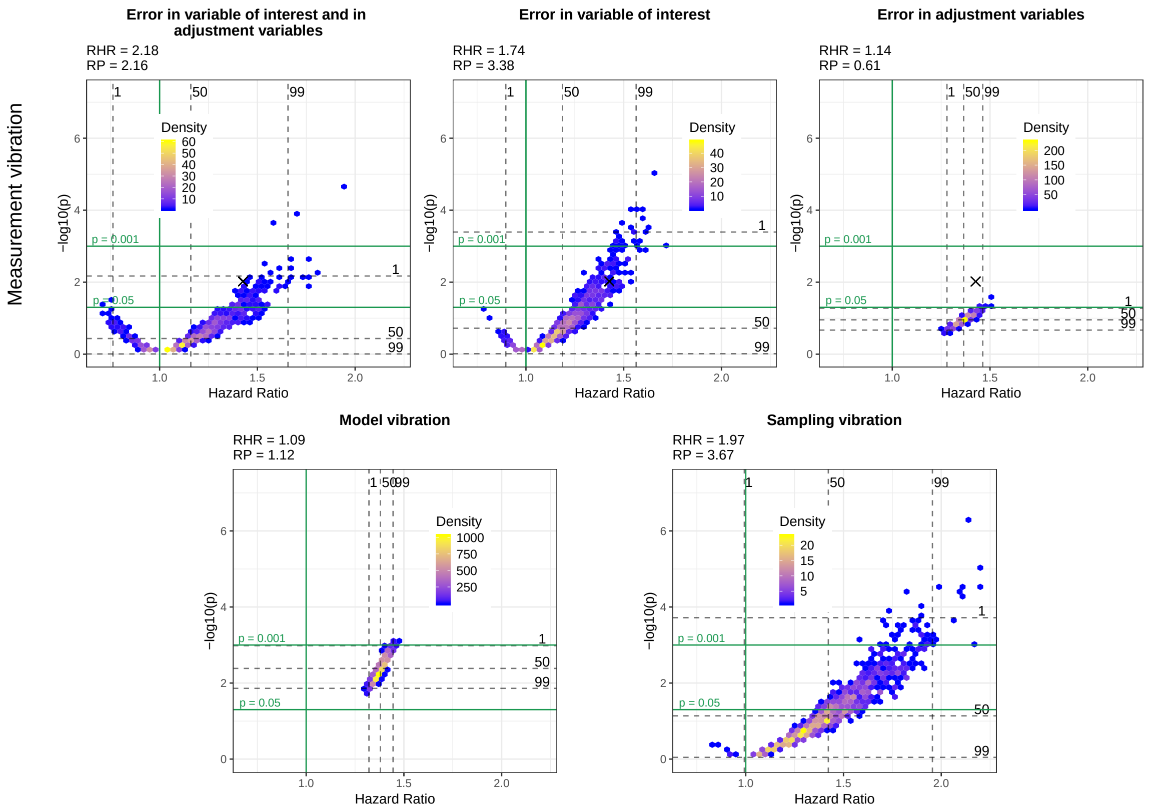

Figure S21: Volcano plots for different types of vibration and different scenarios of measurement vibration when **pest control** is the variable of interest. The summary measures RHR and RP indicate relative hazard ratios and relative p-values, respectively. The black cross in the top panel indicates the model without measurement error.

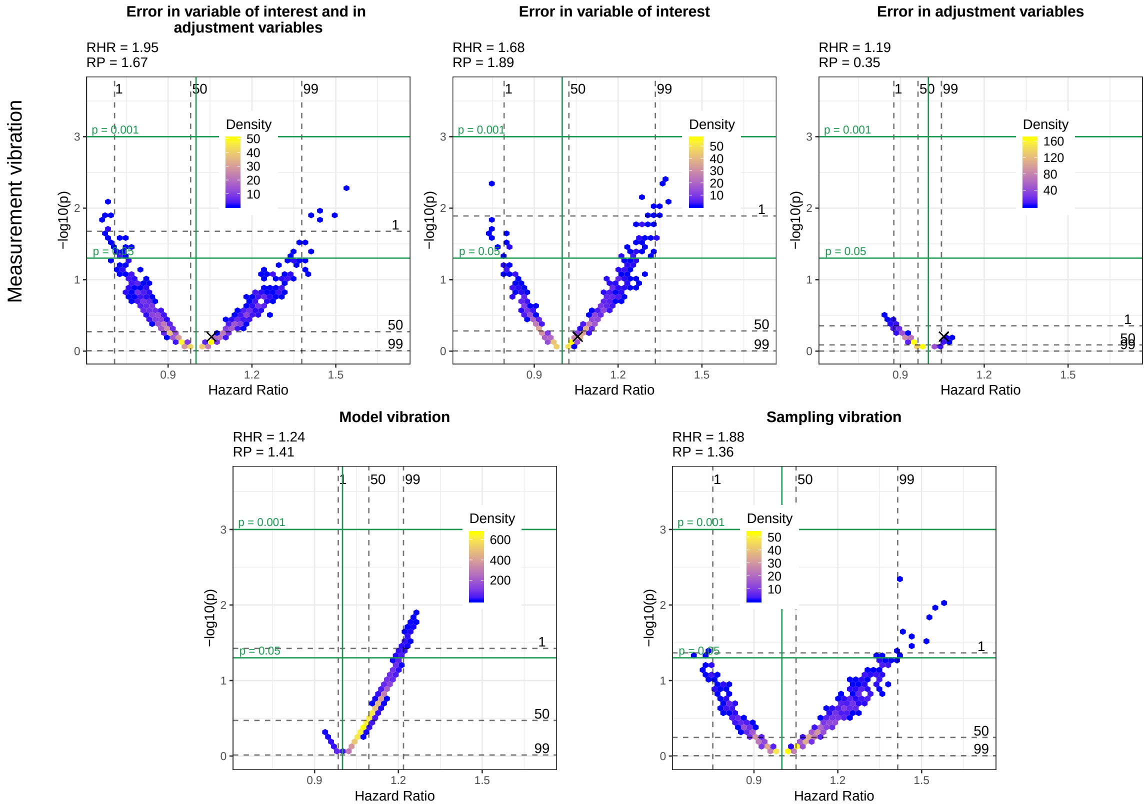

Figure S22: Volcano plots for different types of vibration and different scenarios of measurement vibration when **pneumonia** is the variable of interest. The summary measures RHR and RP indicate relative hazard ratios and relative p-values, respectively. The black cross in the top panel indicates the model without measurement error.

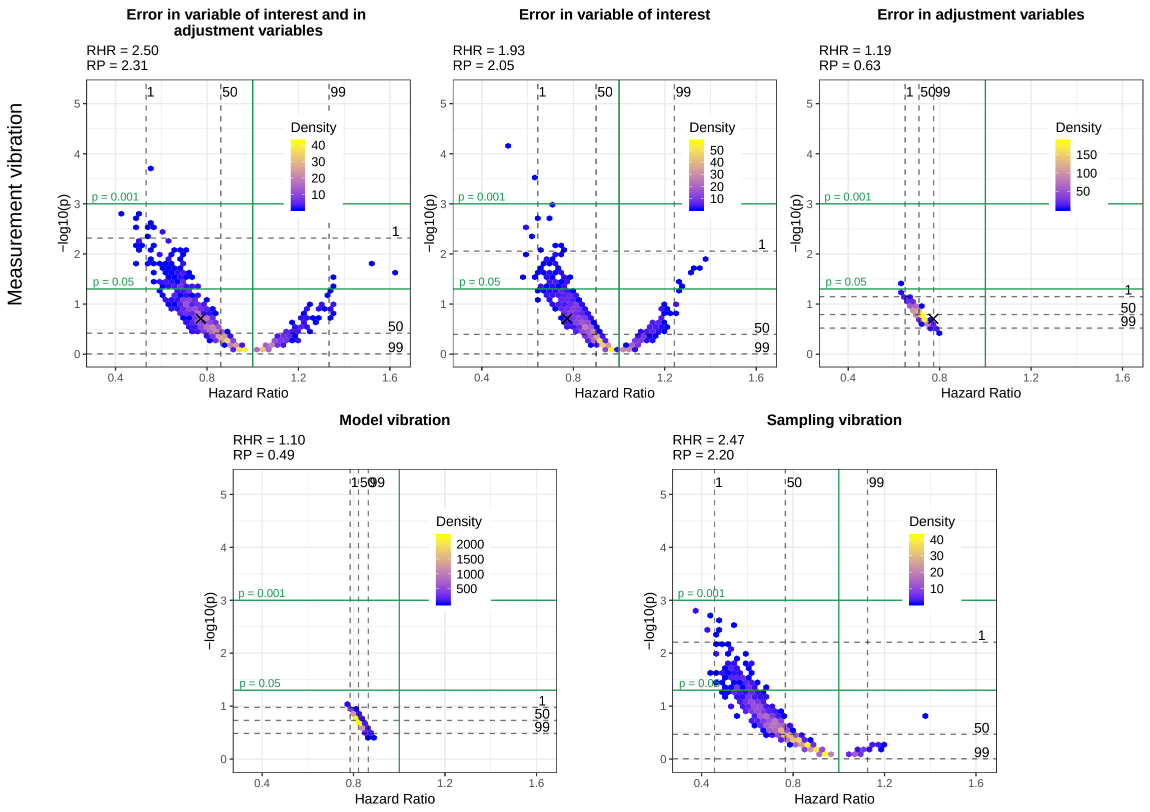

Figure S23: Volcano plots for different types of vibration and different scenarios of measurement vibration when **private water source** is the variable of interest. The summary measures RHR and RP indicate relative hazard ratios and relative p-values, respectively. The black cross in the top panel indicates the model without measurement error.

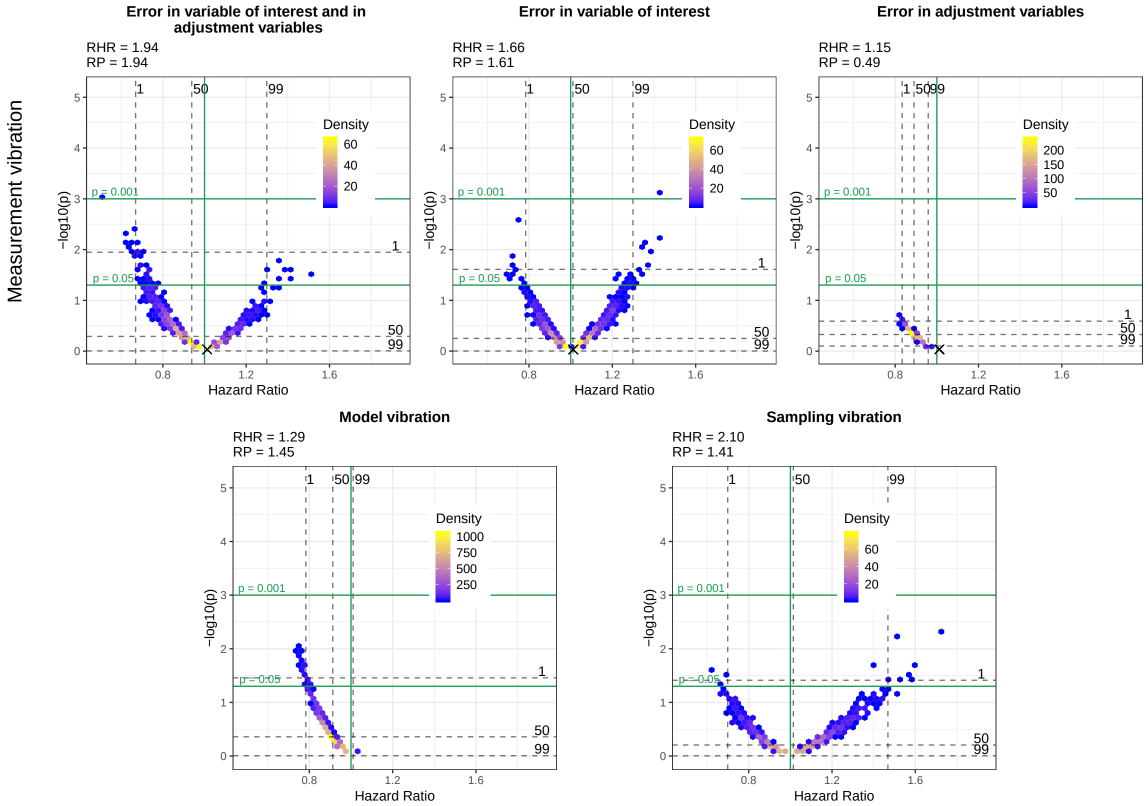

Figure S24: Volcano plots for different types of vibration and different scenarios of measurement vibration when **water treatment** is the variable of interest. The summary measures RHR and RP indicate relative hazard ratios and relative p-values, respectively. The black cross in the top panel indicates the model without measurement error.

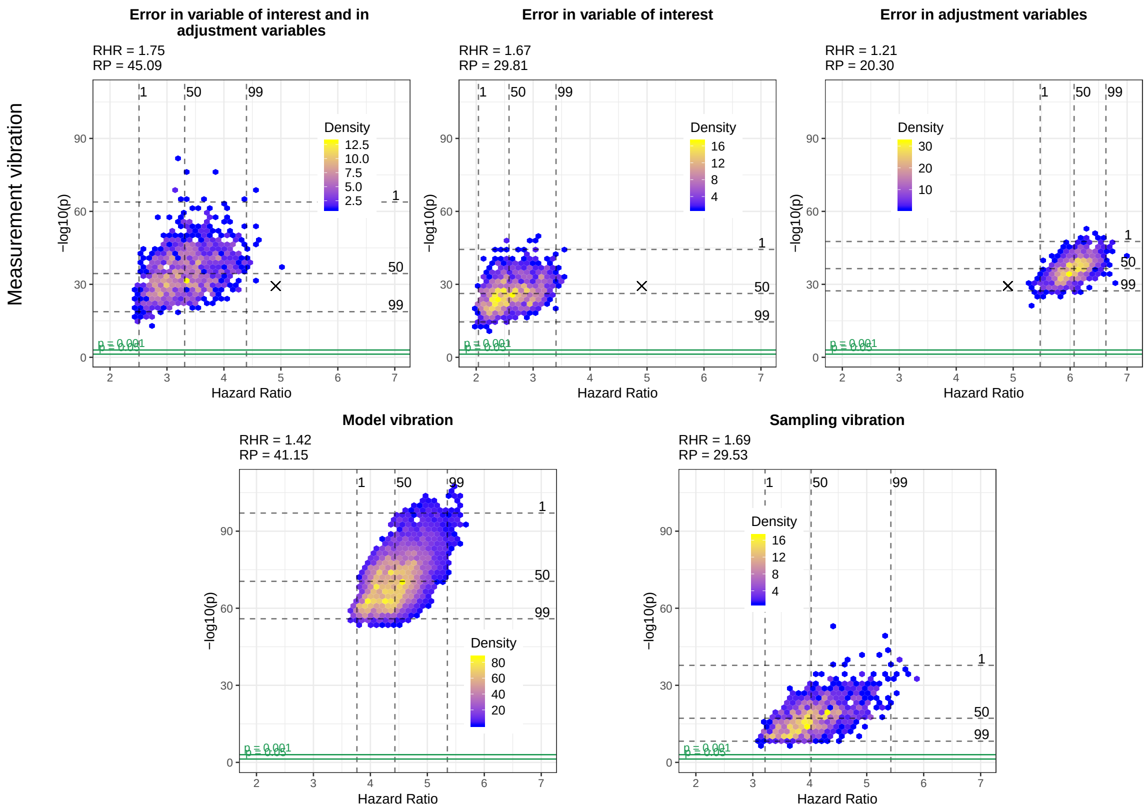

Figure S25: Volcano plots for different types of vibration and different scenarios of measurement vibration when **age** is the variable of interest. The summary measures RHR and RP indicate relative hazard ratios and relative p-values, respectively. The black cross in the top panel indicates the model without measurement error.

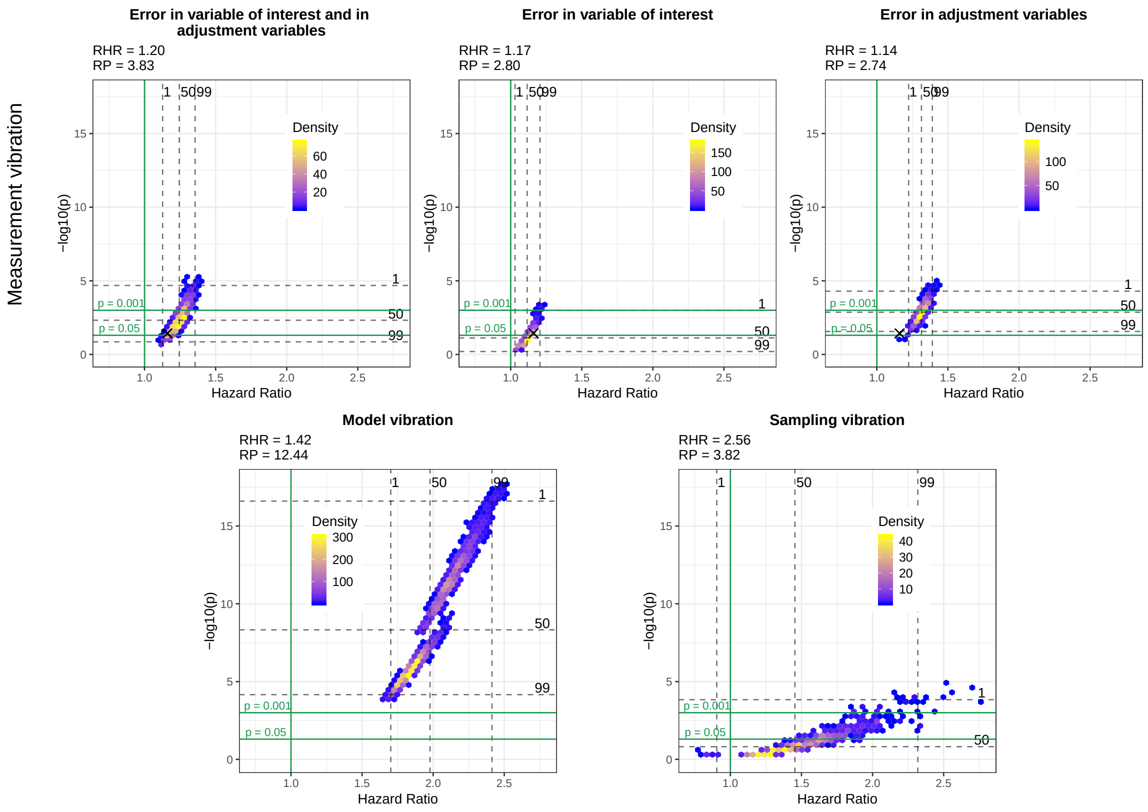

Figure S26: Volcano plots for different types of vibration and different scenarios of measurement vibration when **passive smoking** is the variable of interest. The summary measures RHR and RP indicate relative hazard ratios and relative p-values, respectively. The black cross in the top panel indicates the model without measurement error.

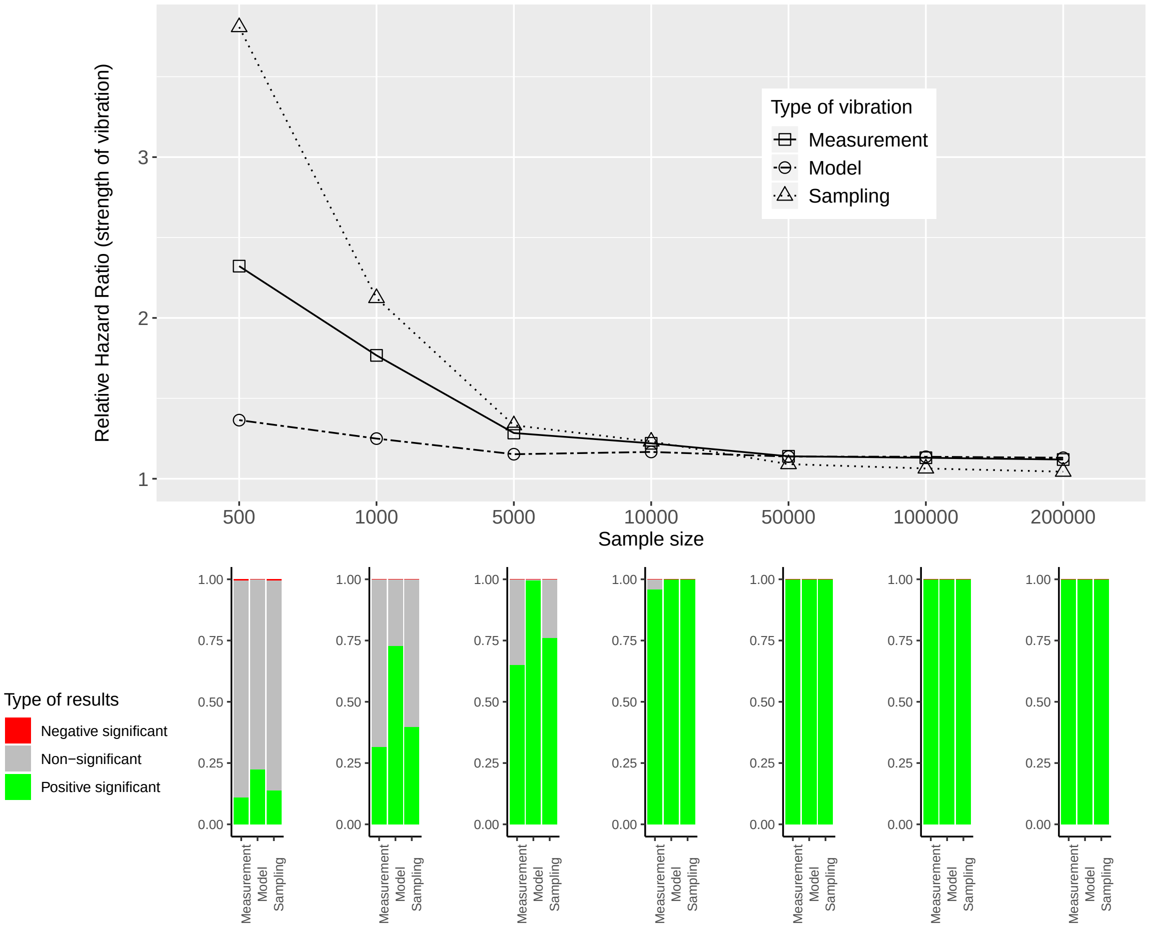

Figure S27: Measurement, model, and sampling vibration for different sample sizes (top panel), and bar plots visualizing the type of results in terms of significance of estimated effects (bottom panel) for the association of **hypertension** with mortality.

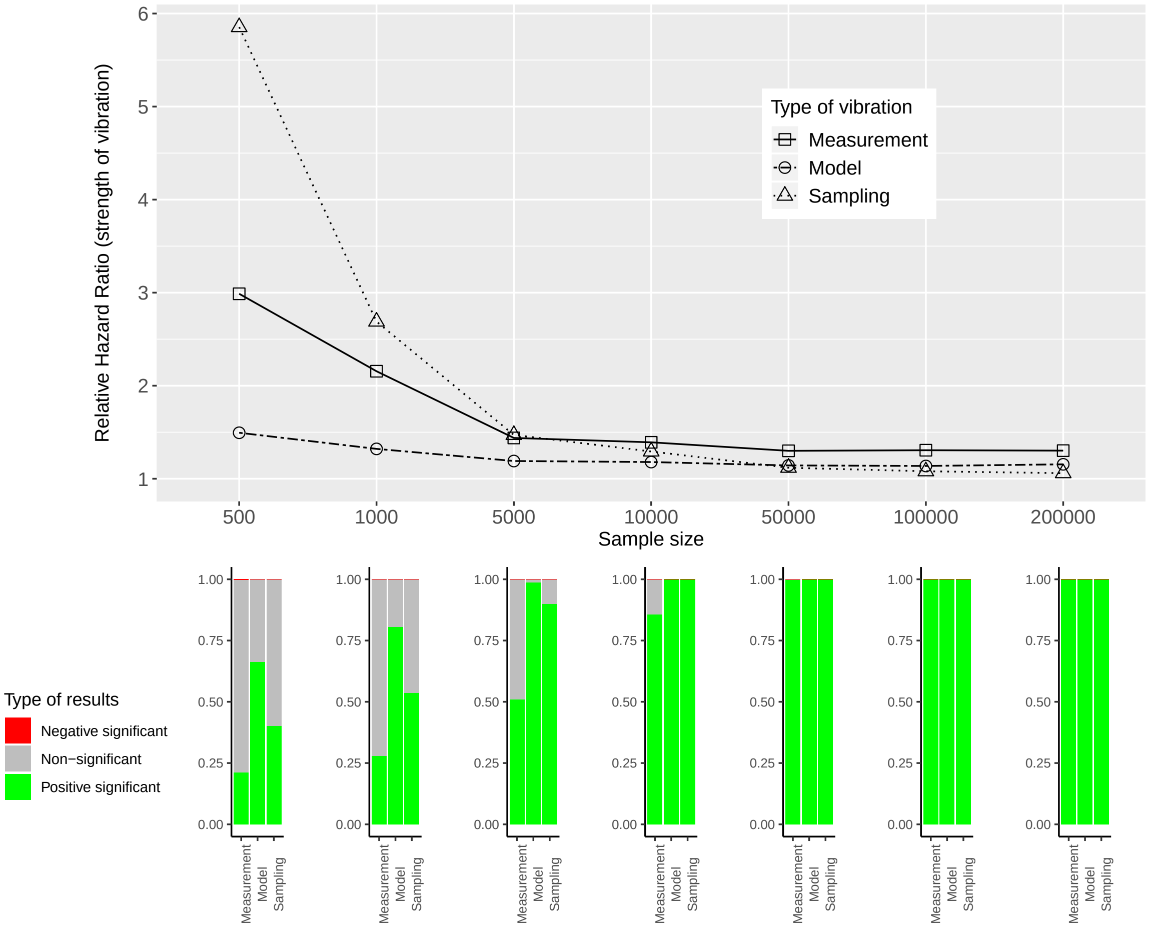

Figure S28: Measurement, model, and sampling vibration for different sample sizes (top panel), and bar plots visualizing the type of results in terms of significance of estimated effects (bottom panel) for the association of **any cancer** with mortality.

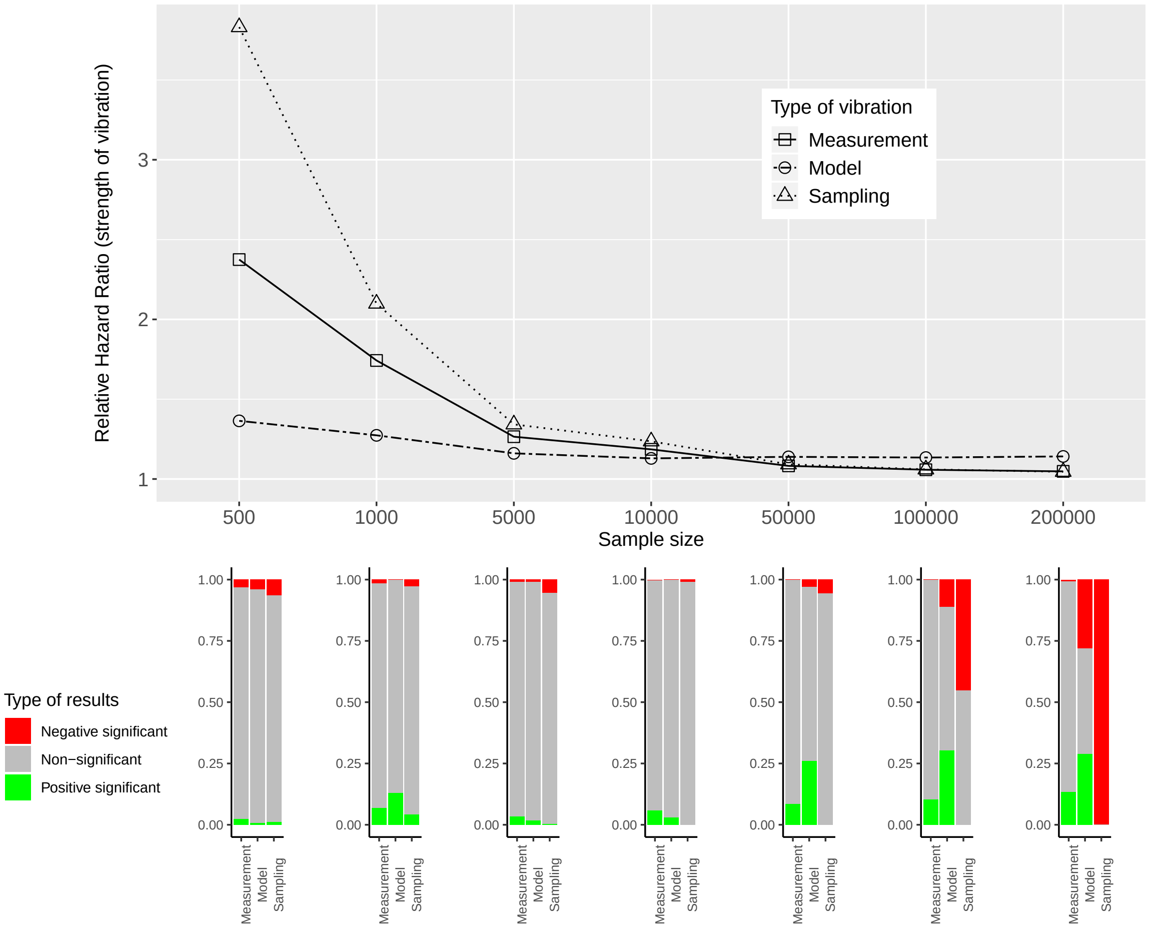

Figure S29: Measurement, model, and sampling vibration for different sample sizes (top panel), and bar plots visualizing the type of results in terms of significance of estimated effects (bottom panel) for the association of **family history of heart disease** with mortality.

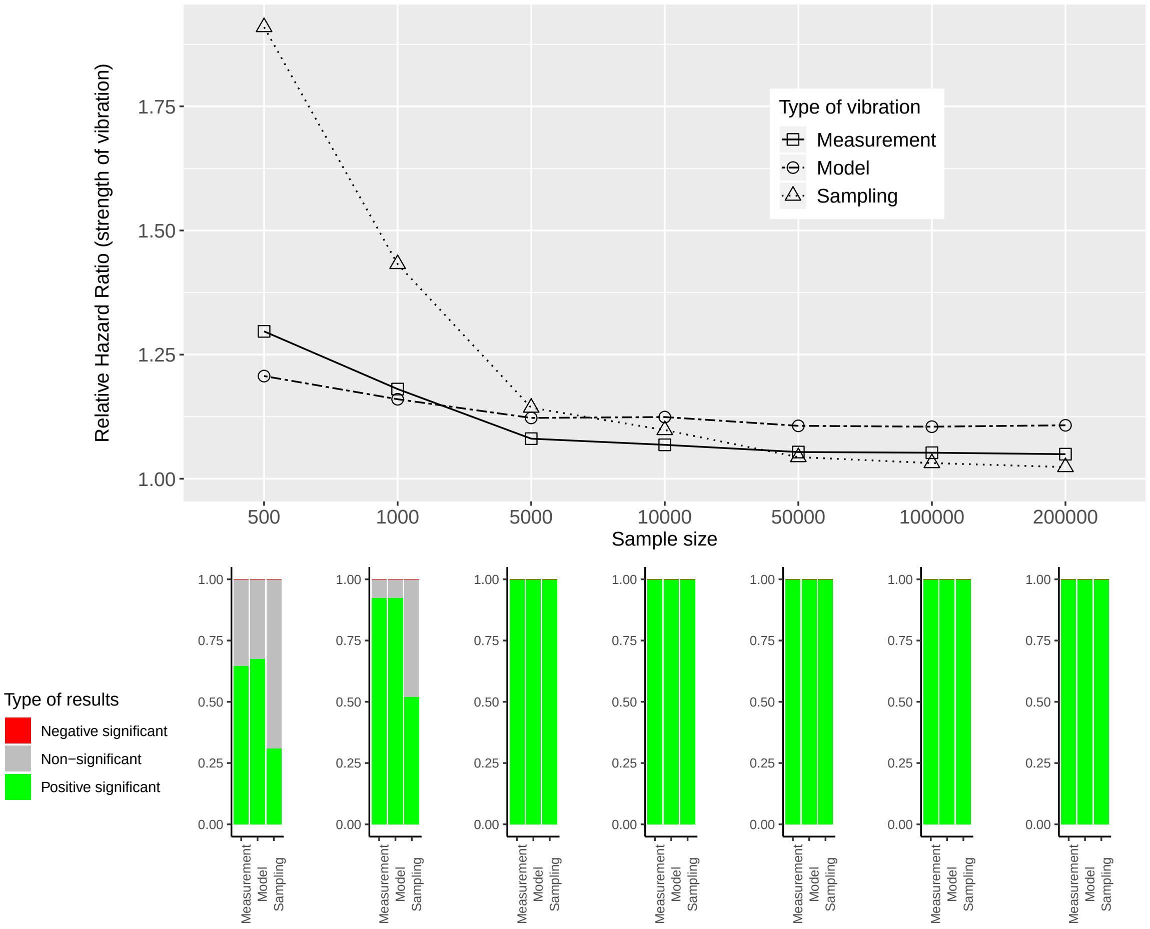

Figure S30: Measurement, model, and sampling vibration for different sample sizes (top panel), and bar plots visualizing the type of results in terms of significance of estimated effects (bottom panel) for the association of **segmented neutrophils number** with mortality.

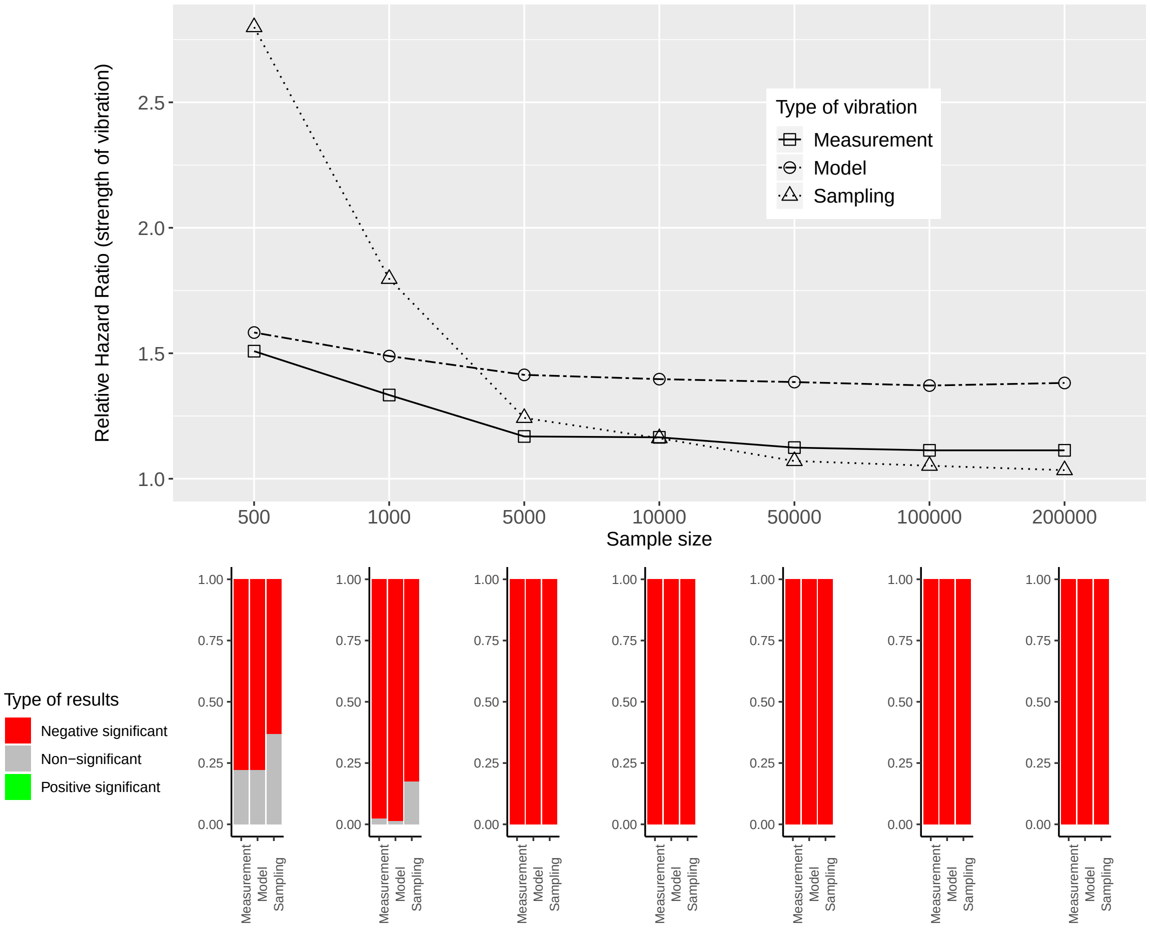

Figure S31: Measurement, model, and sampling vibration for different sample sizes (top panel), and bar plots visualizing the type of results in terms of significance of estimated effects (bottom panel) for the association of **maximal calf circumference** with mortality.

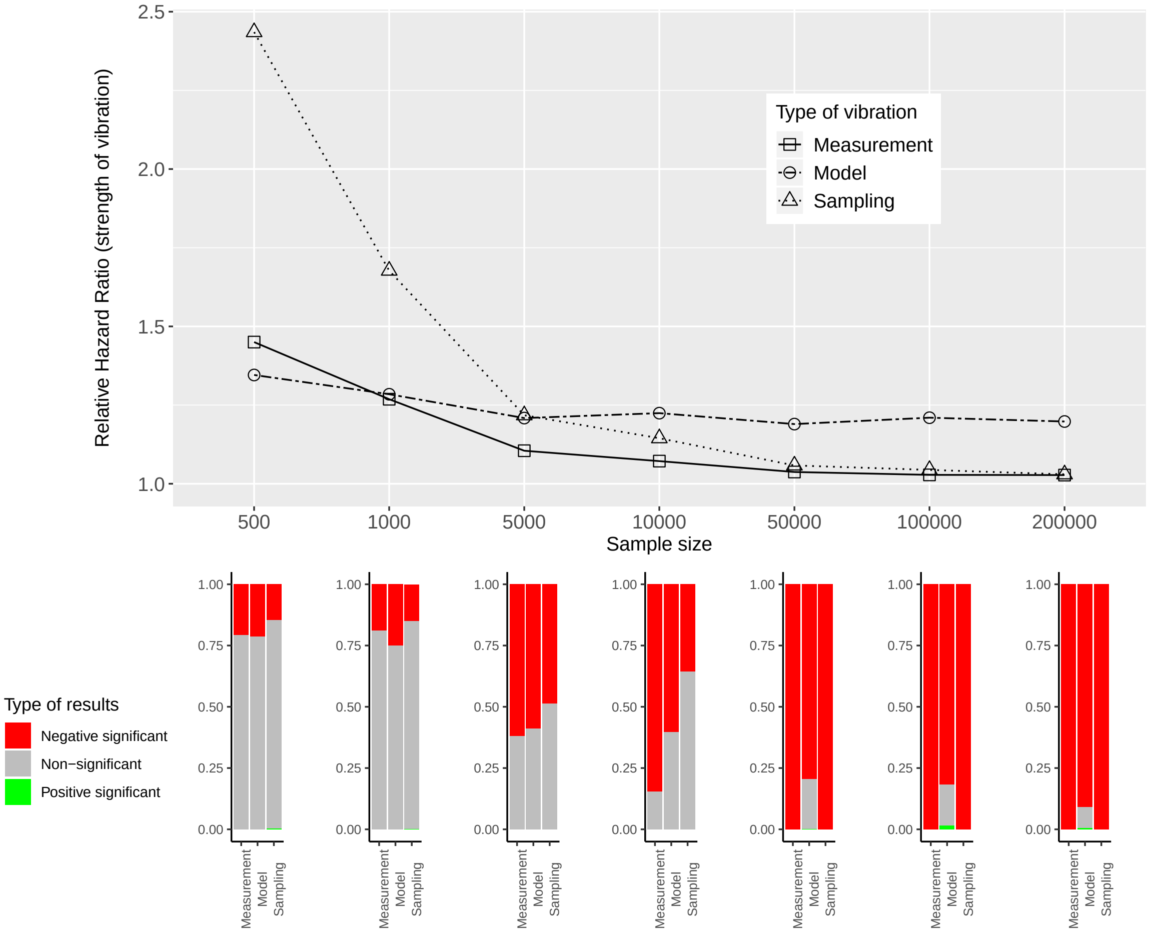

Figure S32: Measurement, model, and sampling vibration for different sample sizes (top panel), and bar plots visualizing the type of results in terms of significance of estimated effects (bottom panel) for the association of **standing height** with mortality.

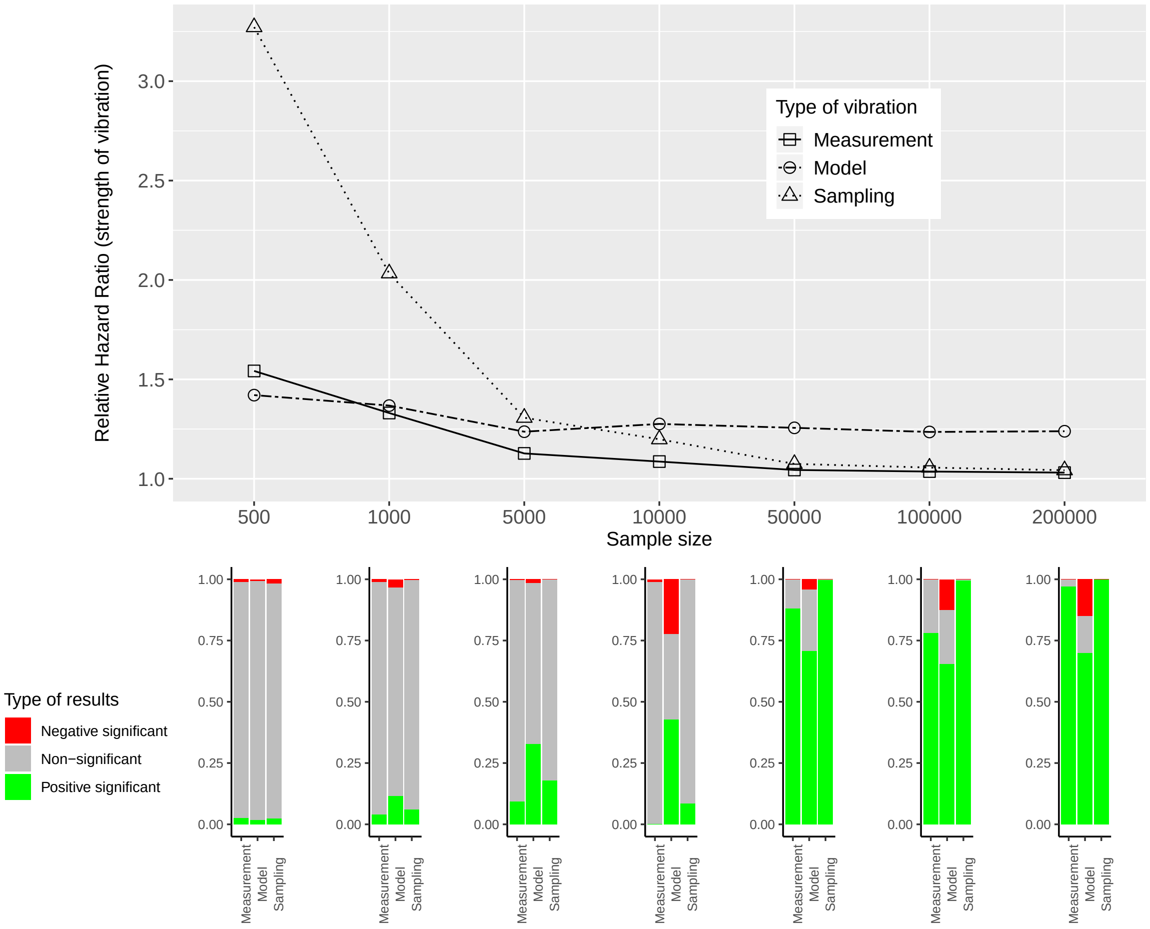

Figure S33: Measurement, model, and sampling vibration for different sample sizes (top panel), and bar plots visualizing the type of results in terms of significance of estimated effects (bottom panel) for the association of **waist circumference** with mortality.

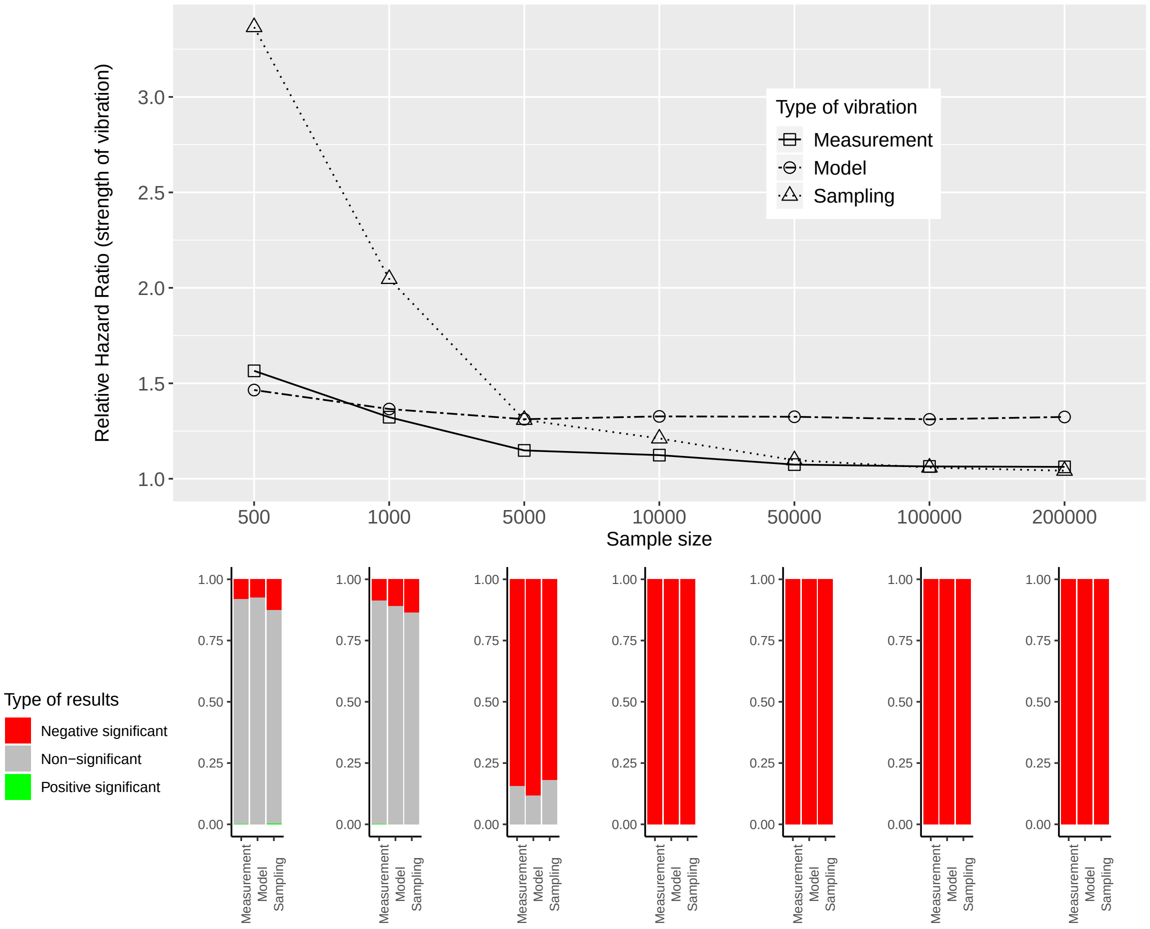

Figure S34: Measurement, model, and sampling vibration for different sample sizes (top panel), and bar plots visualizing the type of results in terms of significance of estimated effects (bottom panel) for the association of **weight** with mortality.

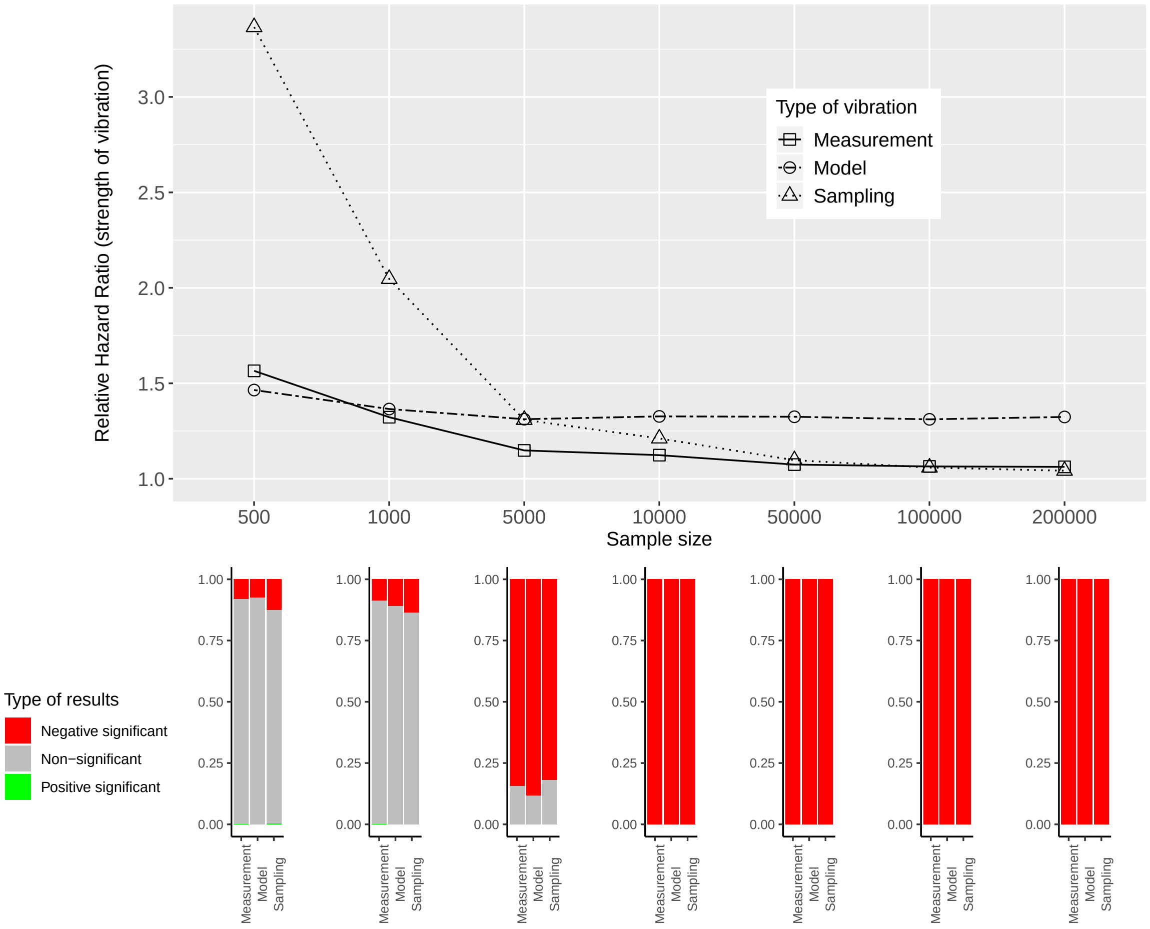

Figure S35: Measurement, model, and sampling vibration for different sample sizes (top panel), and bar plots visualizing the type of results in terms of significance of estimated effects (bottom panel) for the association of **60 sec. pulse** with mortality.

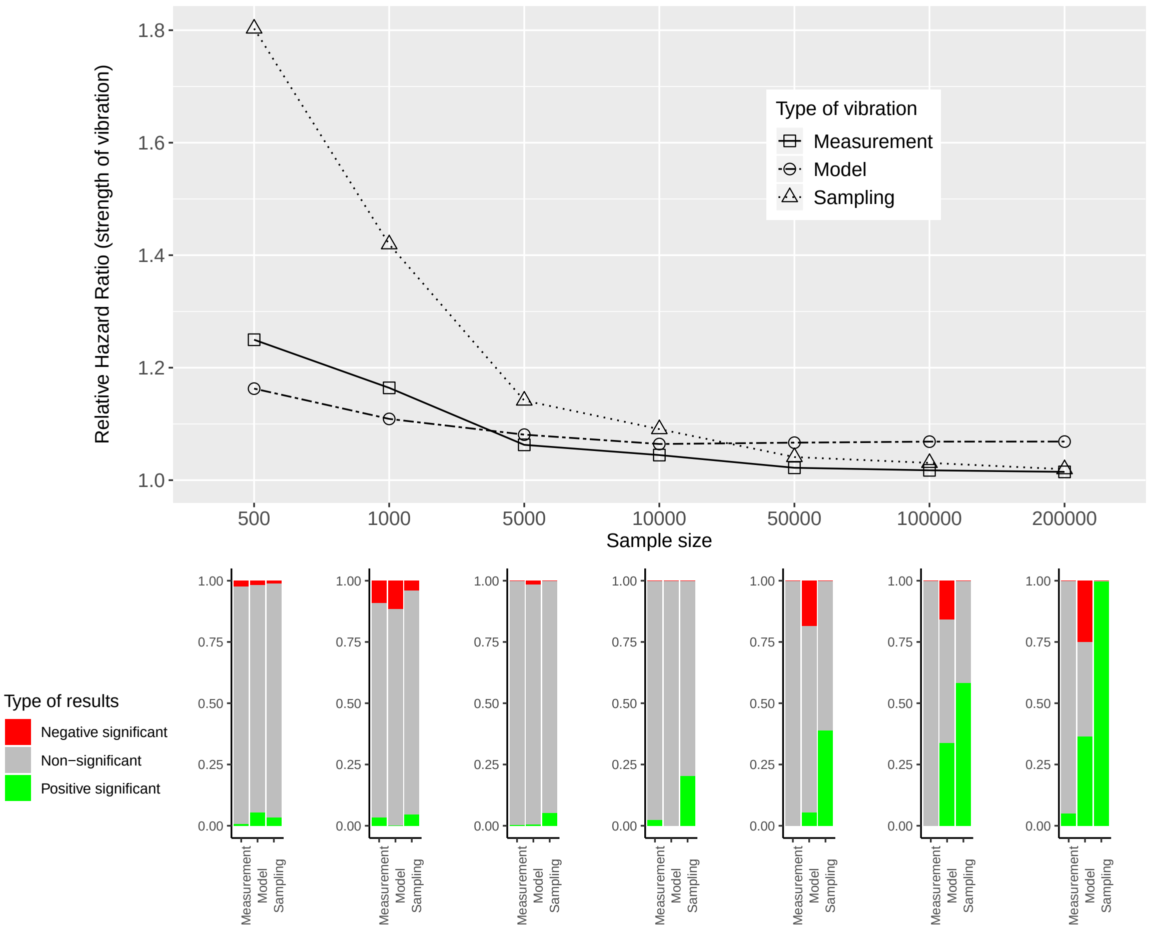

Figure S36: Measurement, model, and sampling vibration for different sample sizes (top panel), and bar plots visualizing the type of results in terms of significance of estimated effects (bottom panel) for the association of **number of dietary supplements taken** with mortality.

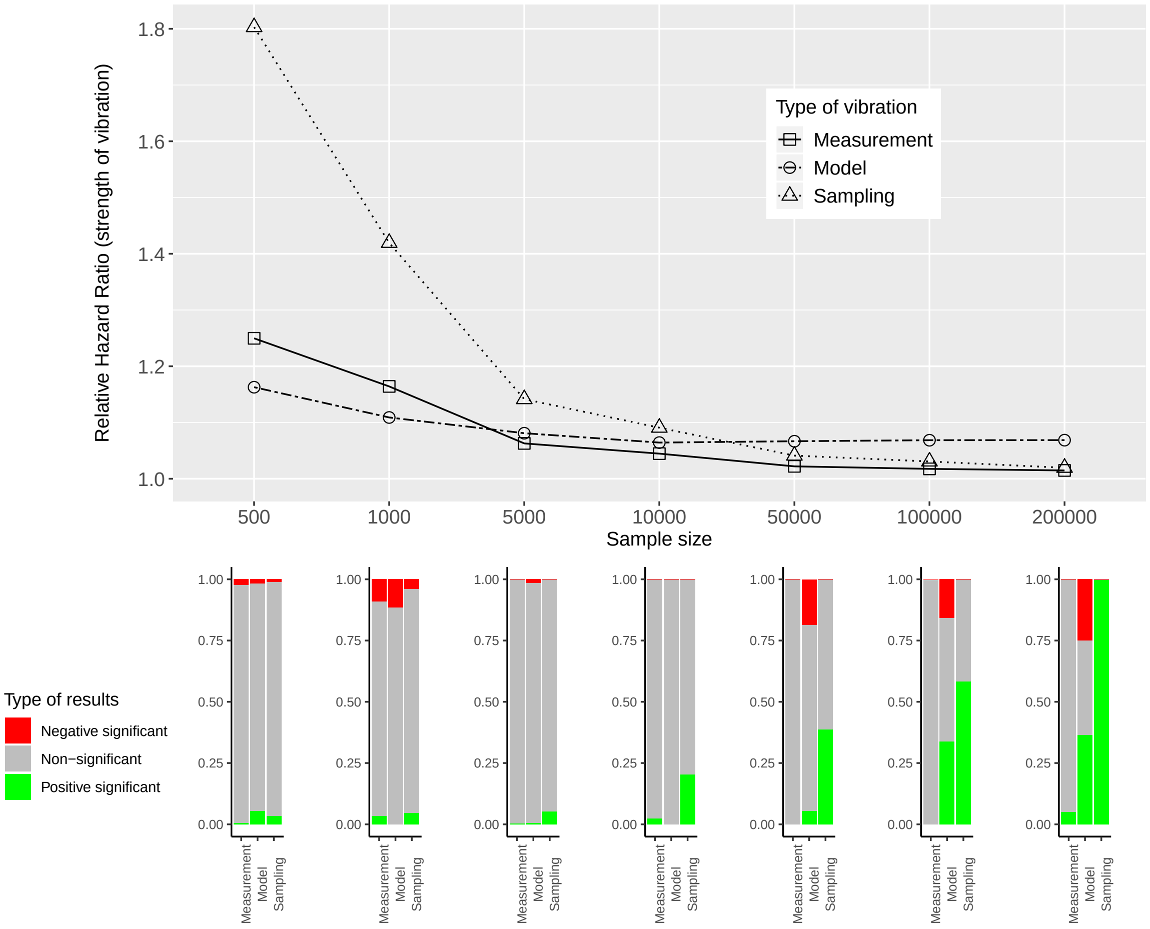

Figure S37: Measurement, model, and sampling vibration for different sample sizes (top panel), and bar plots visualizing the type of results in terms of significance of estimated effects (bottom panel) for the association of **hepatitis a antibody** with mortality.

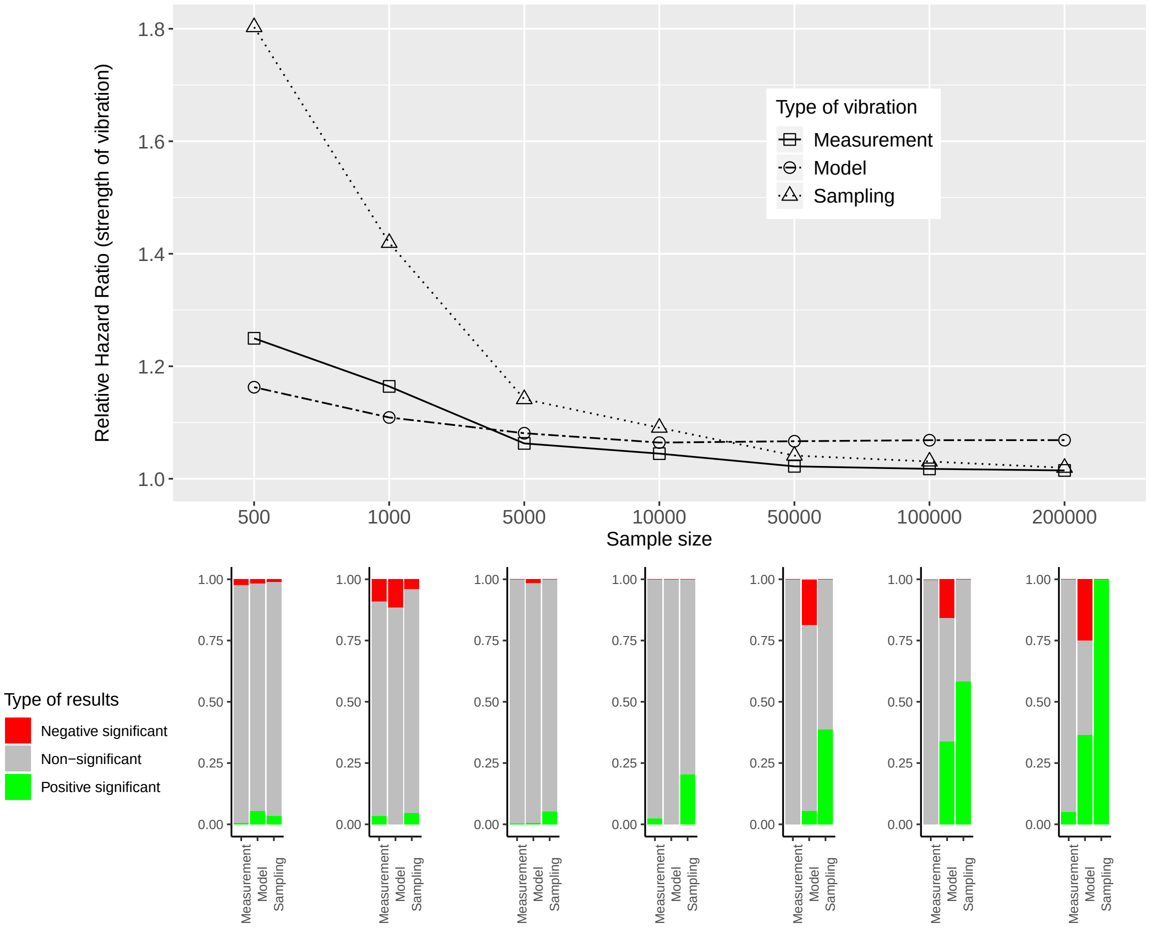

Figure S38: Measurement, model, and sampling vibration for different sample sizes (top panel), and bar plots visualizing the type of results in terms of significance of estimated effects (bottom panel) for the association of **hepatitis B core antibody** with mortality.

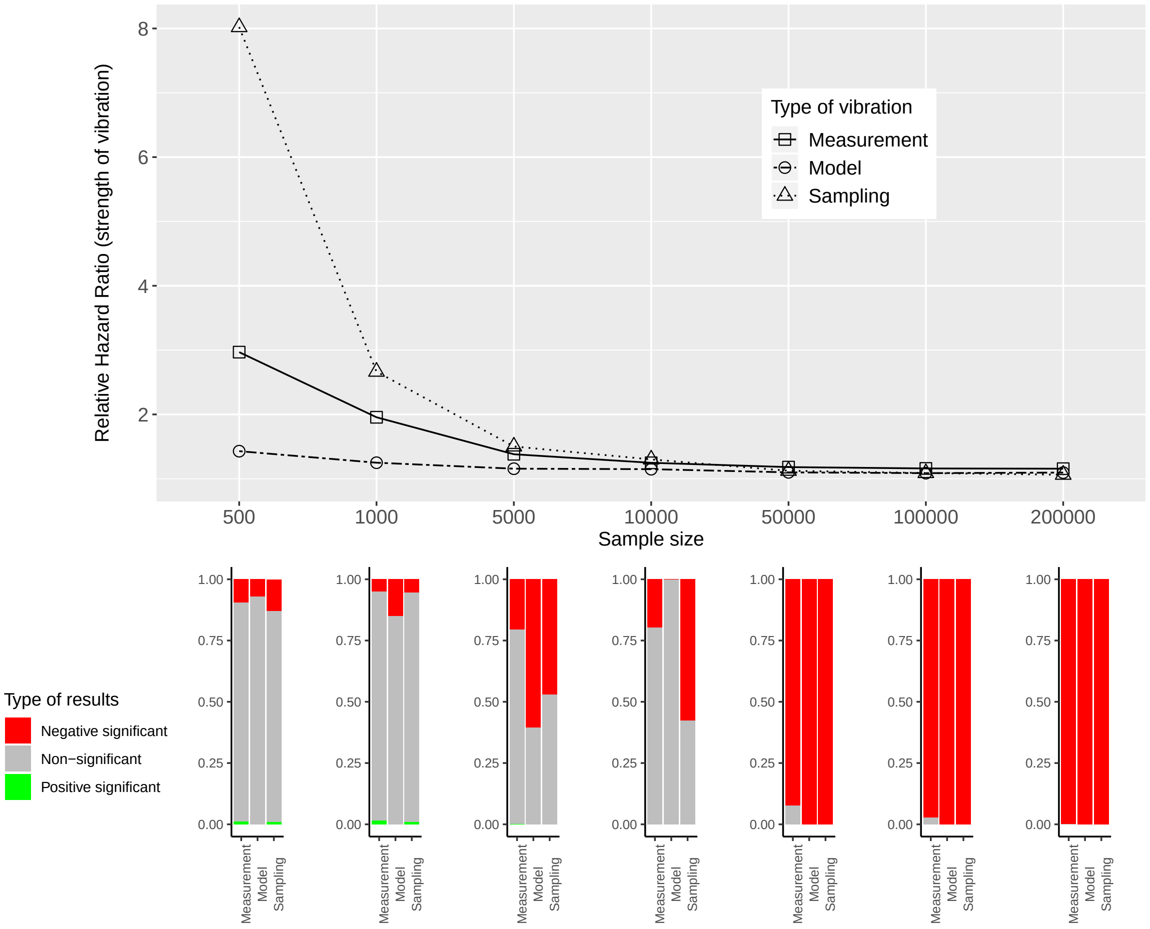

Figure S39: Measurement, model, and sampling vibration for different sample sizes (top panel), and bar plots visualizing the type of results in terms of significance of estimated effects (bottom panel) for the association of **hepatitis B surface antibody** with mortality.

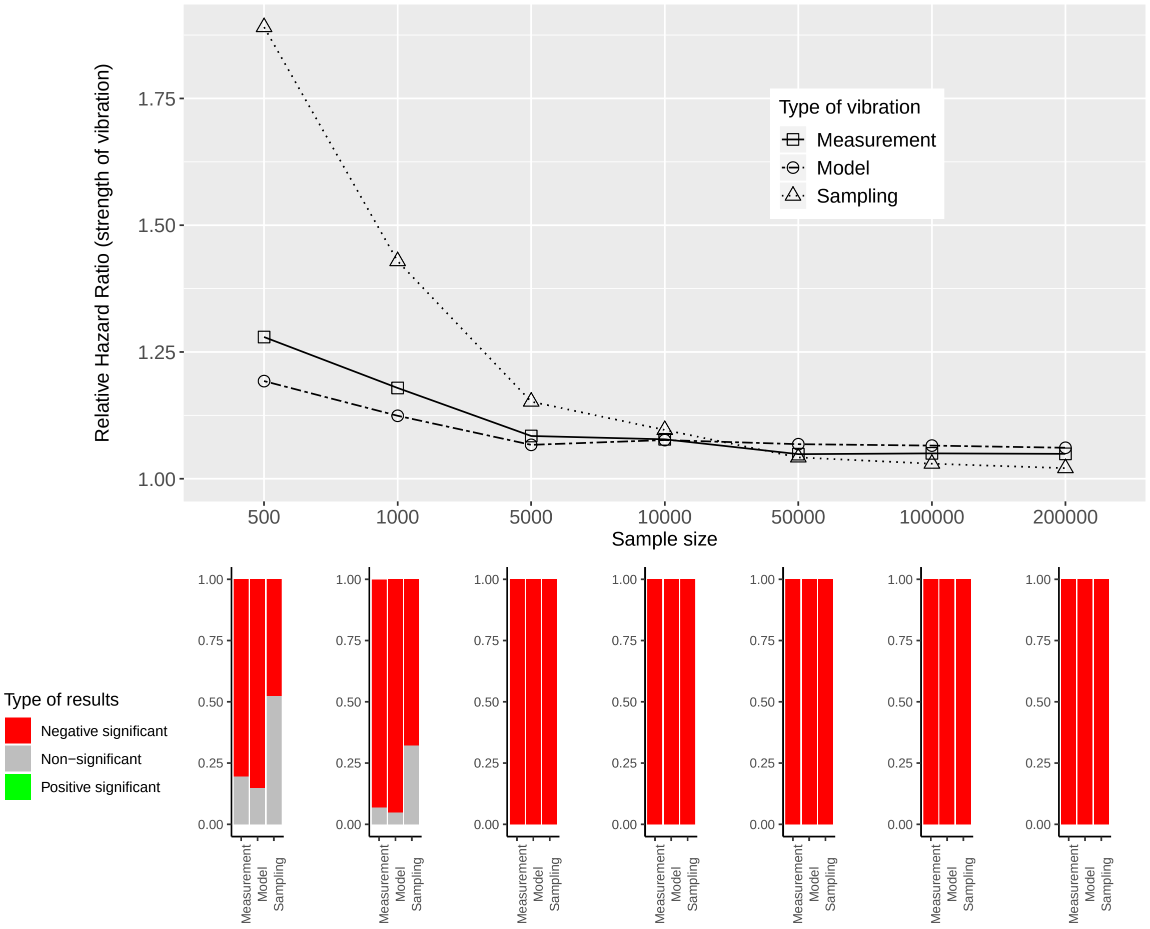

Figure S40: Measurement, model, and sampling vibration for different sample sizes (top panel), and bar plots visualizing the type of results in terms of significance of estimated effects (bottom panel) for the association of **lymphocyte percent** with mortality.

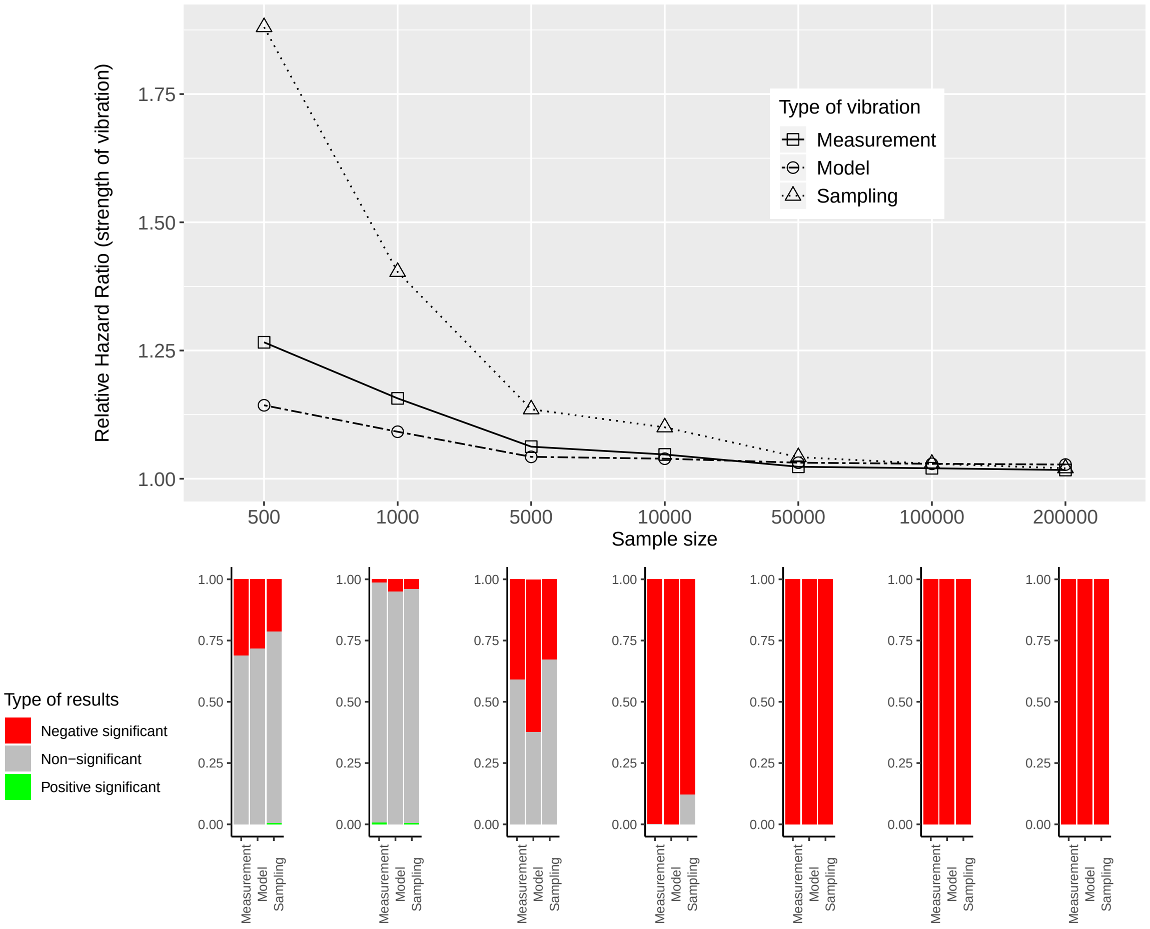

Figure S41: Measurement, model, and sampling vibration for different sample sizes (top panel), and bar plots visualizing the type of results in terms of significance of estimated effects (bottom panel) for the association of **mean platelet volume** with mortality.

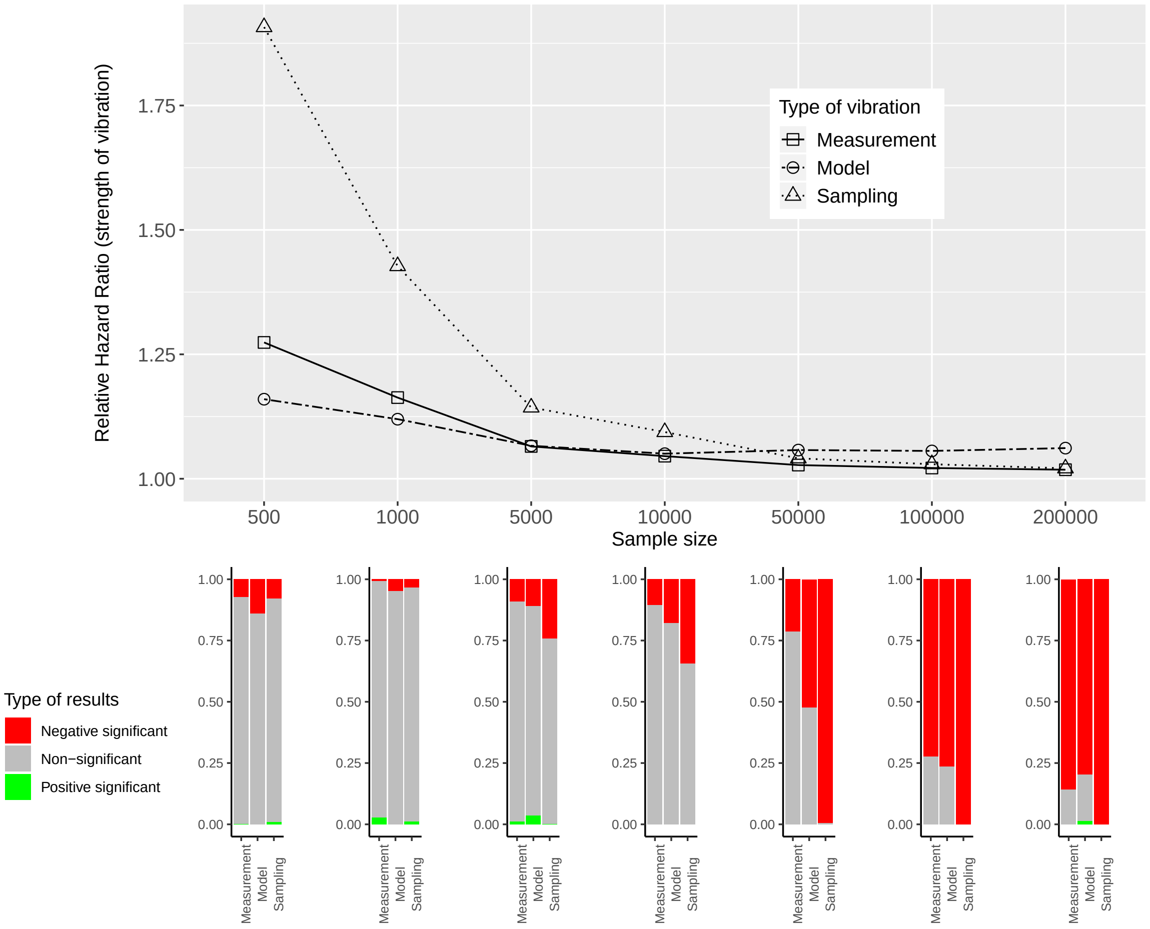

Figure S42: Measurement, model, and sampling vibration for different sample sizes (top panel), and bar plots visualizing the type of results in terms of significance of estimated effects (bottom panel) for the association of **calcium** with mortality.

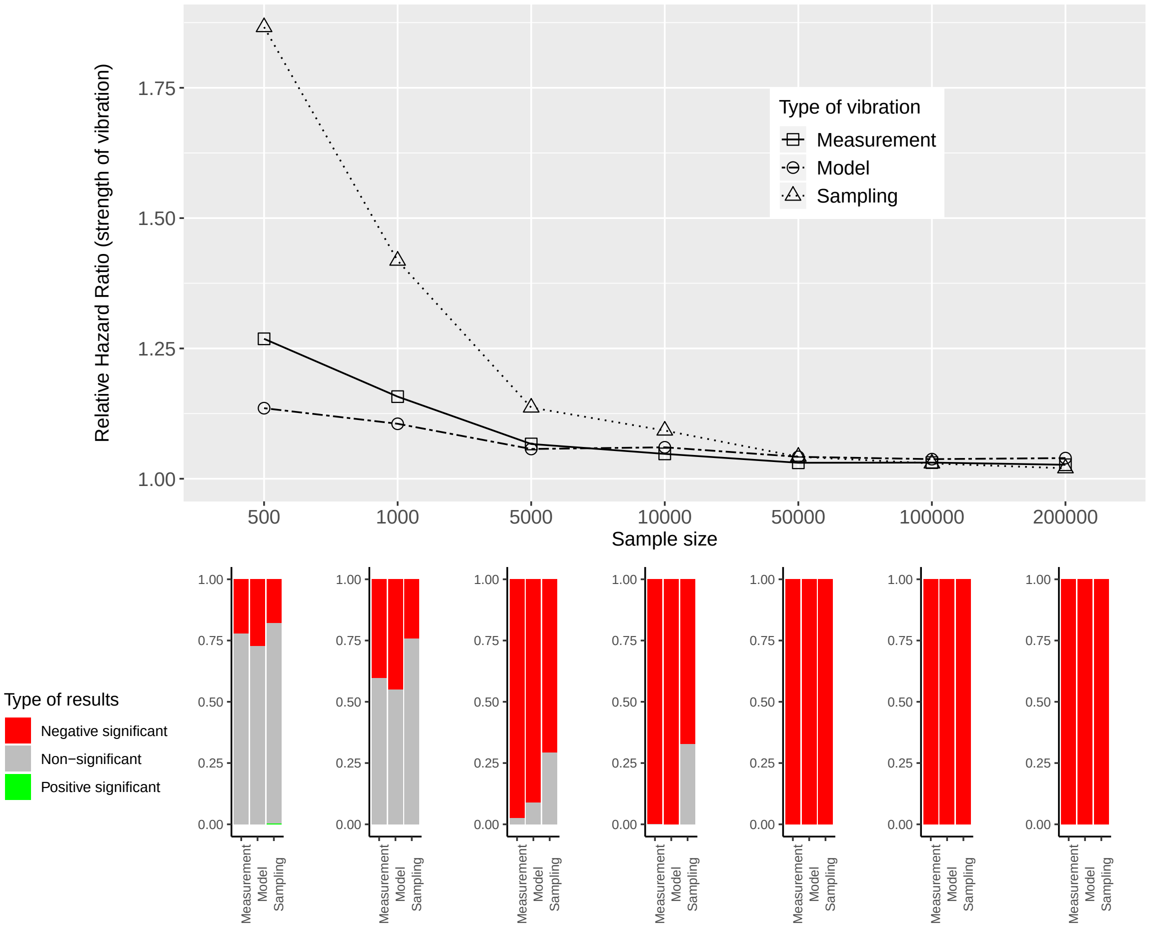

Figure S43: Measurement, model, and sampling vibration for different sample sizes (top panel), and bar plots visualizing the type of results in terms of significance of estimated effects (bottom panel) for the association of **sodium** with mortality.

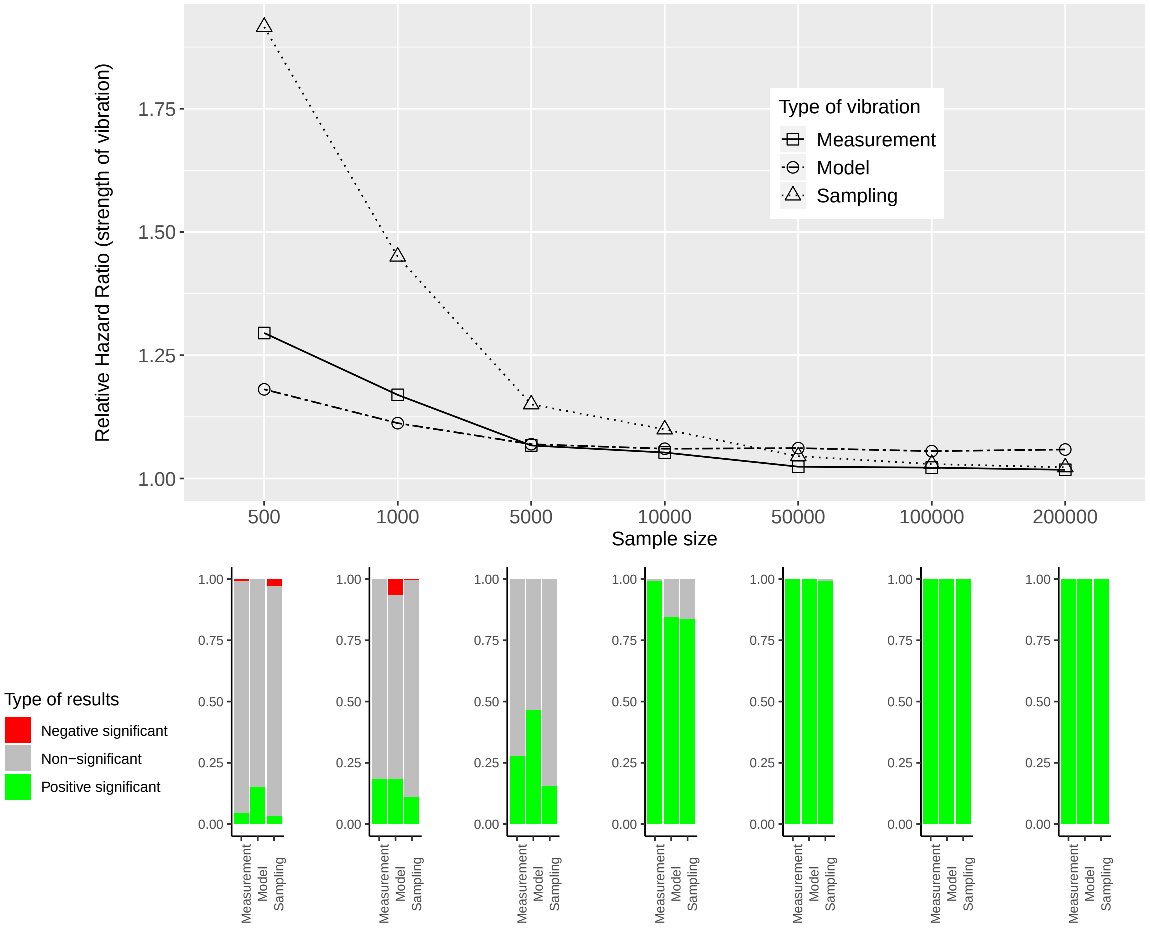

Figure S44: Measurement, model, and sampling vibration for different sample sizes (top panel), and bar plots visualizing the type of results in terms of significance of estimated effects (bottom panel) for the association of **osmolality** with mortality.

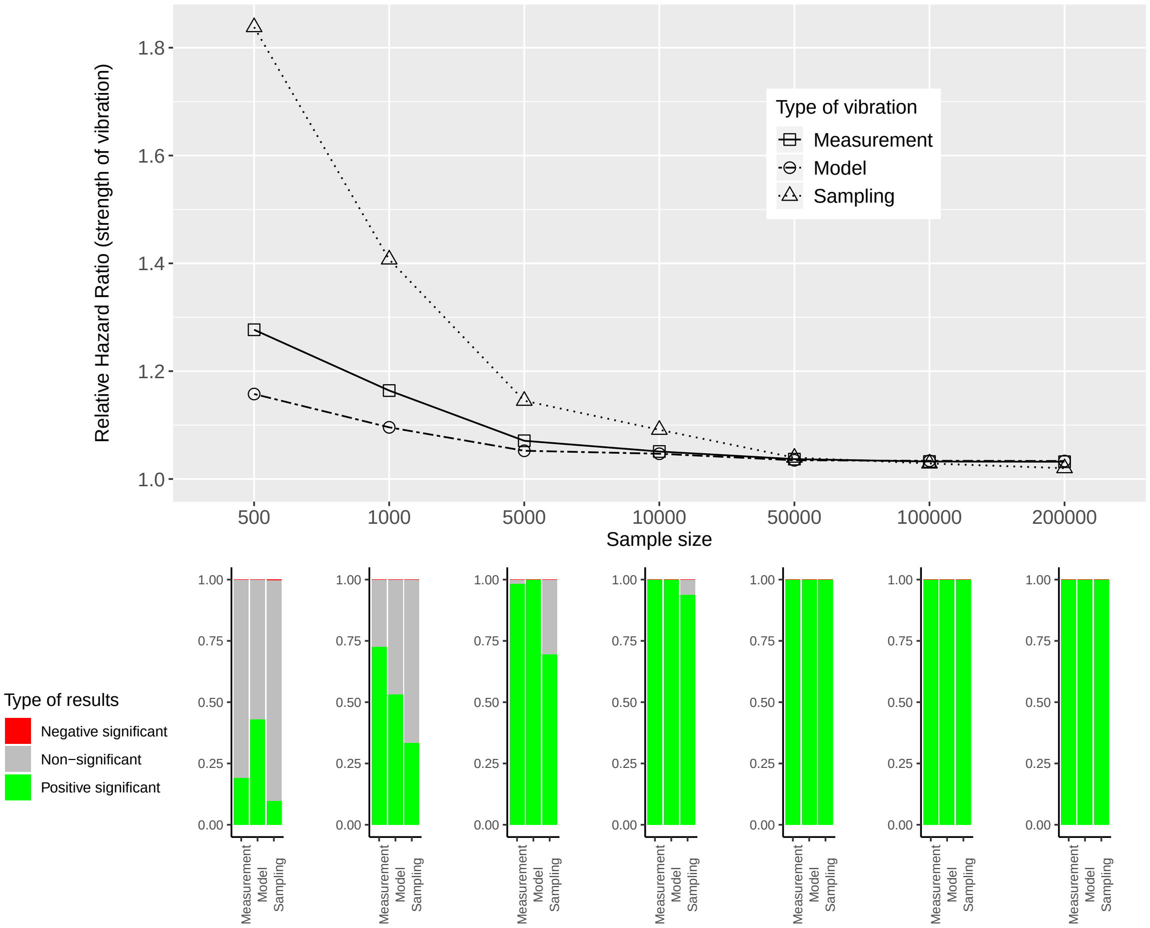

Figure S45: Measurement, model, and sampling vibration for different sample sizes (top panel), and bar plots visualizing the type of results in terms of significance of estimated effects (bottom panel) for the association of **phosphorus** with mortality.

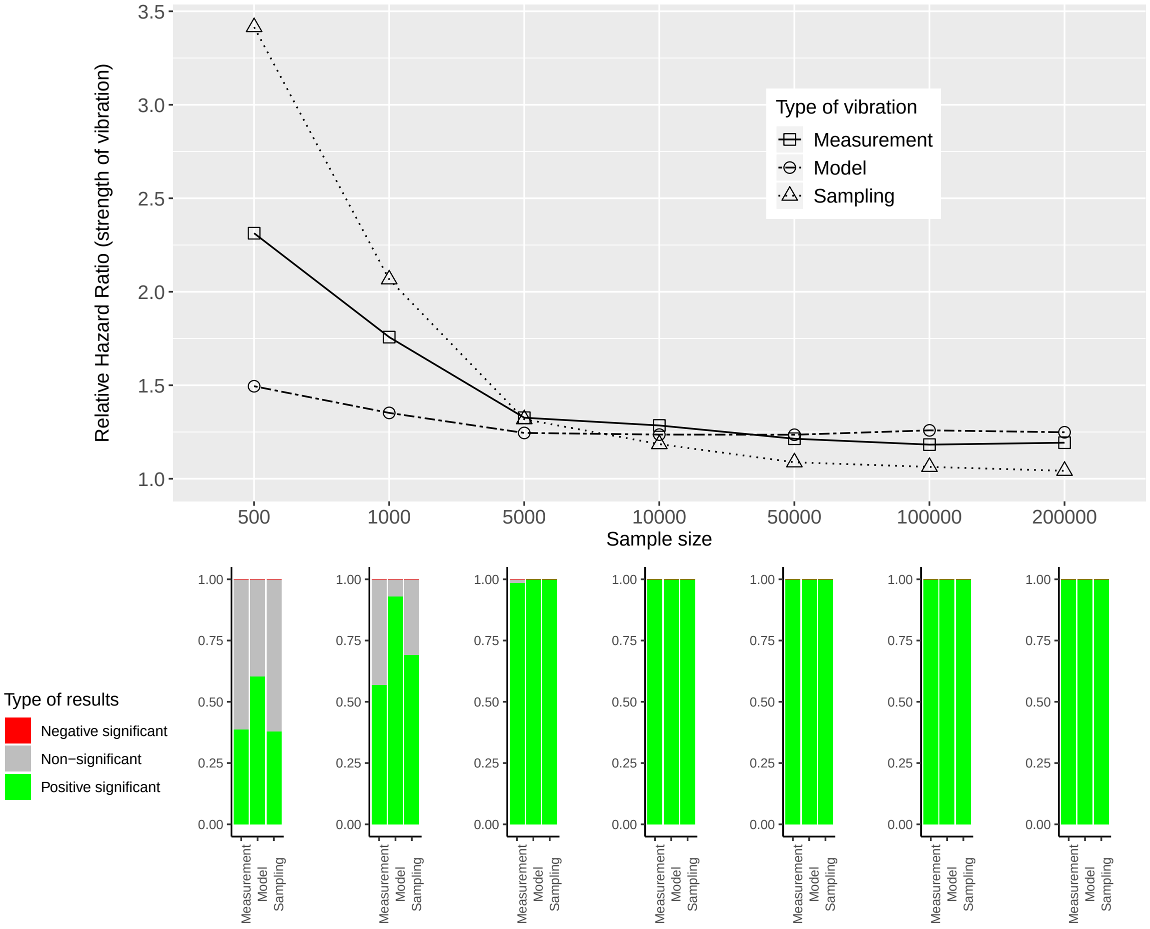

Figure S46: Measurement, model, and sampling vibration for different sample sizes (top panel), and bar plots visualizing the type of results in terms of significance of estimated effects (bottom panel) for the association of **sex** with mortality.

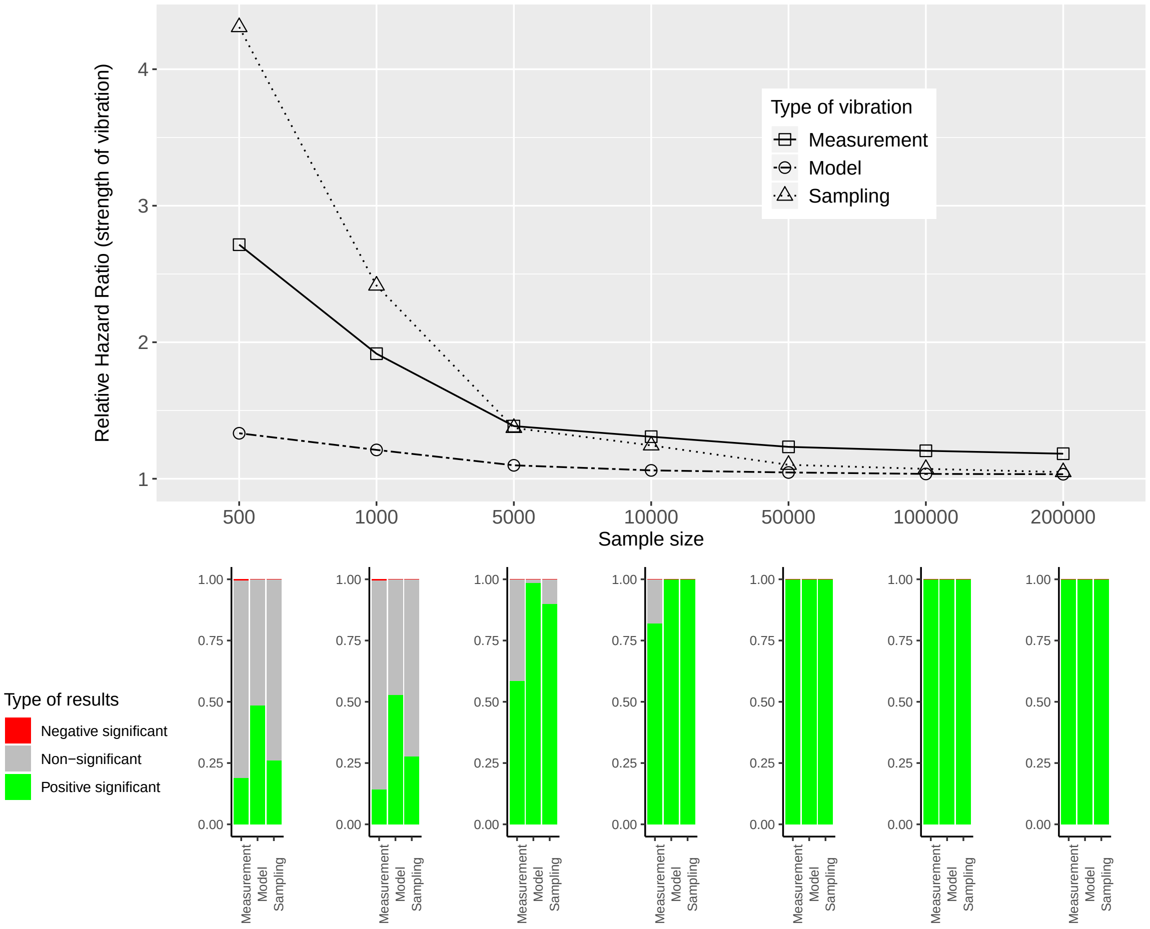

Figure S47: Measurement, model, and sampling vibration for different sample sizes (top panel), and bar plots visualizing the type of results in terms of significance of estimated effects (bottom panel) for the association of **pest control** with mortality.

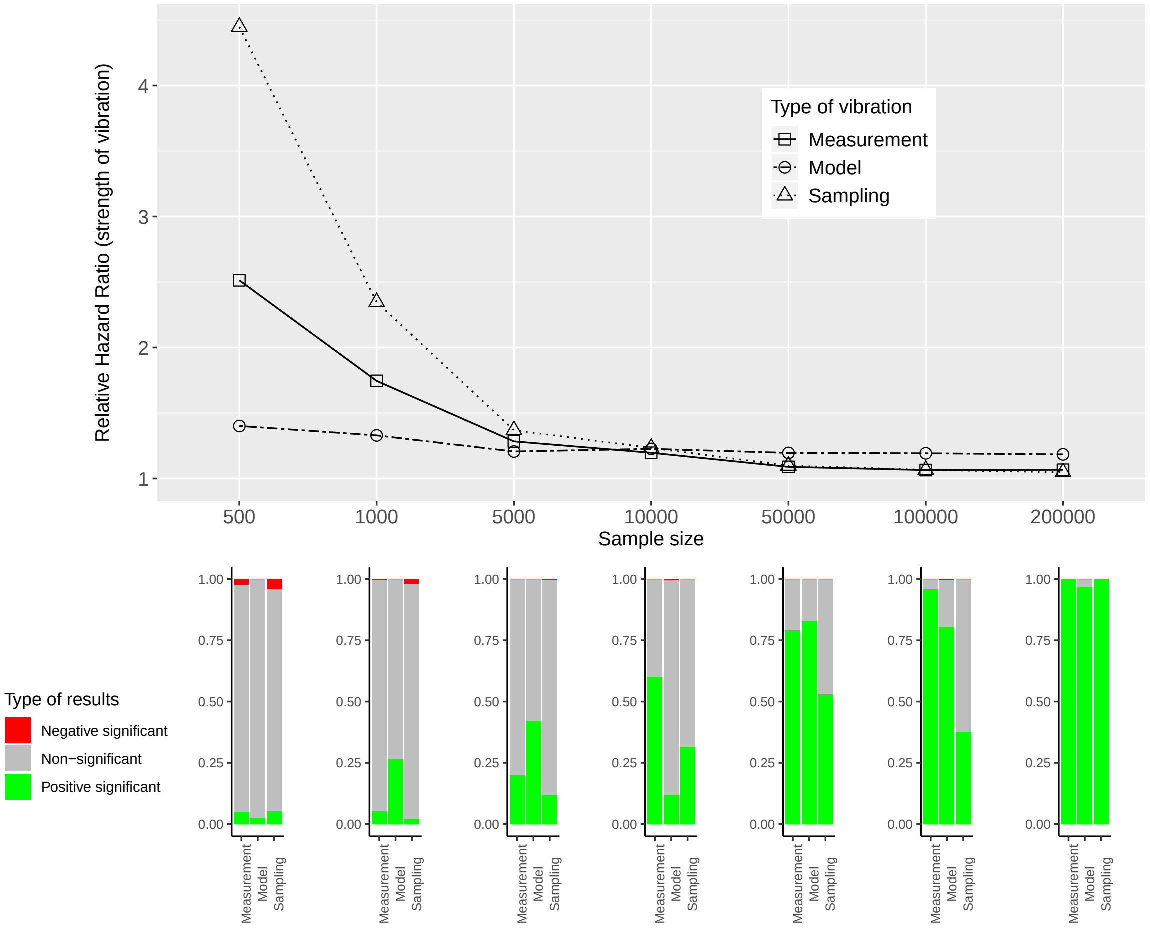

Figure S48: Measurement, model, and sampling vibration for different sample sizes (top panel), and bar plots visualizing the type of results in terms of significance of estimated effects (bottom panel) for the association of **pneumonia** with mortality.

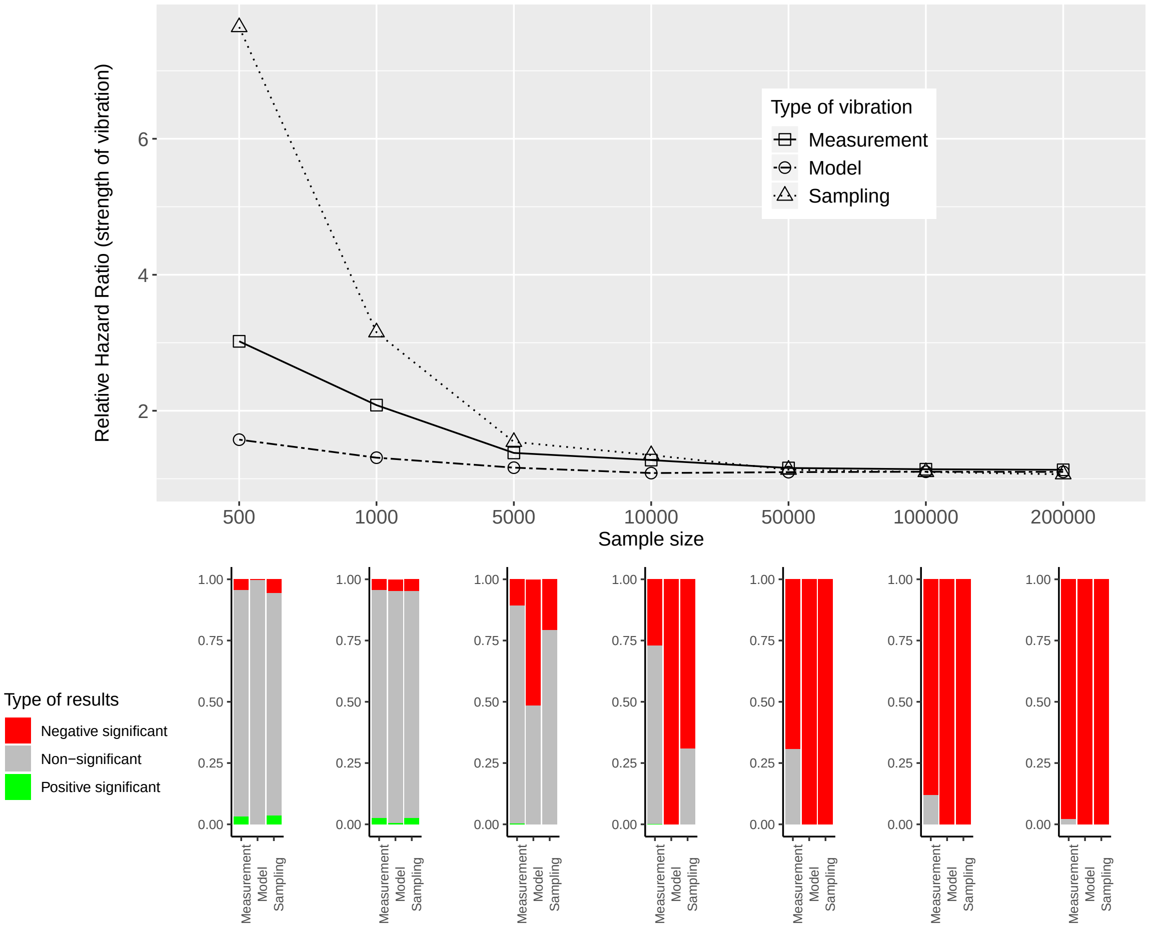

Figure S49: Measurement, model, and sampling vibration for different sample sizes (top panel), and bar plots visualizing the type of results in terms of significance of estimated effects (bottom panel) for the association of **private water source** with mortality.

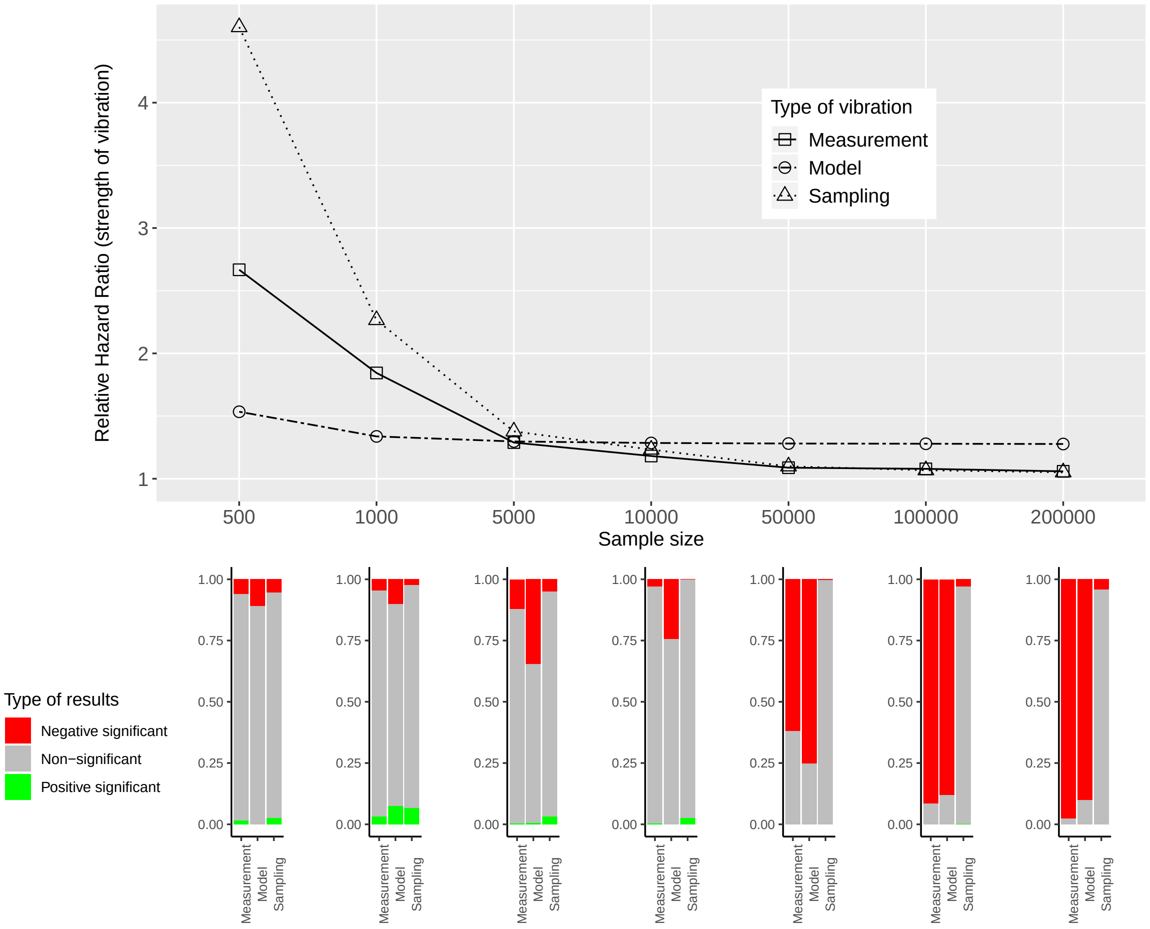

Figure S50: Measurement, model, and sampling vibration for different sample sizes (top panel), and bar plots visualizing the type of results in terms of significance of estimated effects (bottom panel) for the association of **water treatment** with mortality.

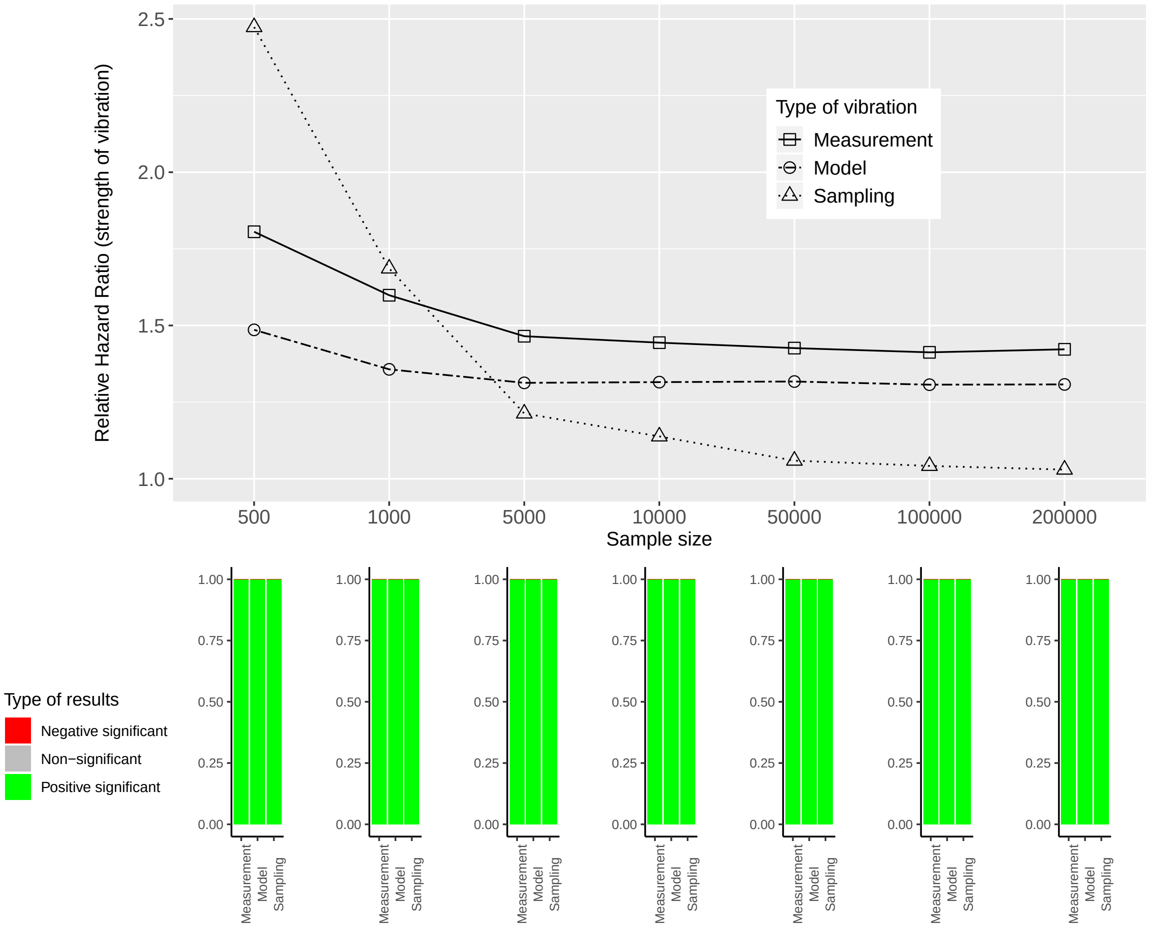

Figure S51: Measurement, model, and sampling vibration for different sample sizes (top panel), and bar plots visualizing the type of results in terms of significance of estimated effects (bottom panel) for the association of **age** with mortality.

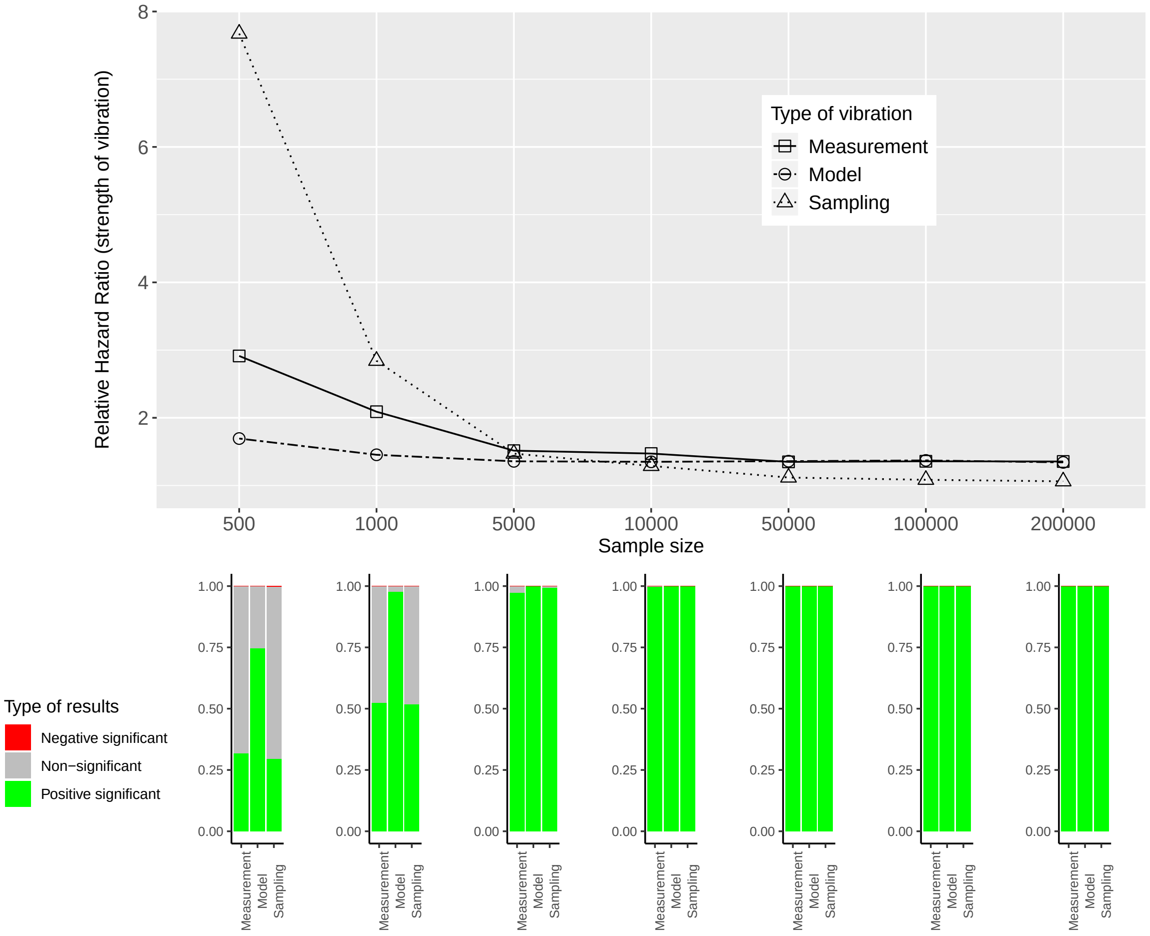

Figure S52: Measurement, model, and sampling vibration for different sample sizes (top panel), and bar plots visualizing the type of results in terms of significance of estimated effects (bottom panel) for the association of **passive smoking** with mortality.
